# One-pot synthesis of tetrahydropyrimidinecarboxamides enabling *in vitro* anticancer activity: a combinative study with clinically relevant brain-penetrant drugs†

**DOI:** 10.1039/d4ra04171b

**Published:** 2024-08-27

**Authors:** Dipti B. Upadhyay, Joaquina Nogales, Jaydeep A. Mokariya, Ruturajsinh M. Vala, Vasudha Tandon, Sourav Banerjee, Hitendra M. Patel

**Affiliations:** a Department of Chemistry, Sardar Patel University Vallabh Vidyanagar Gujarat India hm_patel@spuvvn.edu; b Division of Cancer Research, School of Medicine, University of Dundee Dundee DD1 9SY UK s.y.banerjee@dundee.ac.uk

## Abstract

In this study, we describe a one-pot three-component synthesis of bioactive tetrahydopyrimidinecarboxamide derivatives employing lanthanum triflate as a catalyst. Out of the synthesized compounds, 4f had the most potent anti-cancer activity and impeded cell cycle progression effectively. Anti-cancer bioactivity was observed in 4f against liver, breast, and lung cancers as well as primary patient-derived glioblastoma cell lines. Compound 4f effectively inhibited the 3D neurosphere formation in primary patient-derived glioma stem cells. Specifically, 4f exhibited synergistic cytotoxicity with the EGFR inhibitor that is the clinical epidermal growth factor receptor inhibitor osimertinib. 4f does not exhibit anti-kinase activity and is cytostatic in nature, and further work is needed to understand the true molecular target of 4f and its derivatives. Through our current work, we establish a promising tetrahydopyrimidinecarboxamide-based lead compound with anti-cancer activity, which may exhibit potent anti-cancer activity in combination with specific clinically relevant small molecule kinase inhibitors.

## Abstract 2

This study describe one-pot three-component synthesis of bioactive tetrahydopyrimidinecarboxamide employing La(OTf)_3_ as a catalyst. Compound, 4f had the most potent anti-cancer activity and impeded cell cycle progression effectively.

## Graphical abstract

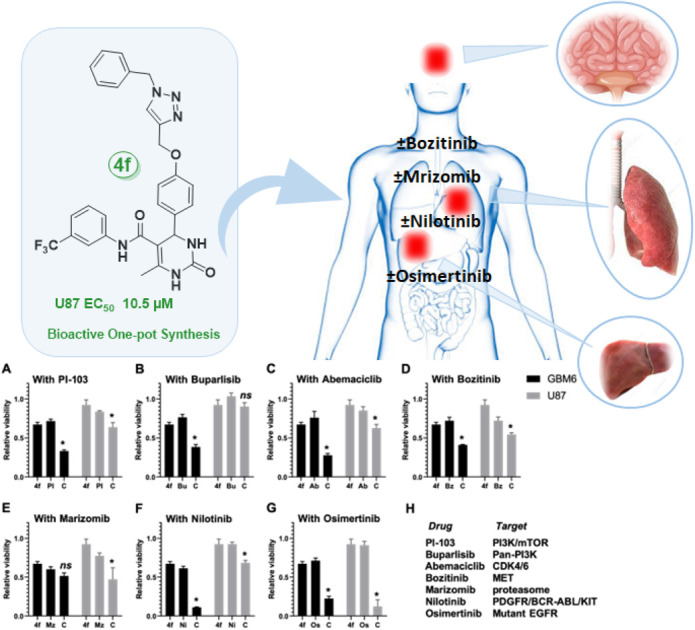

## 1 Introduction

**Fig. 1:**
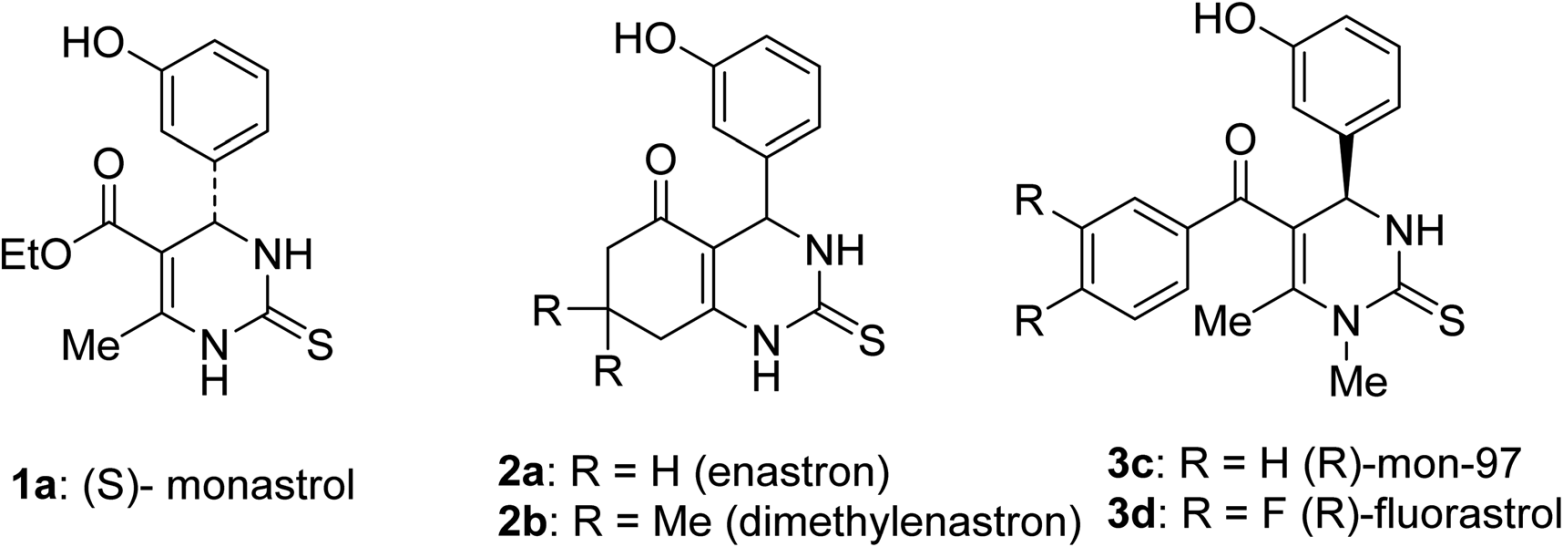
Bioactive dihydropyrimidinones (DHPMs).

Dihydropyrimidinones (DHPMs) produced *via* the Biginelli reaction have received significant attention owing to their diverse biological applications, such as acetylcholinesterase inhibitors,^1^ HIV-1 replication inhibitors,^2^ urease inhibitors,^3^ calcium channel blockers,^4^ antimicrobial agents,^5^ antioxidant agents,^6^ nonsteroidal RORα agonists,^7^ anti-cancer cytotoxic agents,^8^ and antitubercular agents.^9^ From this DHPM family of compounds, monastrol emerged as a key lead compound.^10,11^ Monastrol has significant anticancer properties (Fig. 1) and prevents metastasis by impeding the movement of kinesin spindle protein Eg5,^12^ which associates with microtubules of the mitotic spindle^13^ and engages in intracellular transport and cell division.^14^ Dihydropyrimidine derivatives allosterically inhibit Eg5-mediated microtubule organisation and drive the anti-metastatic activities.^15^ Monastrol was first observed to cause cell cycle disruption in Xenopus models^16^ and later monastrol and its derivatives were reported to have robust anti-proliferative activities against cancer cells.^17^ Multiple research groups have since developed several DHPMs that exhibited antiproliferative properties against other human solid tumour cell lines (Fig. 1).

In the current study, we wanted to utilise our long-standing successful strategy^11,18–25^ of utilising the Biginelli reaction to synthesize benzyloxy derivatives of DHPM to obtain new bioactive, anti-cancer compounds. The benzyloxyphenyl group has diverse medicinal properties, including antimicrobial,^26^ antitubercular,^27^ antifungal,^28^ and anticancer activities.^11,29–32^ Similarly, the incorporation of a 1,2,3-triazole moiety into various compounds has shown significant pharmacological potential, such as anticancer,^33–38^ antimicrobial,^33,39^ anti-inflammatory,^40^ antitubercular,^41^ anti-HIV,^42^ and antiviral effects.^33^ Studies have shown that 1,2,3-triazoles can exert anticancer effects through various mechanisms, such as enzyme inhibition and targeting receptor tyrosine kinases.^43–48^

The aim of this study was to utilise the Biginelli reaction to integrate the 1,4-disubstituted 1,2,3-triazole and benzyloxyphenyl group using a wide variety of catalysts like Lewis acids, salts, or ionic liquids to generate phenyloxymethyl-1,2,3-triazole derivatives with potentially distinct anticancer attributes. Furthermore, we employed lanthanum triflate as a catalyst that acts as a Lewis acid resulting in excellent yield over a short reaction period. From the series of compounds synthesized, we identified one derivative to have potent anti-proliferative effects across multiple solid tumour cell lines and effectively impede the cell cycle with a reduction in cancer stem cell numbers in primary patient-derived cancer cells. The derivative also induced cytotoxicity in combination with FDA-approved clinical drugs thus suggesting a novel paradigm in our efforts to utilise the Biginelli reaction and green catalysts to generate anti-cancer compounds.

## 2 Results and discussion

### 2.1 Chemistry

**Scheme 1:**
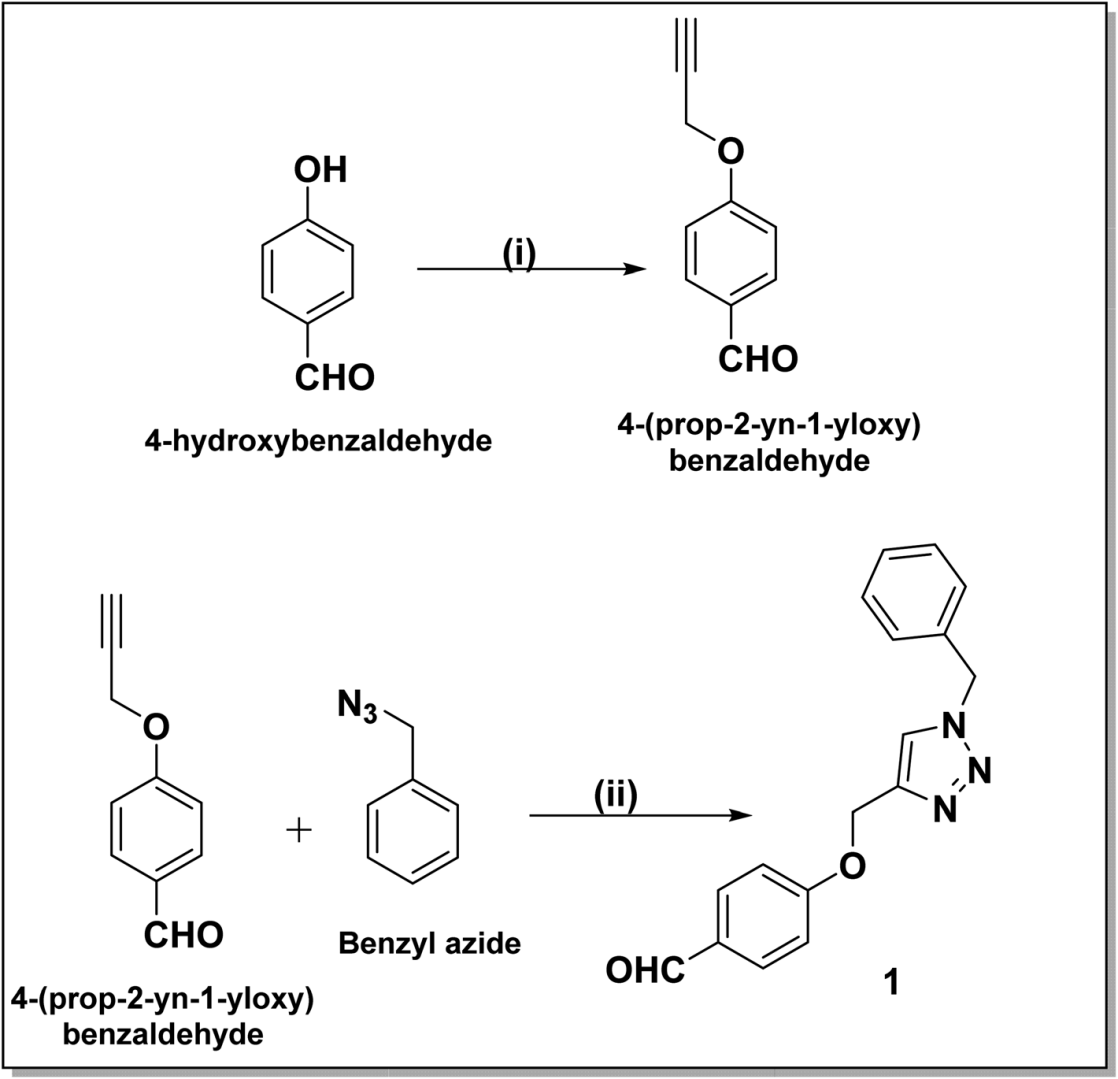
Synthesis of the 4-[(1-benzyl-1*H*-1,2,3-triazol-4-yl)methoxy]benzaldehyde (a) reaction conditions: (i) propargyl bromide, acetone, K_2_CO_3_, 2 h. (ii) CuSO_4_, sodium ascorbate, DMF/water (8 : 2), 3 h.

The synthesis of benzyl triazolyl methoxy derivative of DHPM was carried out by the multicomponent reaction of the 4-((1-benzyl-1*H*-1,2,3-triazole-4-yl)methoxy)benzaldehyde, different acetoacetanilide derivatives, and urea/thiourea. The inclusion of this aldehyde in the reaction is anticipated to enhance its biological applications. Compound 1 which is 4-((1-benzyl-1*H*-1,2,3-triazole-4-yl)methoxy)benzaldehyde, was prepared through a click reaction involving 4-(prop-2-yn-1-yloxy)benzaldehyde and benzyl azide, as illustrated in Scheme 1. The reaction was carried out by employing 10 mol% lanthanum triflate as a catalyst and under a heating environment at 100 °C for about 1–1.5 h, which resulted in good-to-excellent yields.

Our initial study was carried out both in solvent-free conditions as well as under various solvents like ethanol, acetonitrile, ethylene dichloride, tetrahydrofuran, dimethylformamide, and water (Table 1). The effect of solvent amount and required time was investigated. Among the mentioned solvents, ethanol gave the best outcomes with an excellent yield. It was found that using more solvent caused the reaction to take 3.5 hours to complete under reflux conditions. However, when the minimum amount of ethanol (0.5 ml) was utilized at 100 °C, the reaction mixture solidified within the 35 min but remained slightly incomplete. Hence, we decided to use 0.5 ml more ethanol to the reaction mixture and stir for 25 minutes. This successfully completed the reaction, and we observed a high-yield conversion of DHPM. Having set optimized parameters, La(OTf)_3_-catalyzed 20 derivatives of dihydopyrimidones were synthesized by employing various substrates (Table 2). All the synthesized compounds were characterized by ^1^H nuclear magnetic resonance (NMR), 13C{^1^H}^35^ attached proton test,^49^ and either high-resolution mass spectra (HRMS) or liquid chromatography-mass spectrometry (LCMS) analysis (see ESI†).

**Table 1:** Effect of solvent on the reaction^a^

Entry	Solvent	Temp.	Time	Conversion relative to aldehyde^b^
1	CH_3_CN^c^	Reflux	4 h	100%
2	DCE^c^	Reflux	4 h	Incomplete
3	THF^c^	Reflux	4 h	Incomplete
4	DMF^c^	Reflux	3 h	Mixture of product
5	Water^c^	Reflux	5 h	Incomplete
6	Ethanol^c^	Reflux	3.5 h	100%
7	Ethanol^d^	100 °C	1 h	100%
8	Solvent-free	100 °C	30 min	Incomplete
9	Catalyst-free	100 °C	30 min	Mixture of product

a Abbreviations: DCE, 1,2-dichloroethane; DMF, dimethylformamide; TLC, thin-layer chromatography; THF, tetrahydrofuran. Reaction condition: 1 mmol 4-((1-benzyl-1*H*-1,2,3-triazole-4-yl)methoxy)benzaldehyde 1c, 1.1 mmol acetoacetanilide derivative 2c, 1.2 mmol urea 3, and 0.1 mmol La(OTf)_3_.

b Observation from TLC analysis.

c More than 0.5 ml solvent.

d 0.5 ml solvent.

**Table 2:** Synthesis of benzyloxy DHPMs by the multicomponent reaction of 1 mmol 4-((1-benzyl-1*H*-1,2,3-triazole-4-yl)methoxy)benzaldehyde 1, 1.1 mmol of acetoacetenilide derivatives 2(a–k), 1.2 mmol urea derivative (3(a–b); X = O/S), and 0.1 mmol La(OTf)_3_

Entry	–R	X	Product	Time (h)	Yield^a^ (%)
1	–H	O	4a	1	97
2	2-Cl	O	4b	1	89
3	3-Cl	O	4c	1	90
4	4-Cl	O	4d	1	91
5	4-F	O	4e	1.5	88
6	3-CF_3_	O	4f	1.5	91
7	2-CH_3_	O	4g	1	87
8	4-CH_3_	O	4h	1	86
9	2-OCH_3_	O	4i	1	91
10	4-OCH_3_	O	4j	1	89
11	3,4-(CF_3_)_2_	O	4k	1.5	79
12	–H	S	4l	1	94
13	2-Cl	S	4m	1	92
14	3-Cl	S	4n	1	89
15	4-Cl	S	4o	1	95
16	4-F	S	4p	1.5	85
17	3-CF_3_	S	4q	1.5	86
18	2-CH_3_	S	4r	1	88
19	4-CH_3_	S	4s	1	90
20	2-OCH_3_	S	4t	1	88

a Isolated yield.

**Scheme 2:**
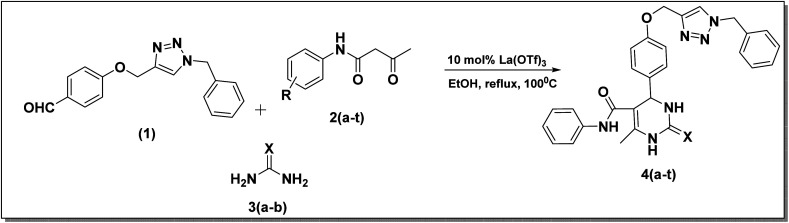
Multicomponent synthesis of dihydropyrimidinones from 4-((1-benzyl-1*H*-1,2,3-triazole-4-yl)methoxy)benzaldehyde 1, acetoacetenilide derivatives 2(a–t), and urea derivative 3(a–b).

After improving the reaction, the substrate scope for the La(OTf)_3_-catalyzed synthesis of dihydropymidine derivatives was investigated by changing the different acetoacetanilide derivatives. Substitution of the acetoacetanilides gave a slight variation in the time of reaction and yield of the product (Table 2). Compound 4a was obtained with an excellent 97% yield in one hour (Table 2, entry 1), while when 3,4-(CF_3_)_2_ substituted acetoacetanilide derivative was employed under the same reaction condition, a slightly decreased yield of 79% was observed (Table 2, entry 11) in 1.5 h. When chloro substituted acetoacetanilide derivatives were used as a substrate, a comparatively good yield was observed than the methyl-substituted acetoacetanilide derivatives (Table 2). In the case of trifluoro methyl substituted acetoacetanilide derivative, it takes 1.5 h to complete the reaction. Based on the above-discussed facts, it was observed that substitutions on the acetoacetanilide have shown great influence on the reaction time as well as in isolated yield (Scheme 2).

**Scheme 3:**
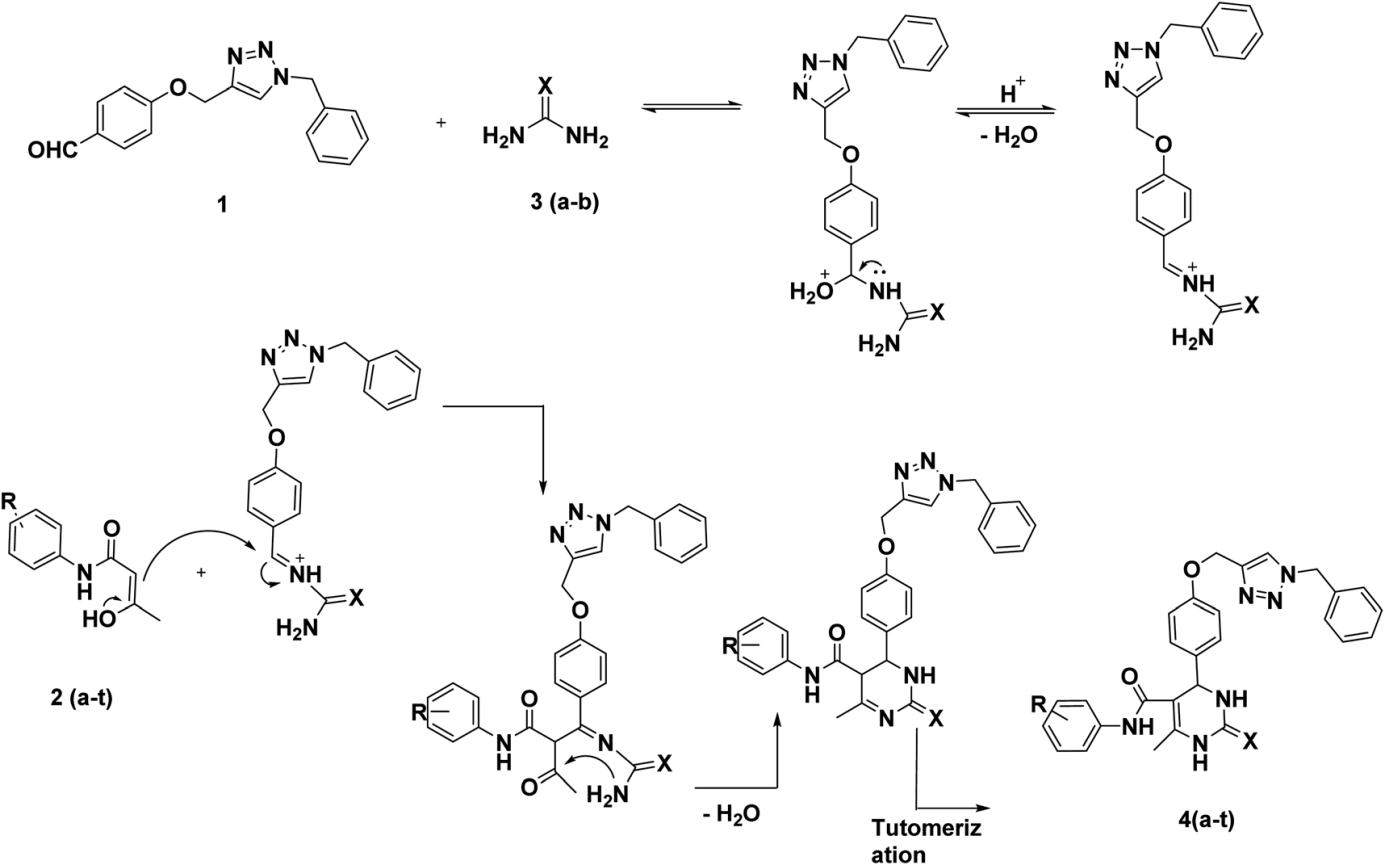
Plausible mechanism of La(OTf)_3_ catalyzed synthesis of DHPMs.

The Biginelli reaction is an example of an acid-catalysed three-component reaction. In the first step of the mechanism, the acid protonates the aldehyde. The nucleophilic NH_2_ group from urea then attacks the electrophilic aldehyde, leading to the formation of an *N*-acyliminium ion intermediate with the release of a water molecule. Next, the enol form of the β-keto amide attacks the *N*-acyliminium ion, resulting in the formation of a ureide intermediate. This intermediate subsequently converts into the final product (Scheme 3).

### 2.2 Biological evaluation

#### 2.2.1 Establishing a potential relationship between structure and biological activity of dihydropymidine derivatives

**Fig. 2:**
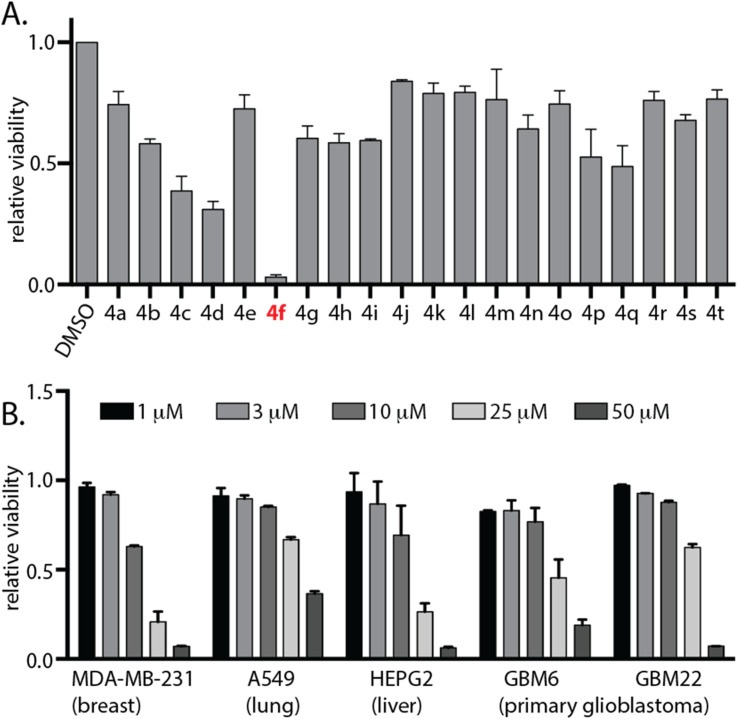
4f exhibits anti-cancer activity in diverse cancer cell lines. (A) Cell viability of a panel of DHPM derivatives 4a–t at 25 μM against human U87 cell line was assessed after 96 hours using the CellTiter 96 AQueous Non-Radioactive Cell Proliferation Assay kit. Viability of DMSO-treated cells was used as a control. Data are represented as fold viability of DMSO-treated control. (B) Indicated cell lines were treated with various doses of 4f for 72 hours and their viability was measured using CellTiter 96 AQueous Non-Radioactive Cell Proliferation Assay kit. Viability of DMSO-treated cells was used as control. Data are represented as fold viability of DMSO-treated control for each cell line with *n* = 3 biological replicates. See also ESI Fig. S1.†

To determine the biological relevance of these molecules *in vitro*, a cell viability assay was conducted on all synthesised compounds in triplicates at 25 μM against human cell line U87 MG. Out of the 20 molecules tested, 4f had the maximum potency (Fig. 2A). MDA-MB-231, A549, and HEPG2 cell lines, as well as primary patient derived glioblastoma cells GBM6 and GBM22, were used to assess the anti-cancer properties of 4f*in vitro* using cell viability assays. After 72 h incubation, cell viability was measured, and the EC_50_ values for 4f in MDA-MB-231 is 10.43 μM, A549 is 38.07 μM, HepG2 is 10.5 μM, GBM22 is 31.23 μM, and GBM6 is 22.82 μM (Fig. 2B). Since 4f treatment leads to reduced cell numbers over 48–96 hours, we wanted to query whether 4f function is cytotoxic or cytostatic. Hence, we carried out an assay to observe induction of apoptosis. We treated GBM6 cells with 25 μM of 4f and utilised FDA-approved drug crenolanib as a positive control. 10 μM of crenolanib induced significant apoptosis in GBM6 cells, however, 4f did not induce apoptotic cell death suggesting that 4f may be cytostatic in nature (ESI Fig. S1†).

#### 2.2.2 4f causes significant cell cycle delay in cancer cells

**Fig. 3:**
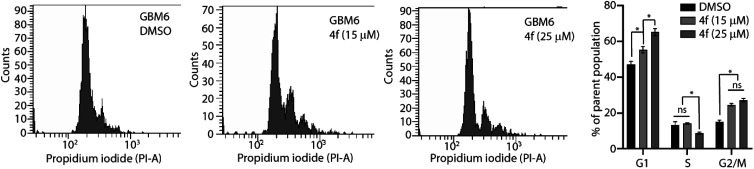
4f exhibits cell cycle defects in GBM6 cells. Asynchronous GBM6 cells were treated with various doses of 4f for 16 hours and the cell cycle distribution was analysed using propidium iodide and quantified using flow cytometry. * Indicates statistical significance compared to DMSO-treated control cell population; one-way ANOVA with Tukey's multiple comparison; ns: not significant.

Since 4f did not induce apoptosis in cancer cells, we enquired if the cytostatic function observed of 4f could be due to the induction of cell cycle defects. For this purpose, we utilised asynchronous GBM6 cells and treated then with or without 15 or 25 μM 4f over 16 hours. The cells were harvested post-treatment, fixed with ice-cold 70% ethanol, and the DNA content were labelled with propidium iodide. Following this, cell cycle stages of the cells were analysed using flow cytometry. Interestingly, 4f treatment led to a dose-dependent increase in G1 and G2/M population of cells with a significant decrease in S-phase cells (Fig. 3). This clearly suggests that the cytostatic function of 4f is indeed driven by induction of cell cycle delays in cancer cells.

#### 2.2.3 Combination with clinically relevant brain-penetrant drugs

**Fig. 4:**
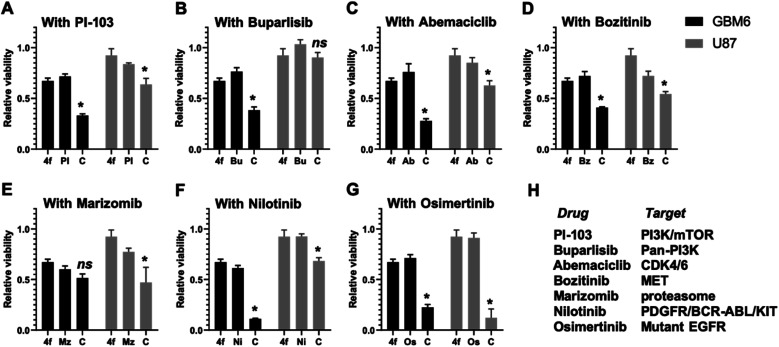
4f induces enhanced cytotoxicity in combination with specific clinically relevant brain-penetrant small molecule inhibitors. U87 and GBM6 cells were treated with 4f alone (10 and 25 μM respectively) or clinically relevant brain-penetrant drugs alone (A) PI-103 100 nM, (B) buparlisib 500 nM, (C) abemaciclib 1 μM, (D) bozitinib 7 μM, (E) marizomib 400 nM, (F) nilotinib 5 μM, (G) osimertinib 2 μM, or a combination C of both for 48 hours and cell viability was analysed by CellTiter 96 AQueous Non-Radioactive Cell Proliferation Assay kit. Viability of DMSO-treated cells was used as control. Data are represented as fold viability of DMSO-treated control for each cell line (* indicates statistical significance compared to each single compound treatment; one-way ANOVA with Dunnett's multiple comparison; ns: not significant). Same dataset for 4f was used across all combination studies from (A)–(G). (H) Table lists the cellular targets of the respective clinically relevant brain-penetrant drugs.

Since 4f induces cytostatic effects through cell cycle impediment, we wanted to test if a combination with clinical drugs could promote synergism in inducing cancer cell death. For this purpose, we tested 4f in two different cell lines (U87 MG and GBM6) in combination with PI-103, buparlisib, abemaciclib, bozitinib, marizomib, nilotinib, and osimertinib (Fig. 4A–G). Interestingly, 4f induced potent cytotoxicity in combination with nilotinib, and osimertinib (Fig. 4F and G) in the cells. While an additive combination effect was observed across all drugs with 4f, the synergistic effect seen in combination with EGFR inhibitor osimertinib was very robust and observed across both cell types.

#### 2.2.4 Establishing cytotoxicity in glioblastoma stem 3D neurospheres

**Fig. 5:**
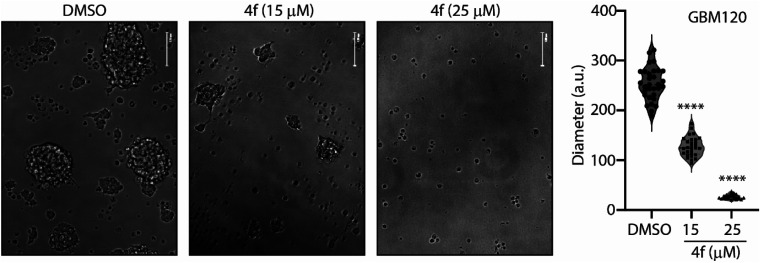
Compound 4f induces anti-glioma activity in 3D glioblastoma cell cultures. GBM120 cancer stem cells were treated with either DMSO or 15 μM or 25 μM or 4f for 13 days and neurospheres were allowed to form. A representative image of neurospheres is shown. Scale bar = 125 μm. The diameter of the neurospheres were quantified using ImageJ. The significance of the differences was measured using one-way ANOVA with Tukey's multiple comparisons for *n* = 3 biological replicates; ****p < 0.0001.

To further determine whether 4f treatment could impact a 3D glioma stem cell culture system, we treated GBM120 glioma stem cell lines with 15 and 25 μM 4f for 13 days. 4f treated GBM120 neurospheres were significantly smaller in size compared to DMSO control as observed under bright field (Fig. 5), and neurosphere formation was drastically reduced at the highest concentration (Fig. 5).

#### 2.2.5 *In silico* ADMET prediction

The most crucial stage in the process of discovering and developing new drugs involves anticipating the ADME (Absorption, Distribution, Metabolism, and Excretion) properties. *In silico* ADMET prediction of most bioactive compound 4f has been performed and their drug-likeness has been assessed through ADME prediction. The most potent compound and their prediction of drug-likeness are mentioned in Table 3. The physicochemical property value of 4f lies in the zone of various filters (Table 3). The molecule 4f shows good drug likeness properties as it follows Lipinski, veber, egan and muegge rule. There are two violations in terms of molecular weight and molar refractivity observed in the Ghose rule. This, 4f has drug-likeness with the bioavailability score of 0.55. This drug likeness data of 4f makes it a good drug candidate. Furthermore, other parameters like molecular weight (MW), topological surface area (TPSA), molar refractivity (MR), rotational bond (RB), hydrogen bond donor and acceptors lay in the acceptable range. Other than that, important physicochemical data is indicated in Table 3.

Predicted physicochemical properties of 4f with various filters for drug-likeness^a^

**Table:** 

Comp.	MW	RB	HBA	HBD	MR	TPSA	X log *P*	W log *P*	M log *P*	NR	NC	NH	Atom
4f	562.54	10	8	3	151.21	110.17	3.74	4.91	3.32	5	29	12	66

a Abbreviation: MW: molecular weight; RB: rotational bond; HBA: H-bond acceptor; HBD: H-bond donor; MR: molecular refractivity; TPSA: topological polar surface area; NR: no. of rings; NC: no. of carbon; NH: no. of heteroatoms.

**Table:** 

Lipinski filter	Ghose filter	Veber filter	Egan filter	Muegge filter
MW ≤ 500	160 ≤ MW ≤ 480	RB ≤ 10	W log *P* ≤ 5.88	200 ≤ MW ≤ 600
M log *P* ≤ 4.15	−0.4 ≤ W log *P* ≤ 5.6	TPSA ≤ 140	TPSA ≤ 131.6	−2X log *P* ≤ 5; TPSA ≤ 150
HBA ≤ 10	40 ≤ MR ≤ 130			NR ≤ 7; NC > 4; NH > 1; RB ≤ 15; HBA ≤ 10; HBD ≤ 5
HBD ≤ 5	20 ≤ atoms ≤ 70			

## 3 Conclusion

A series of benzyloxy derivatives of DHPM was synthesised using a multicomponent reaction. Lanthanum triflate catalyzed reaction gave good to excellent yield within 1–1.5 h. The advantages of this protocol include good to high yields, operational simplicity, simple filtration and needing no extraction or separation by column chromatography is necessary. Out of the series we identified 4f with the most potent anti-cancer activity in a diverse set of cancer cell lines including primary patient-derived cells. Furthermore, in combination with brain-penetrant small molecule kinase and proteasome inhibitors, 4f induced potent cytostatic activity and cell cycle impediment which attests for further medicinal chemistry and development of the benzyloxy derivatives of DHPM backbone. Interestingly, treating patient derived glioma stem 3D neurospheres with 4f significantly reduced neurosphere diameter (Fig. 5), this suggesting potent anti-glioma activity in stem-like organoid models. Furthermore, 4f exhibited a remarkable potency in combination with EGFR-mutant targeting osimertinib. Although kinase inhibitory activity (ESI Fig. S2†) or apoptosis (ESI Fig. S1†) were not observed for 4f, the data do suggest that a future *in vivo* bioavailable derivative of 4f could be used as a combination with osimertinib to target cancers with mutated/amplified EGFR. Both osimertinib and marizomib are covalent inhibitors and represent a paradigm shift toward cancer therapeutics. The ability of our drug to efficiently combine with covalent inhibitor provides an impetus toward further SAR and congener exploration to improve the DHPM backbone for clinical readiness. Compound 4f shows the most promising drug candidate due to its favorable drug-likeness and *in silico* ADMET properties. Therefore, this study establishes a novel synthetic scheme which will allow for the development of future clinically relevant anti-cancer molecules which can be used in combination with specific drugs targeting EGFR mutated cancers.

## 4 Experimental

### 4.1 General

All chemicals were purchased from commercially available sources and used without further purification. Melting points were determined by the open capillary tube method and are uncorrected. ^1^H NMR and ^13^C{1H} NMR, HSQC, and HMBC spectral analysis were recorded on BRUKER AVANCE II 600 NMR Spectrometer equipped with cryogenic TCI probe using DMSO-*d*_6_ as the solvents. Abbreviation used for NMR signal: s = Singlet, d = Doublet, t = triplet, dd = double doublet m = Multiplet. The chemical shifts are expressed in parts per million and coupling constants (*J*) are provided in Hertz.

### 4.2 General procedure for the synthesis of spiroxindoles 4(a–t)

A mixture of 1 mmol 4-((1-benzyl-1*H*-1,2,3-triazole-4-yl)methoxy)benzaldehyde 1(a–c), 1.1 mmol of acetoacetenilide derivatives 2(a–k), and 1.2 mmol urea derivative 3(a–b) was added to a 50 ml round bottom flask with 0.5 ml ethanol and 0.1 mmol (10 mol%) lanthanum triflate. It was stirred at 100 °C for 35 min. Within this time, the reaction mixture solidified or became sticky, and then 0.5 ml more ethanol was added and stirred for the required time, as mentioned in Table 2. Next, 5 ml more ethanol was added and cooled to room temperature. The progress of the reaction was monitored by TLC. After completion of the reaction, the reaction mixture was poured slowly into 20 ml ice-cold water with stirring and kept still until the precipitation of the product was completed. The crude product was filtered and washed with 20% aqueous solution of ethanol (5 ml × 3). Products were recrystallized from 6 ml ethanol.

#### 4.2.1 4-(4-((1-Benzyl-1*H*-1,2,3-triazol-4-yl)methoxy)phenyl)-6-methyl-2-oxo-*N*-phenyl-1,2,3,4-tetrahydropyrimidine-5-carboxamide (4a)

White solid (0.482 g, 97%), mp: 190–200 °C; ^1^H NMR (600 MHz, DMSO-d_6_) *δ*: 3.48 (s, 3H), 6.85 (d, *J* = 7.2 Hz, 2H), 6.88 (t, *J* = 7.8 Hz, 1H), 7.03 (d, *J* = 7.8 Hz, 1H), 7.18 (t, *J* = 7.8 Hz, 1H), 7.43 (NH_2,_ 2H), 7.44 (t, *J* = 7.8 Hz, 1H), 7.58 (d, *J* = 8.4 Hz, 1H), 7.751–7.739 (t, *J* = 8.4 Hz, 1H), 8.074 (d, *J* = 8.4 Hz, 1H), 10.53 (NH, 1H); ^13^C{^1^H} NMR (151 MHz, DMSO-d_6_) *δ*: 16.93 (CH_3_), 52.12 (–CH–), 54.35 (CH_2_), 60.96 (CH_2_), 105.47, 114.45, 119.47, 122.92, 124.48, 127.40, 128.37, 128.65, 135.87, 136.72, 138.20, 139.15, 142.93, 152.44 (C=O), 157.20 (C–O–), 165.23 (C=O); MS (ESI-TOF) *m*/*z* calcd for C_28_H_25_ClN_6_O_3_ (M + H)^+^: 494.21, found: 495.25.

#### 4.2.2 4-(4-((1-Benzyl-1*H*-1,2,3-triazol-4-yl)methoxy)phenyl)-*N*-(2-chlorophenyl)-6-methyl-2-oxo-1,2,3,4-tetrahydropyrimidine-5-carboxamide (4b)

White solid (0.469 g, 89%), mp: 118–120 °C; ^1^H NMR (600 MHz, DMSO-d_6_) *δ*: 2.12 (s, 3H), 5.07 (s, 2H), 5.28 (d, *J* = 2.8 Hz, 1H), 5.57 (s, 2H), 6.97 (d, *j* = 8.4 Hz, 2H), 7.10 (t, *j* = 1.6 Hz and 17.2 Hz, A–H), 7.39–7.20 (m, 10H), 7.45 (d, *J* = 1.6 Hz and 9.6 Hz), 7.57 (s, 1H), 8.26 (s, 1H), 8.78 (d, *j* = 2 Hz, 1H), 8.94 (s, 1H); ^13^C{^1^H} NMR (151 MHz, DMSO-d_6_) *δ*: 17.82 (CH_3_), 53.35 (–CH–), 54.83 (CH_2_), 61.57 (CH_2_), 104.60, 115.12, 125.23, 126.81, 127.19, 128.04, 128.38, 128.52, 128.71, 129.32, 129.87, 135.76, 136.57, 136.92, 141.13, 143.51, 15.92 (C=O), 157.97 (C–O–), 165.73 (C=O); MS (ESI-TOF) *m*/*z* calcd for C_28_H_25_ClN_6_O_3_ (M + H)^+^: 528.17, found: 529.03.

#### 4.2.3 4-(4-((1-Benzyl-1*H*-1,2,3-triazol-4-yl)methoxy)phenyl)-*N*-(3-chlorophenyl)-6-methyl-2-oxo-1,2,3,4-tetrahydropyrimidine-5-carboxamide (4c)

White solid (0.475 g, 90%), mp: 122–124 °C; ^1^H NMR (600 MHz, DMSO-d_6_) *δ*: 2.00 (s, 3H), 5.04 (s, 2H), 5.33 (dd, *j* = 2.8 Hz, 1H), 5.55 (s, 2H), 6.93 (d, *J* = 8.8 Hz, 2H), 7.01 (dd, *J* = 1.6 Hz and 9.6 Hz, 1H), 7.14 (d, *J* = 8.8 Hz, 2H), 7.21–7.35 (m, 7H), 7.43 (dd, *J* = 1.6 Hz and 9.6 Hz, 1H), 7.55 (t, *J* = 2.4 Hz, 1H), 7.73 (t, *J* = 2 Hz, 1H), 8.24 (s, 1H), 9.70 (s, 1H); ^13^C{^1^H} NMR (151 MHz, DMSO-d_6_) *δ*: 17.66 (CH_3_), 53.34 (–CH–), 54.81 (CH_2_), 61.54 (CH_2_), 105.62, 115.09, 118.34, 119.39, 123.19, 125.20, 128.03, 128.51, 128.70, 129.31, 130.76, 133.39, 136.56, 137.28, 139.87, 141.32, 143.52 (C=O), 157.85 (–C–O–), 166.13 (C=O); MS (ESI-TOF) *m*/*z* calcd for C_28_H_25_ClN_6_O_3_ (M + H)^+^: 528.16, found: 529.01.

#### 4.2.4 4-(4-((1-Benzyl-1*H*-1,2,3-triazol-4-yl)methoxy)phenyl)-*N*-(4-chlorophenyl)-6-methyl-2-oxo-1,2,3,4-tetrahydropyrimidine-5-carboxamide (4d)

White solid (0.480 g, 91%), mp: 128–130 °C; ^1^H NMR (600 MHz, DMSO-d_6_) *δ*: 1.99 (s, 3H), 5.04 (s, 2H), 5.31 (d, *J* = 2.8 Hz, 1H), 5.55 (s, 2H), 6.93, (d, *J* = 8 Hz, 2H), 7.14 (d, *J* = 8 Hz, 2H), 7.24–7.35 (m, 7H), 7.55 (d, *J* = 8 Hz, 3H), 8.23 (s, 1H), 8.72 (s, 1H), 9.62 (s, 1H); ^13^C{^1^H} NMR (151 MHz, DMSO-d_6_) *δ*: 17.61 (CH_3_), 53.35 (–CH–), 54.87 (CH_2_), 61.54 (CH_2_), 105.77, 115.08, 121.56, 125.20, 127.09, 128.00, 128.51, 128.70, 128.95, 129.32, 136.55, 137.30, 138.78, 139.49, 153.02 (C=O), 157.05 (C–O), 165.94 (C=O); MS (ESI-TOF) *m*/*z* calcd for C_28_H_25_ClN_6_O_3_ (M + H)^+^: 528.16, found: 529.00.

#### 4.2.5 4-(4-((1-Benzyl-1*H*-1,2,3-triazol-4-yl)methoxy)phenyl)-*N*-(4-fluorophenyl)-6-methyl-2-oxo-1,2,3,4-tetrahydropyrimidine-5-carboxamide (4e)

Light brown (0.451 g, 88%), mp: 134–136 °C; ^1^H NMR (600 MHz, DMSO-d_6_) *δ*: 2.00 (s, 3H), 5.05 (s, 2H), 5.31 (d, *J* = 2.8 Hz, 1H), 5.56 (s, 2H), 6.94, (d, *J* = 8 Hz, 2H), 7.05 (t, *J* = 8 Hz, 2H), 7.15 (t, *j* = 8 Hz, 2H), 7.26–7.35 (m, 5H), 7.53 (d, *J* = 8 Hz, 3H), 8.23 (s, 1H), 8.69 (s, 1H), 9.55 (s, 1H); ^13^C{^1^H} NMR (151 MHz, DMSO-d_6_) *δ*: 17.58 (CH_3_), 53.35 (–CH–), 54.92 (CH_2_), 61.54 (CH_2_), 105.89, 115.08, 115.49, 115.71, 121.78, 121.85, 125.29, 128.71, 128.03, 128.52, 128.71, 129.32, 136.16, 136.55, 137.32, 139.03, 143.54, 153.08, 157.25, 157.84 (C=O), 159.64 (C–O), 165.78 (C=O); MS (ESI-TOF) *m*/*z* calcd for C_28_H_25_FN_6_O_3_ (M + H)^+^: 512.19, found: 513.08.

#### 4.2.6 4-(4-((1-Benzyl-1*H*-1,2,3-triazol-4-yl)methoxy)phenyl)-6-methyl-2-oxo-*N*-(3-(trifluoromethyl)phenyl)-1,2,3,4-tetrahydropyrimidine-5-carboxamide (4f)

Light brown (0.511 g, 91%), mp: 230–232 °C; ^1^H NMR (600 MHz, DMSO-d_6_) *δ*: 2.04 (s, 3H), 5.05 (s, 2H), 5.35 (d, *J* = 2.8 Hz, 1H), 5.56 (s, 2H), 6.94, (d, *J* = 8 Hz, 2H), 7.16 (d, *J* = 8 Hz, 2H), 7.26–7.35 (m, 6H), 7.45 (t, *J* = 8 Hz, 1H), 7.60 (s, 1H), 7.75 (d, *J* = 8 Hz, 1H), 8.02 (s, 1H), 8.23 (s, 1H), 8.79 (s, 1H), 9.81 (s, 1H); ^13^C{^1^H} NMR (151 MHz, DMSO-d_6_) *δ*: 17.70 (CH_3_), 53.35 (–CH–), 54.76 (CH_2_), 61.54 (CH_2_), 105.45, 115.13, 115.98, 119.84, 123.49, 125.18, 128.03, 128.51, 128.6, 129.31, 130.31, 13.55, 137.27, 140.26, 143.53, 153.00 (C=O), 157.89 (C–O), 166.29 (C=O); MS (ESI-TOF) *m*/*z* calcd for C_29_H_25_F_3_N_6_O_3_ (M + H)^+^: 562.19, found: 563.01.

#### 4.2.7 4-(4-((1-Benzyl-1*H*-1,2,3-triazol-4-yl)methoxy)phenyl)-6-methyl-2-oxo-*N*-(*o*-tolyl)-1,2,3,4-tetrahydropyrimidine-5-carboxamide (4g)

White solid (0.442 g, 87%), mp: 200–202 °C; ^1^H NMR (600 MHz, DMSO-d_6_) *δ*: 2.04 (s, 3H), 5.05 (s, 2H), 5.35 (d, *J* = 2.8 Hz, 1H), 5.56 (s, 2H), 6.94, (d, *J* = 8 Hz, 2H), 7.16 (d, *J* = 8 Hz, 2H), 7.26–7.35 (m, 6H), 7.45 (t, *J* = 8 Hz, 1H), 8.02 (s, 1H), 8.79 (s, 1H), 9.81 (s, 1H); ^13^C{^1^H} NMR (151 MHz, DMSO-d_6_) *δ*: 17.65 (CH_3_), 18.34, (CH_3_), 53.36 (–CH–), 55.22 (CH_2_), 61.59 (CH_2_), 105.36, 115.04, 125.21, 125.84, 126.30, 128.39, 129.33, 130.9, 133.43, 136.56, 137.08, 139.05, 143.55, 153.93 (C=O), 157.92 (–CH–), 165.72 (C=O); MS (ESI-TOF) *m*/*z* calcd for C_29_H_28_N_6_O_3_ (M + H)^+^: 508.22, found: 509.10.

#### 4.2.8 4-(4-((1-Benzyl-1*H*-1,2,3-triazol-4-yl)methoxy)phenyl)-6-methyl-2-oxo-*N*-(*p*-tolyl)-1,2,3,4-tetrahydropyrimidine-5-carboxamide (4h)

White solid (0.438 g, 86%), mp: 208–210 °C; ^1^H NMR (600 MHz, DMSO-d_6_) *δ*: 1.99 (s, 3H), 2.18 (s, 3H), 5.05 (s, 2H), 5.31 (d, *J* = 3.2 Hz, 1H), 5.55 (s, 2H), 6.93, (d, *J* = 8 Hz, 2H),7.00 (d, *J* = 8 Hz, 2H), 7.16 (d, *J* = 8 Hz, 2H), 7.26–7.35 (m, 5H), 7.39 (d, *J* = 8 Hz, 1H), 7.50 (s, 1H), 8.23 (s, 1H), 8.65 (s, 1H), 9.41 (s, 1H); ^13^C{^1^H} NMR (151 MHz, DMSO-d_6_) *δ*: 17.55 (CH_3_), 20.96 (CH_3_), 53.35 (–CH–), 54.97 (CH_2_) 61.55 (CH_2_), 106.17, 115.05, 120.11, 125.19, 128.05, 128.51, 128.71, 129.32, 129.41, 132.48, 136.55, 137.29, 137.37, 138.60, 143.54, 153.13 (C=O), 157.82 (C–O), 165.68 (C=O); MS (ESI-TOF) *m*/*z* calcd for C_29_H_28_N_6_O_3_ (M + H)^+^: 508.22, found: 509.10.

#### 4.2.9 4-(4-((1-Benzyl-1*H*-1,2,3-triazol-4-yl)methoxy)phenyl)-*N*-(2-methoxyphenyl)-6-methyl-2-oxo-1,2,3,4-tetrahydropyrimidine-5-carboxamide (4i)

White solid (0.477 g, 91%), mp: 220–222 °C; ^1^H NMR (600 MHz, DMSO-d_6_) *δ*: 2.15 (s, 3H), 3.60 (s, 3H), 5.07 (d, *J* = 12 Hz, 2H), 5.15 (d, *J* = 12 Hz, 2H), 5.56 (s, 2H), 6.81 (t, *J* = 8 Hz, 2H),7.00–7.16 (m, 8H), 7.55 (s, 1H), 7.83 (d, *J* = 8 Hz, 1H), 8.11 (s, 1H), 8.26 (s, 1H), 8.84 (s, 1H); ^13^C{^1^H} NMR (151 MHz, DMSO-d_6_) *δ*: 17.82 (CH_3_), 53.37 (CH_3_), 54.93 (–CH–), 56.03 (CH_2_), 61.57 (CH_2_), 104.31, 111.36, 115.29, 120.76, 121.72, 124.51, 125.23, 127.97, 128.54, 128.72, 129.33, 136.54, 142.34, 143.53, 149.79 (C=O), 152.69 (C–O), 158.18 (C–O), 165.06 (C=O).; MS (ESI-TOF) *m*/*z* calcd for C_29_H_28_N_6_O_4_ (M + H)^+^: 524.22, found: 525.03.

#### 4.2.10 4-(4-((1-Benzyl-1*H*-1,2,3-triazol-4-yl)methoxy)phenyl)-*N*-(4-methoxyphenyl)-6-methyl-2-oxo-1,2,3,4-tetrahydropyrimidine-5-carboxamide (4j)

White solid (0.467 g, 89%), mp: 226–228 °C; ^1^H NMR (600 MHz, DMSO-d_6_) *δ*: 1.19 (s, 3H), 3.65 (s, 3H), 5.05 (s, 2H), 5.31 (s, 2H), 5.55 (s, 1H), 6.78, (d, *J* = 8 Hz, 2H), 6.94 (d, *J* = 8 Hz, 2H), 7.15 (d, *J* = 8 Hz, 2H), 7.26–7.35 (m, 6H), 7.41 (d, *J* = 8 Hz, 1H), 7.50 (s, 1H), 8.23 (s, 1H), 8.65 (s, 1H), 9.37 (s, 1H); ^13^C{^1^H} NMR (151 MHz, DMSO-d_6_) *δ*: 17.54 (CH_3_), 53.35 (CH_3_), 54.98 (–CH–), 55.67 (CH_2_), 67.55 (CH_2_), 106.18, 114.17, 118.05, 121.67, 125.19, 128.05, 128.51, 128.71, 129.32, 132.92, 136.55, 137.38, 138.35, 143.57, 153.16 (C=O), 155.67 (C–O), 157.82 (C–O), 168.47 (C=O); MS (ESI-TOF) *m*/*z* calcd for C_29_H_28_N_6_O_4_ (M + H)^+^: 524.22, found: 525.08.

#### 4.2.11 4-(4-((1-Benzyl-1*H*-1,2,3-triazol-4-yl)methoxy)phenyl)-6-methyl-*N*-phenyl-2-thioxo-1,2,3,4-tetrahydropyrimidine-5-carboxamide (4k)

White solid (0.403 g, 79%), mp: 180–182 °C; ^1^H NMR (600 MHz, DMSO-d_6_) *δ*: 2.03 (s, 3H), 5.06 (s, 2H), 5.32 (d, *J* = 3.2 Hz, 1H), 5.56 (s, 2H), 6.98, (td, *J* = 2 Hz and 17.2 Hz, 3H), 7.14 (d, *J* = 8 Hz, 2H), 7.26–7.35 (m, 7H), 7.51 (d, *J* = 8 Hz, 2H), 8.23 (s, 1H), 9.38 (s, 1H), 9.68 (s, 1H), 9.95 (s, 1H); ^13^C{^1^H} NMR (151 MHz, DMSO-d_6_) *δ*: 17.03 (CH_3_), 53.36 (–CH–), 55.01 (CH_2_), 61.58 (CH_2_), 107.89, 115.22, 120.16, 123.86, 125.21, 128.24, 128.51, 128.71, 129.12, 129.32, 135.94, 136.08, 136.55, 139.54, 143.49, 158.11 (C=O), 165.53 (C–O), 174.38 (C=S); MS (ESI-TOF) *m*/*z* calcd for C_28_H_26_N_6_O_2_S (M + H)^+^: 445.1067, found: 445.1081.

#### 4.2.12 4-(4-((1-Benzyl-1*H*-1,2,3-triazol-4-yl)methoxy)phenyl)-*N*-(2-chlorophenyl)-6-methyl-2-thioxo-1,2,3,4-tetrahydropyrimidine-5-carboxamide (4l)

White solid (0.479 g, 94%), mp: 116–118 °C; ^1^H NMR (600 MHz, DMSO-d_6_) *δ*: 1.08 (t, *J* = 6.6 Hz, 3H), 4.13 (q, *J* = 3.72 Hz, 2H), 4.30 (dd, *J* = 2.64 Hz and 16.56 Hz, 1H), 4.46 (dd, *J* = 2.46 Hz and 15.51 Hz, 1H), 5.21 (d, *J* = 10.5 Hz, 1H), 5.49 (d, *J* = 17.28 Hz, 1H), 5.87 (tt, *J* = 4.14 Hz and 12 Hz, 1H), 6.95 (d, *J* = 8.4 Hz, 1H), 7.32 (t, *J* = 8.4 Hz, 1H), 7.45 (t, *J* = 7.2 Hz, 1H), 7.61 (NH_2,_ 2H), 7.65 (d, *J* = 8.4 Hz, 1H), 7.77 (t, *J* = 7.8 Hz, 1H), 8.10 (d, *J* = 8.4 Hz, 1H); ^13^C{^1^H} NMR (151 MHz, DMSO-d_6_) *δ*: 16.57 (CH_3_), 52.74 (–CH–), 54.34 (CH_2_), 61.00 (CH_2_), 105.99, 114.60, 124.52, 126.51, 126.79, 127.20, 127.75, 127.85, 128.04, 128.66, 129.29, 134.80, 135.16, 135.87, 136.91, 142.85, 157.56 (C=O), 164.87 (C–O), 173.68 (C=S); MS (ESI-TOF) *m*/*z* calcd for C_28_H_25_ClN_6_O_2_S (M + H)^+^: 510.18, found: 511.06.

#### 4.2.13 4-(4-((1-Benzyl-1*H*-1,2,3-triazol-4-yl)methoxy)phenyl)-*N*-(3-chlorophenyl)-6-methyl-2-thioxo-1,2,3,4-tetrahydropyrimidine-5-carboxamide (4m)

Light brown(0.500 g, 92%), mp: 120–122 °C; ^1^H NMR (600 MHz, DMSO-d_6_) *δ*: 2.04 (s, 3H), 5.06 (s, 2H), 5.32 (d, *J* = 3.2 Hz, 1H), 5.56 (s, 2H), 6.98, (d, *J* = 8 Hz, 2H), 7.04 (d, *J* = 8 Hz, 1H), 7.14 (d, *J* = 8 Hz, 2H), 7.26–7.35 (m, 6H), 7.42 (d, *J* = 8 Hz, 2H), 7.72 (s, 1H), 8.23 (s, 1H), 9.44 (s, 1H), 9.84 (s, 1H), 10.00 (s, 1H); ^13^C{^1^H} NMR (151 MHz, DMSO-d_6_) *δ*: 17.09 (CH_3_), 53.36 (–CH–), 54.89 (CH_2_), 61.57 (CH_2_), 107.41, 115.26, 118.43, 119.60, 123.51, 125.21, 128.21, 128.51, 128.71, 129.32, 130.86, 133.47, 136.03, 136.55, 136.82, 141.01, 143.47, 158.14(C=O), 165.79 (C–O), 174.42 (C=S); MS (ESI-TOF) *m*/*z* calcd for C_28_H_25_ClN_6_O_2_S (M + H)^+^: 544.14, found: 544.98.

#### 4.2.14 4-(4-((1-Benzyl-1*H*-1,2,3-triazol-4-yl)methoxy)phenyl)-*N*-(4-chlorophenyl)-6-methyl-2-thioxo-1,2,3,4-tetrahydropyrimidine-5-carboxamide (4n)

Light brown (0.484 g, 89%), mp: 126–128 °C; ^1^H NMR (600 MHz, DMSO-d_6_) *δ*: 2.04 (s, 3H), 5.06 (s, 2H), 5.32 (d, *J* = 3.2 Hz, 1H), 5.56 (s, 2H), 6.96 (d, *J* = 8 Hz, 2H), 7.14 (d, *J* = 8 Hz, 2H), 7.26–7.35 (m, 7H), 7.55 (d, *J* = 8 Hz, 2H), 8.23 (s, 2H), 9.44 (s, 1H), 9.84 (s, 1H), 9.98 (s, 1H); ^13^C{^1^H} NMR (151 MHz, DMSO-d_6_) *δ*: 17.07(CH_3_), 53.36 (–CH–), 54.94 (CH_2_), 61.57 (CH_2_), 107.57, 115.24, 121.66, 125.21, 127.40, 128.21, 128.56, 129.25, 129.32, 136.04, 136.47, 136.59, 138.49, 143.47, 158.12 (C=O), 165.61 (C–O), 174.41 (C=S); MS (ESI-TOF) *m*/*z* calcd for C_28_H_25_ClN_6_O_2_S (M + H)^+^: 544.14, found: 544.97.

#### 4.2.15 4-(4-((1-Benzyl-1*H*-1,2,3-triazol-4-yl)methoxy)phenyl)-*N*-(4-fluorophenyl)-6-methyl-2-thioxo-1,2,3,4-tetrahydropyrimidine-5-carboxamide (4o)

White solid (0.502 g, 95%), mp: 232–234 °C; ^1^H NMR (600 MHz, DMSO-d_6_) *δ*: 2.04 (s, 3H), 5.06 (s, 2H), 5.32 (d, *J* = 3.2 Hz, 1H), 5.56 (s, 2H), 6.96 (d, *J* = 8 Hz, 2H), 7.04 (d, *J* = 8 Hz, 1H), 7.14 (d, *J* = 8 Hz, 2H), 7.26–7.35 (m, 6H), 7.55 (t, *J* = 8 Hz, 2H), 8.23 (s, 1H), 9.40 (t, *J* = 2 Hz, 1H), 9.84 (s, 1H), 9.98 (s, 1H); ^13^C{^1^H} NMR (151 MHz, DMSO-d_6_) *δ*: 17.04 (CH_3_), 53.36 (–CH–), 54.98 (CH_2_), 61.57 (CH_2_), 107.69, 115.23, 115.58, 118.79, 121.89, 121.97, 125.21, 128.22, 128.51, 128.71, 129.32, 135.89, 136.07, 136.57, 143.48, 157.40, 158.12, 159.78(C=O), 165.44 (C–O), 174.40 (C=S); MS (ESI-TOF) *m*/*z* calcd for C_28_H_25_FN_6_O_2_S (M + H)^+^: 528.17, found: 529.02.

#### 4.2.16 4-(4-((1-Benzyl-1*H*-1,2,3-triazol-4-yl)methoxy)phenyl)-6-methyl-2-thioxo-*N*-(3-(trifluoromethyl)phenyl)-1,2,3,4-tetrahydropyrimidine-5-carboxamide (4p)

White solid (0.491 g, 85%), mp: 250–252 °C; ^1^H NMR (600 MHz, DMSO-d_6_) *δ*: 2.04 (s, 3H), 5.06 (s, 2H), 5.32 (d, *J* = 3.2 Hz, 1H), 5.56 (s, 2H), 6.96 (d, *J* = 8 Hz, 2H), 7.14 (d, *J* = 8 Hz, 2H), 7.26–7.35 (m, 6H), 7.47 (t, *J* = 8 Hz, 1H), 7.75 (t, *J* = 8 Hz, 2H), 8.02 (s, 1H), 8.23 (s, 1H), 9.40 (t, *J* = 2 Hz, 1H), 9.98 (s, 1H), 10.00 (s, 1H); ^13^C{^1^H} NMR (151 MHz, DMSO-d_6_) *δ*: 17.40 (CH_3_), 53.35 (–CH–), 54.82 (CH_2_), 61.57 (CH_2_), 107.20, 115.27, 116.08, 120.13, 123.60, 125.21, 128.23, 128.51, 128.70, 129.31, 130.39, 136.01, 136.55, 137.21, 140.34, 143.47, 158.16 (C=O), 165.97 (C–O), 174.43 (C=S); MS (ESI-TOF) *m*/*z* calcd for C_29_H_25_F_3_N_6_O_2_S (M + H)^+^: 578.17, found: 579.01.

#### 4.2.17 4-(4-((1-Benzyl-1*H*-1,2,3-triazol-4-yl)methoxy)phenyl)-6-methyl-2-thioxo-*N*-(*o*-tolyl)-1,2,3,4-tetrahydropyrimidine-5-carboxamide (4q)

White solid (0.451 g, 86%), mp: 190–192 °C; ^1^H NMR (600 MHz, DMSO-d_6_) *δ*: 1.87 (s, 3H), 2.04 (s, 3H) 5.09 (s, 2H), 5.32 (d, *J* = 3.2 Hz, 1H), 5.56 (s, 2H), 6.96–7.14 (m, 8H), 7.19 (t, *J* = 8 Hz, 2H), 7.47 (t, *J* = 8 Hz, 1H), 7.75 (t, *J* = 8 Hz, 2H), 8.02 (s, 1H), 8.23 (s, 1H), 9.40 (t, *J* = 2 Hz, 1H), 9.98 (s, 1H), 10.00 (s, 1H); ^13^C{^1^H} NMR (151 MHz, DMSO-d_6_) *δ*: 17.10 (CH_3_), 18.36 (CH_3_), 53.38 (–CH–), 55.29 (CH_2_), 61.61 (CH_2_), 107.36, 115.18, 125.28, 126.09, 126.39, 128.53, 128.57, 128.73, 129.34, 130.77, 133.54, 135.91, 136.02, 136.55, 136.74, 143.49, 158.18 (C=O), 165.46 (C–O), 174.55 (C=S); MS (ESI-TOF) *m*/*z* calcd for C_29_H_28_N_6_O_2_S (M + H)^+^: 524.20, found: 525.03.

#### 4.2.18 4-(4-((1-Benzyl-1*H*-1,2,3-triazol-4-yl)methoxy)phenyl)-6-methyl-2-thioxo-*N*-(*p*-tolyl)-1,2,3,4-tetrahydropyrimidine-5-carboxamide (4r)

White solid (0.461 g, 88%), mp: 184–186 °C; ^1^H NMR (600 MHz, DMSO-d_6_) *δ*: 2.02 (s, 3H), 2.18 (s, 3H) 5.09 (s, 2H), 5.30 (s, 1H), 5.56 (s, 2H), 6.96 (d, *J* = 8 Hz, 2H), 7.02 (d, *J* = 8 Hz, 2H), 7.13 (d, *J* = 8 Hz, 2H), 7.26–7.35 (m, 6H), 7.39 (d, *J* = 8 Hz, 1H), 8.23 (s, 1H), 9.36 (s, 1H), 9.59 (s, 1H), 9.98 (s, 1H); ^13^C{^1^H} NMR (151 MHz, DMSO-d_6_) *δ*: 17.60 (CH_3_), 20.97 (CH_3_), 53.36 (–CH–), 55.03 (CH_2_), 61.57 (CH_2_), 107.96, 115.20, 120.19, 125.21, 128.24, 128.51, 128.71, 129.32, 129.48, 132.80, 135.71, 136.10, 136.55, 137.02, 143.49, 158.09 (C=O), 165.31 (C–O), 174.36 (C=S); MS (ESI-TOF) *m*/*z* calcd for C_29_H_28_N_6_O_2_S (M + H)^+^: 524.19, found: 525.03.

#### 4.2.19 4-(4-((1-Benzyl-1*H*-1,2,3-triazol-4-yl)methoxy)phenyl)-*N*-(4-methoxyphenyl)-6-methyl-2-thioxo-1,2,3,4-tetrahydropyrimidine-5-carboxamide (4s)

White solid (0.487 g, 90%), mp: 200–202 °C; ^1^H NMR (600 MHz, DMSO-d_6_) *δ*: 2.16 (s, 3H), 3.63 (s, 3H), 5.09 (s, 2H), 5.22 (d, *J* = 8 Hz, 2H), 5.56 (s, 2H), 6.96 (dt, *J* = 1.6 Hz and 16.8 Hz, 1H), 6.98–7.02 (m, 3H), 7.31–7.35 (m, 2H), 7.74 (dd, *J* = 1.6 Hz and 9.6 Hz, 1H), 8.23 (s, 1H), 8.92 (s, 1H), 9.41 (d, *J* = 1.6 Hz, 1H), 10.00 (d, *J* = 1.6 Hz, 1H); ^13^C{^1^H} NMR (151 MHz, DMSO-d_6_) *δ*: 17.22 (CH_3_), 53.37 (CH_3_), 54.94 (–CH–), 56.08 (CH_2_), 61.60 (CH_2_), 106.42, 111.54, 115.34, 120.74, 122.55, 125.09, 125.23, 127.56, 128.53, 128.72, 129.33, 135.54, 136.54, 138.45, 143.48, 150.36 (C=O), 158.36 (C–O), 164.83 (C–O), 174.02 (C=S); MS (ESI-TOF) *m*/*z* calcd for C_29_H_28_N_6_O_3_S (M + H)^+^: 540.19, found: 541.00.

#### 4.2.20 4-(4-((1-Benzyl-1*H*-1,2,3-triazol-4-yl)methoxy)phenyl)-*N*-(2-methoxyphenyl)-6-methyl-2-thioxo-1,2,3,4-tetrahydropyrimidine-5-carboxamide (4t)

White solid (0.475 g, 88%), mp: 204–206 °C; ^1^H NMR (600 MHz, DMSO-d_6_) *δ*: 2.04 (s, 3H), 3.65 (s, 3H), 5.06 (s, 2H), 5.32 (d, *J* = 3.2 Hz, 1H), 5.56 (s, 2H), 6.80 (d, *J* = 8 Hz, 2H), 6.96 (d, *J* = 8 Hz, 2H), 7.14 (d, *J* = 8 Hz, 2H), 7.26–7.35 (m, 5H), 7.47 (d, *J* = 8 Hz, 1H), 8.23 (s, 1H), 9.40 (t, *J* = 2 Hz, 1H), 9.55 (s, 1H), 9.92 (s, 1H); ^13^C{^1^H} NMR (151 MHz, DMSO-d_6_) *δ*: 16.99 (CH_3_), 53.36 (CH_3_), 55.04 (–CH–), 55.68 (CH_2_), 61.58 (CH_2_), 107.99, 114.24, 115.20, 121.75, 125.20, 128.24, 128.51, 128.71, 129.33, 132.63, 135.50, 136.11, 136.55, 143.49, 155.84 (C=O), 158.09 (C–O), 165.08 (C–O), 174.34 (C=S); MS (ESI-TOF) *m*/*z* calcd for C_29_H_28_N_6_O_3_S (M + H)^+^: 540.19, found: 541.04.

### 4.3 Materials and methods

#### 4.3.1 Materials and cell lines

Drugs PI-103 (#A2067-APE), buparlisib (#ORB669009-BOR), abemaciclib (#S5716-SEL), bozitinib (#S6762-SEL), marizomib (#SML1916-100UG), nilotinib (#S1033-SEL), crenolanib (#S2730-SEL), and osimertinib (#S7297-SEsssL) were purchased from Stratech, UK as stated previously.^50^ The compounds were dissolved in DMSO to a working stock of 10 mM. Propidium iodide was purchased from Sigma-Millipore. MDA-MB-231 (a triple negative breast cancer epithelial cell line), A549 (a non-small cell lung cancer cell line), HEPG2 (derived from a patient with the hepatocellular carcinoma) and U87 MG (U87) cells were from ATCC. GBM6, GBM120, and GBM22 were acquired from the Brain Tumour PDX National Resource, Mayo Clinic, USA.^20^ Insulin and epidermal growth factor were purchased from Sigma Millipore.

#### 4.3.2 Cell culture

Mammalian cells were all grown in a humidified incubator with 5% CO_2_ at 37 °C. U87 and HEPG2 cell lines were cultured in Dulbecco's modified Eagle's medium (DMEM, Gibco) supplemented with 10% FBS and 1% penicillin and streptomycin. MDA-MB-231, A549 and primary patient glioblastoma cell lines (GBM6 and GBM22) were cultured in DMEM supplemented with 10% FBS, 1% penicillin and streptomycin, 10 μg ml^−1^ insulin, and 20 ng ml^−1^ hEGF as stated previously.^20^ GBM120 cells were cultured in neurosphere media consisting of KnockOut DMEM/F-12 Basal Media supplemented with StemPro NSC SFM Supplement, 10 mg FGF, 10 mg EGF, l-glutamine (Corning #25005CI) 10 ml of 200 mM solution, and 1% penicillin and streptomycin.

#### 4.3.3 Cell viability and apoptosis assays

To measure cell viability, actively proliferating cells were seeded at with an equal number of cells per well. Cell viability assays were carried out with 48–96 h treatment of indicated drugs or DMSO control using CellTiter 96® AQueous Non-Radioactive Cell proliferation assay, adhering to manufacturer instructions. Absorbance was measured using a Tecan multi-well plate reader and data was represented as % viability compared to DMSO treated control as stated previously.^20,51,52^ For the assessment of apoptosis, Annexin V-FITC (ab176749; Abcam, Cambridge, UK) was used as stated previously.^53^ 16 h post drug treatments, the wells were washed and buffer solution containing Annexin V-FITC diluted as par manufacturer's instructions were added to the wells. The cells were incubated for 90 min prior to imaging. Bright field and fluorescent imaging were taken using a Thermo Scientific EVOS imaging system.

#### 4.3.4 Cell cycle analyses

Cell cycle analyses using propidium iodide and flow cytometry were carried out as described previously.^52,54^ Asynchronous GBM6 cells were treated with either DMSO or 4f at 15 or 25 μM for 16 h. Post treatment, cells were washed with PBS + 1% FBS and resuspended in flow cytometry tubes. Cells were then fixed with 70% ice-cold ethanol and propidium iodide (50 μg ml^−1^) was added to the cells and incubated in the dark at room temperature (25 °C) for 30 min. The cell populations were then subjected to quantitative measurement of DNA content by flow cytometry using a FACSFortessa (BD Biosciences) and cell cycle distribution and the percentage of G_2_/M–S–G_1_ cells to the total cell events were determined by the BD FACS Diva software. Stacked bar graphs were derived using Graphpad Prism.

#### 4.3.5 Neurosphere formation assay

Neurosphere formation assay was carried out as stated previously.^20^ Briefly, GBM120 cells were plated at 4000 cells per well in neurosphere media supplemented with either DMSO, 15 μM and 25 μM of 4f for 13 days in triplicates. After 13 days, representative images were taken of each well using the Zeiss Axiovert Live microscope. Diameters of the neurospheres were quantified using ImageJ software, and graphs were plotted on GraphPad Prism.

#### 4.3.6 Kinase screen analysis of 4f

Kinase inhibitor specificity profiling assays were carried out at The International Centre for Protein Kinase Profiling (http://www.kinase-screen.mrc.ac.uk/) as stated previously.^20^ Briefly, 4f biochemical kinase inhibitory property was determined against a panel of 139 protein kinases as described previously.^55,56^ Protein kinases were assayed *in vitro* with 10 μM final concentration of 4f and results are presented as a percentage of kinase activity to DMSO control reactions as an average of triplicate reactions in the form of comparative histograms using Adobe Illustrator.

#### 4.3.7 Statistical analysis

All analysis was conducted using Graphpad Prism statistical package and presented as mean ± SD unless otherwise stated. Figure legends contain details of the statistical tests and multiple comparisons conducted throughout. Experiments were repeated 2–3 times with multiple technical replicates for the appropriate statistical tests to be conducted.

#### 4.3.8 *In silico* ADMET prediction

*In silico* ADMET prediction of the synthesized DHPM compound 4f was accomplished with the help of the web tool SwissADME^57^ (http://www.swissadme.ch/).

## Data availability

The data supporting this article have been included as part of the ESI.†

## Conflicts of interest

DBU, JN, RMV, SB, and HMP are named inventors on the patent no. 528662 awarded by the government of India on 18th March 2024 pertaining to these reported compounds. No other conflicts of interest reported.

## Supplementary Material

RA-014-D4RA04171B-s001
