# Supplementary material for: One-pot synthesis of tetrahydropyrimidinecarboxamides enabling in vitro anticancer activity: a combinative study with clinically relevant brain-penetrant drugs
Source: RSC Adv. 2024 Aug 27;14(37):27174–86. doi: 10.1039/d4ra04171b (PMC11348845; doi:10.1039/d4ra04171b)
Supplement: RA-014-D4RA04171B-s001 [file RA-014-D4RA04171B-s001.pdf]

Supplementary Information (SI) for RSC Advances.
This journal is © The Royal Society of Chemistry 2024

One-pot synthesis of tetrahydropyrimidinecarboxamides enabling In Vitro anticancer activities: A combinative study with clinically relevant brain-penetrant drugs

Dipti B. Upadhyay[a,#], Joaquina Nogales[b,#], Jaydeep A. Mokariya[a], Ruturajsinh M. Vala[a], Vasudha Tandon[b], Sourav Banerjee [b,*], Hitendra M. Patel [a,*]

[a] Department of Chemistry, Sardar Patel University, Vallabh Vidyanagar, Gujarat, India

[b] Department of Cellular & Systems Medicine, School of Medicine, University of Dundee, Dundee DD1 9SY, United Kingdom

[#] contributed equally

* Corresponding authors: Hitendra M. Patel, Email: hm_patel@spuvvn.edu

Sourav Banerjee, Email: s.y.banerjee@dundee.ac.uk

Table of Contents

Sr. No.	Content	Page No.
1	**4f** does not induce apoptosis in cancer cells	2
2	kinase inhibitory activity Figure	2
3	^{1}H-NMR and ^{13}C{^{1}H}-NMR spectra of compound **4a-4t**	3-23
4	LCMS of compound **4a-4t**	23-28

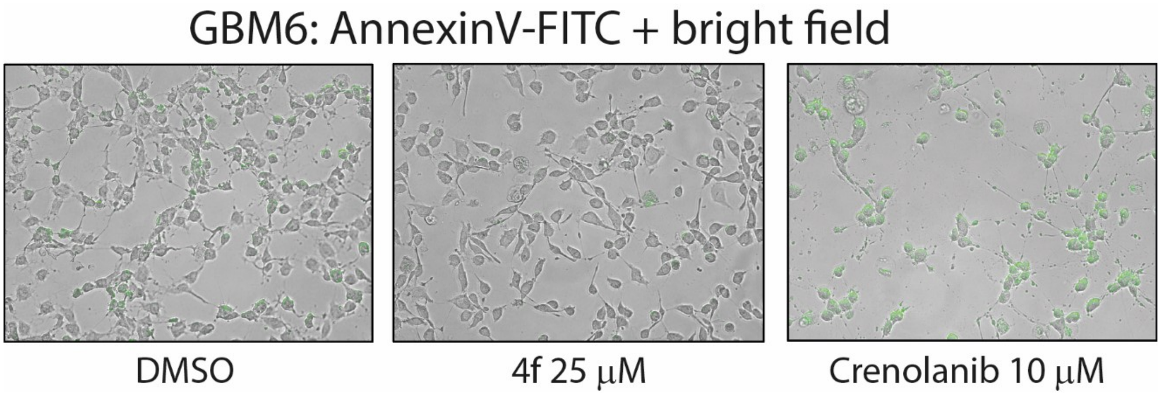

Figure S1:4f at 25 μM does not induce apoptosis in GBM6 after 16 hr treatment. Images are superimposed bright field with FITC fluorescent channels. Crenolanib treatment is used as control for apoptosis induction.

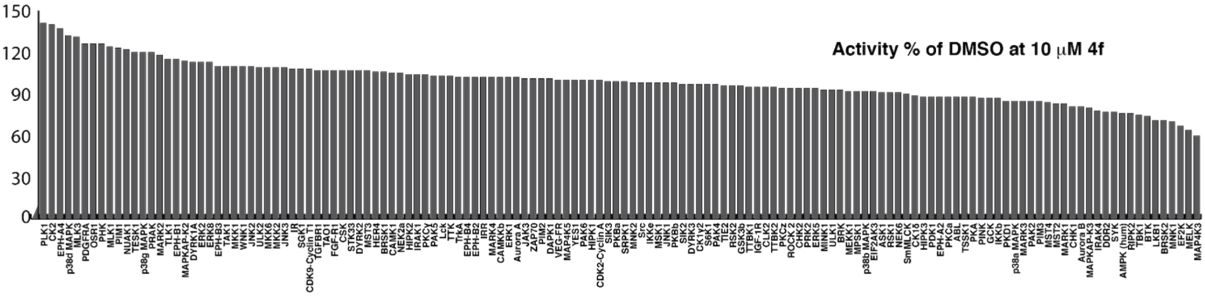

Figure S2: Kinase profiling of **4f** at 10 μM was carried out against the panel of 139 kinases at the International Centre for Protein Kinase Profiling (http://www.kinase-screen.mrc.ac.uk/).

Copies of **^{1}H NMR** and **^{13}C{^{1}H}- NMR** spectra for compound **4**a-**4**ab

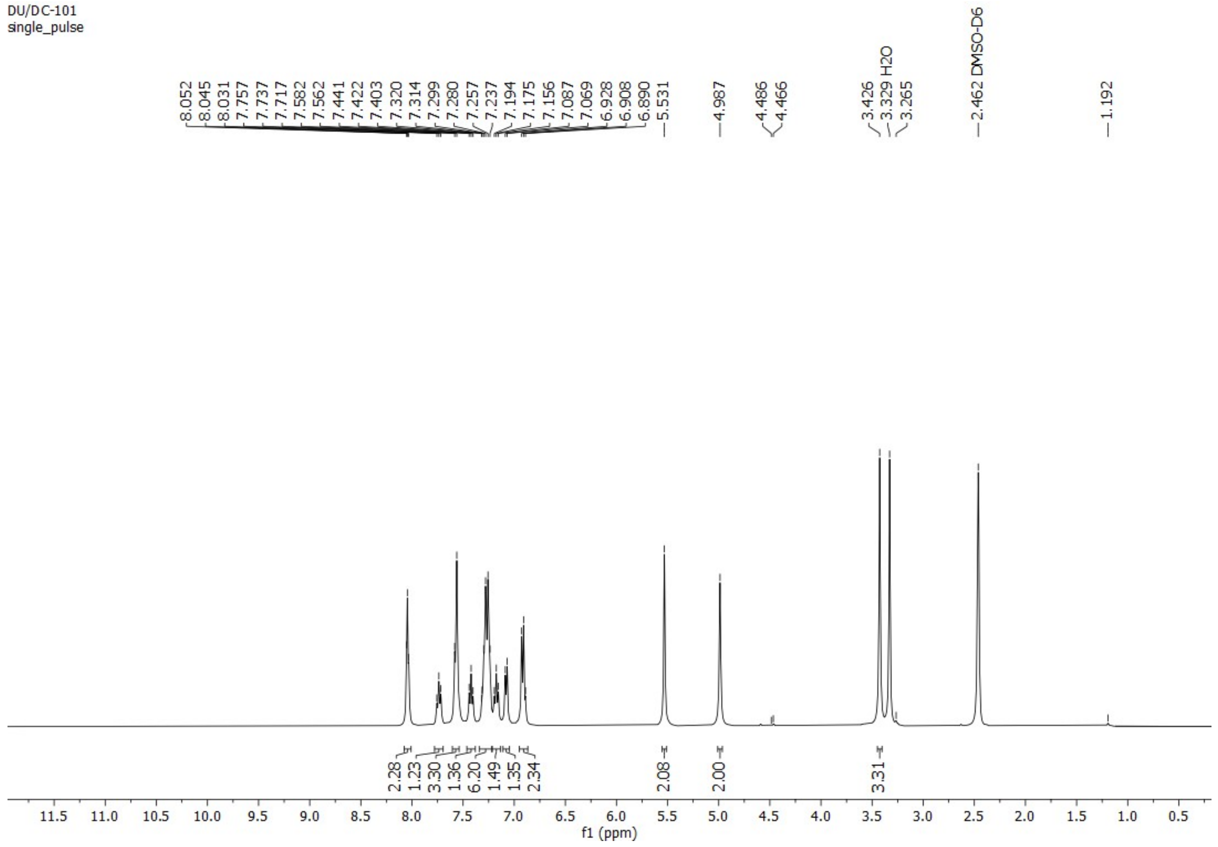

Figure S3. ^{1}H NMR spectrum of compound **4a** at 400 MHz in DMSO-d6

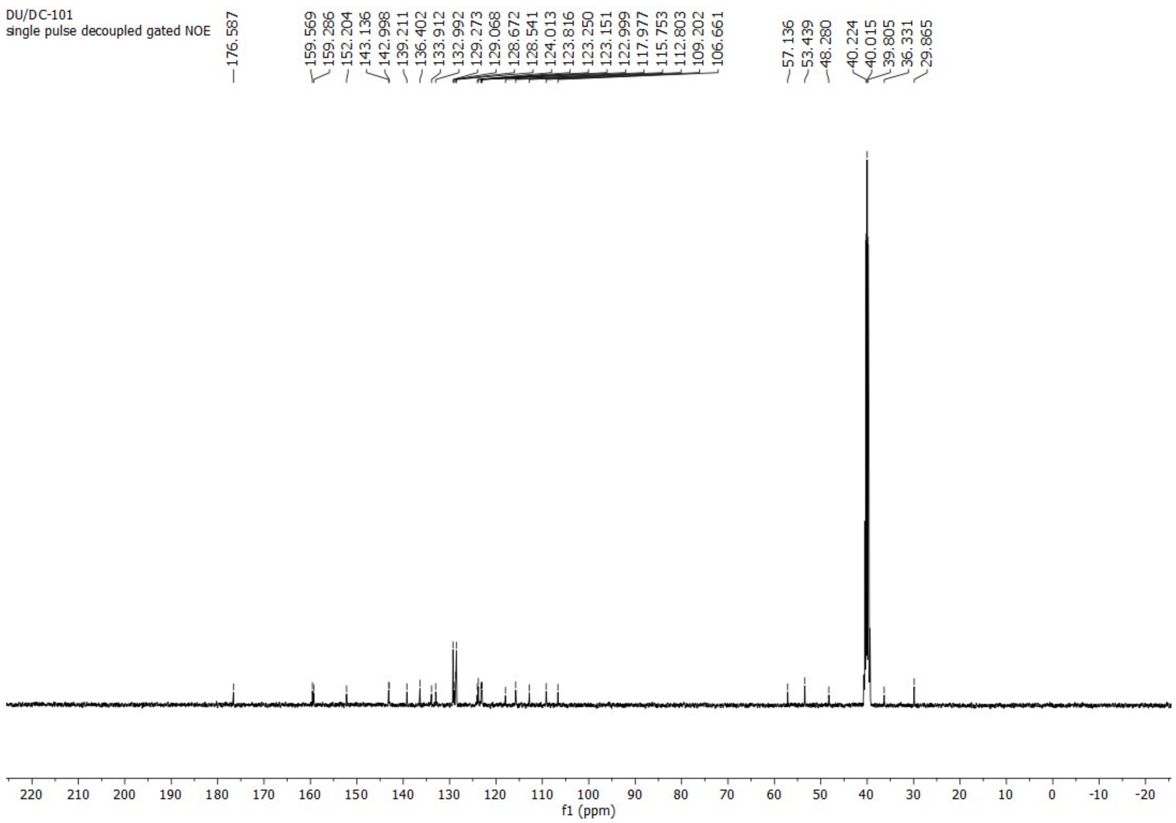

Figure S4. ^{13}C NMR spectrum of compound **4a** at 150 MHz in DMSO-d6

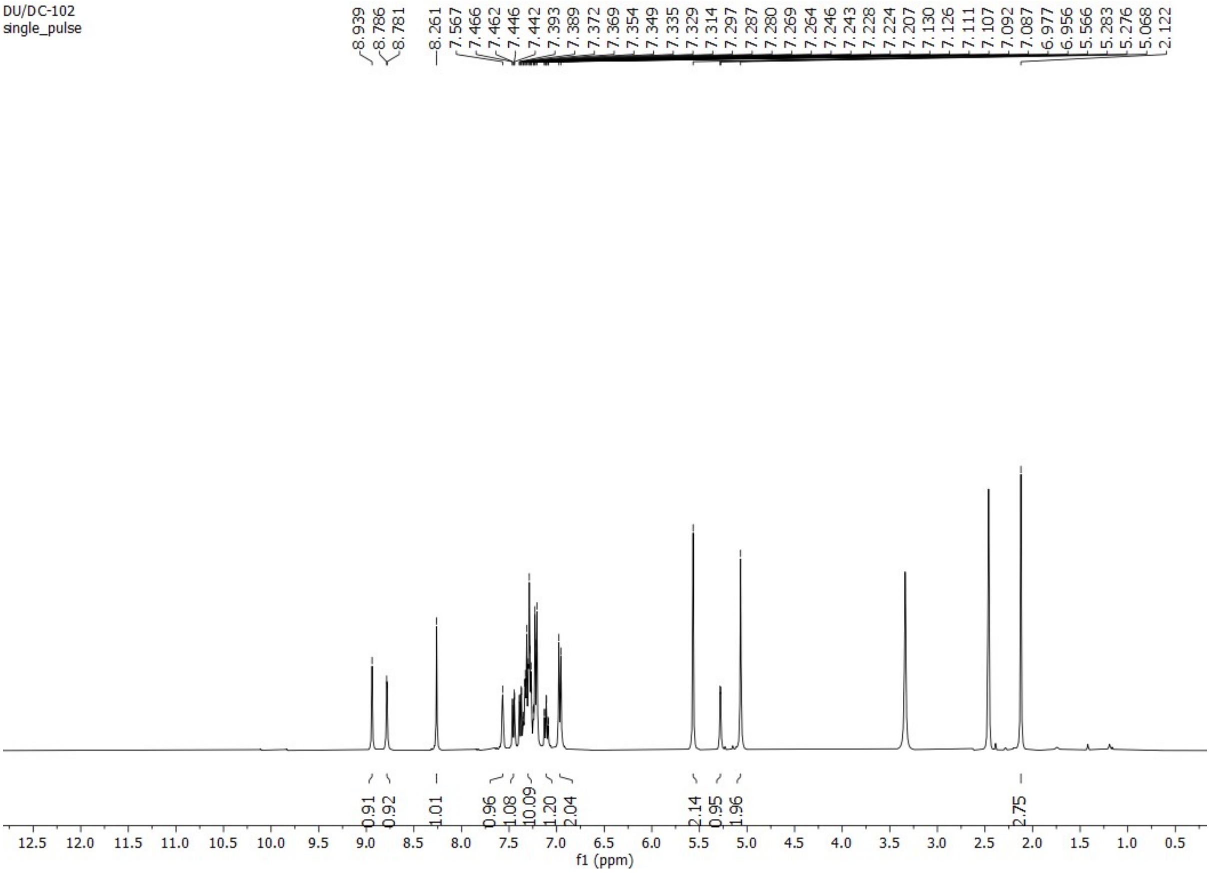

Figure S5. ^{1}H NMR spectrum of compound **4b** at 400 MHz in DMSO-d6

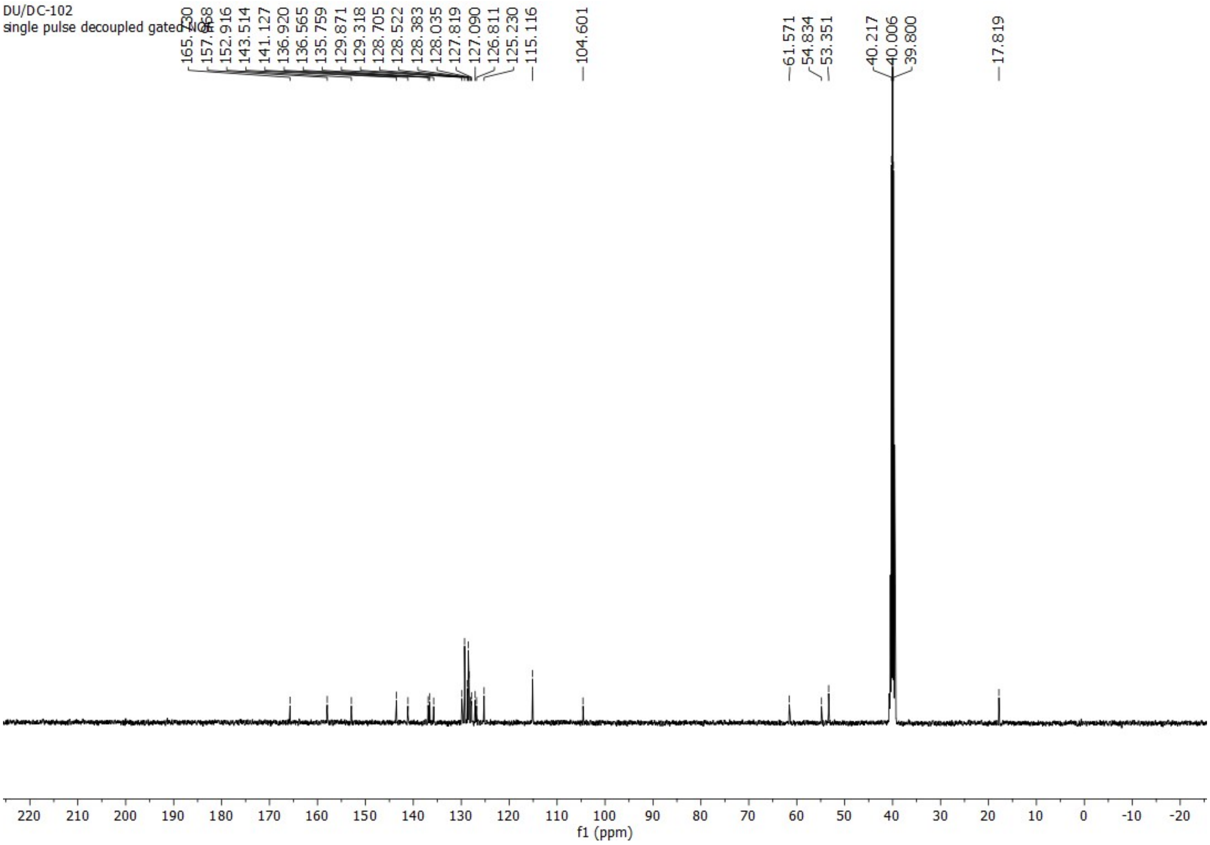

Figure S6. ^{13}C NMR spectrum of compound **4b** at 150 MHz in DMSO-d6

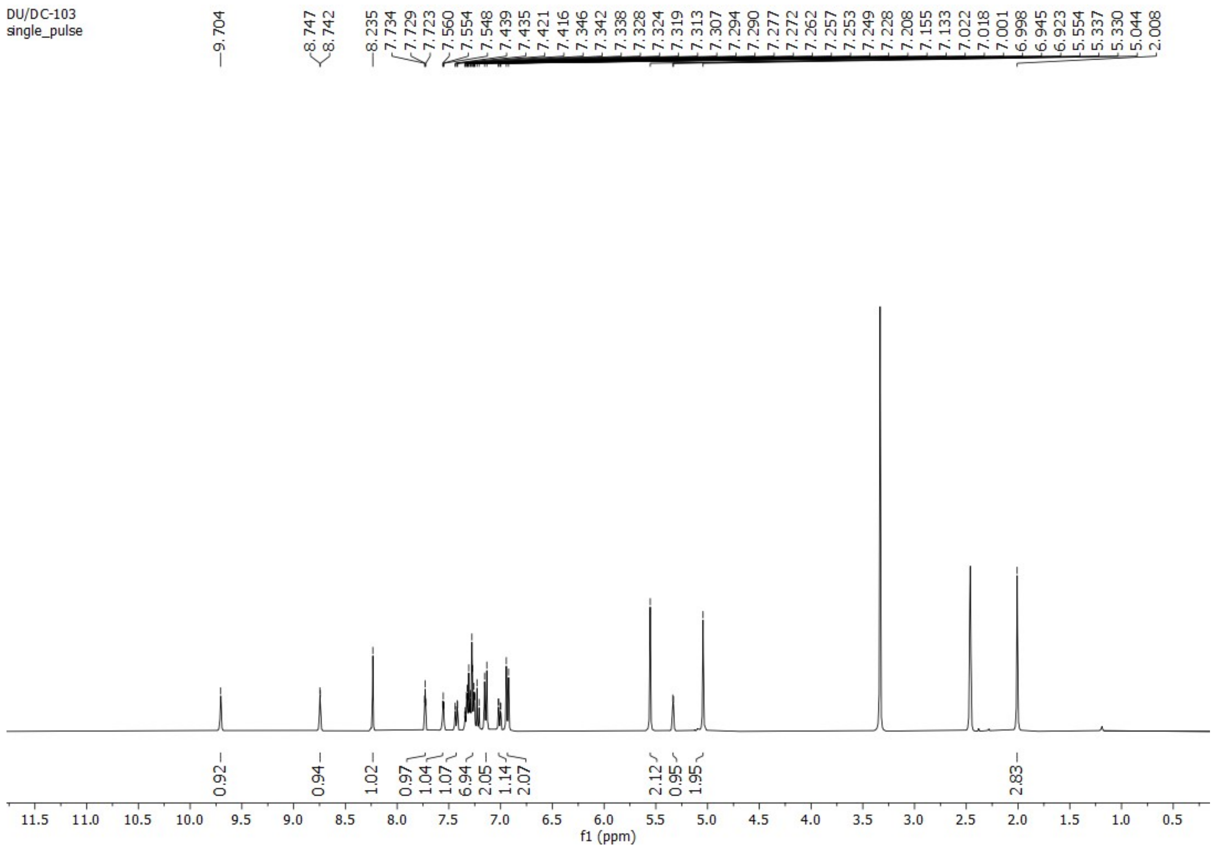

Figure S7. ^{1}H NMR spectrum of compound **4c** at 400 MHz in DMSO-d6

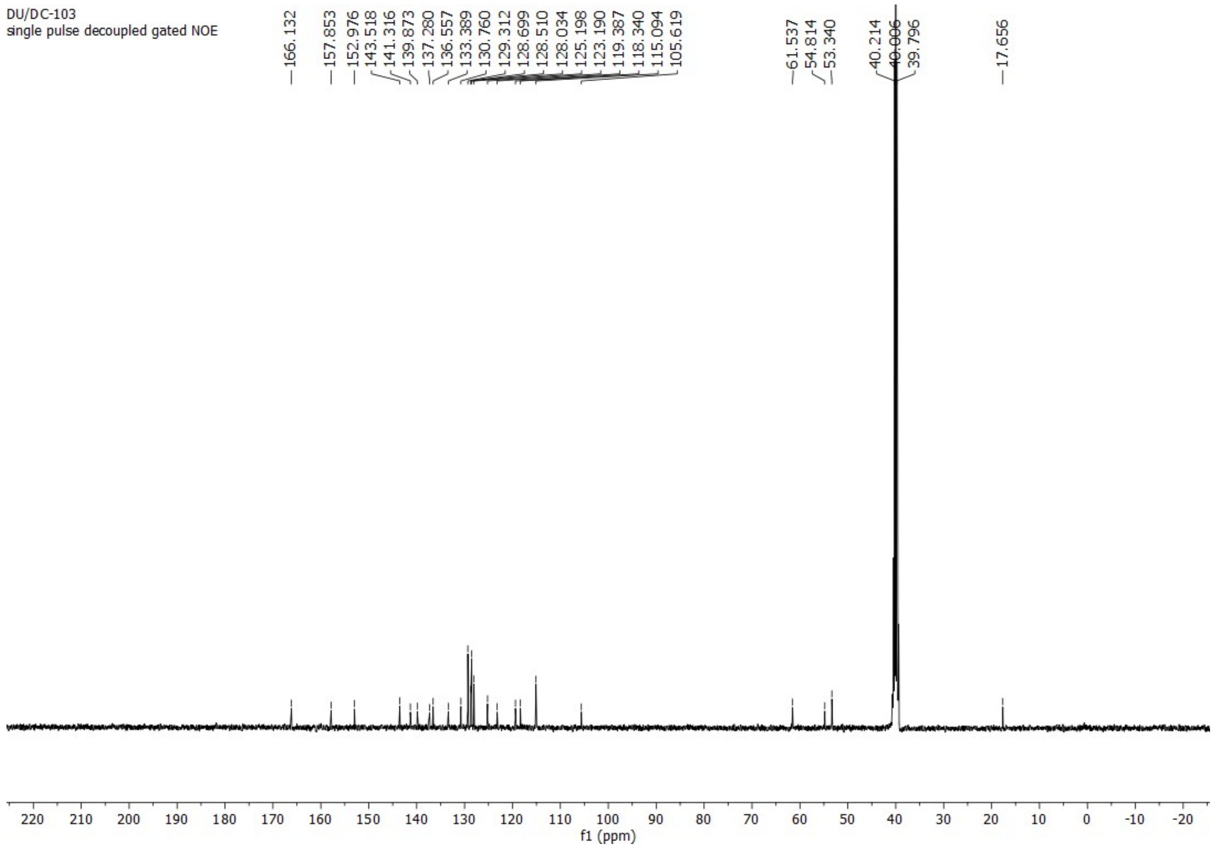

Figure S8. ^{13}C NMR spectrum of compound **4c** at 150 MHz in DMSO-d6

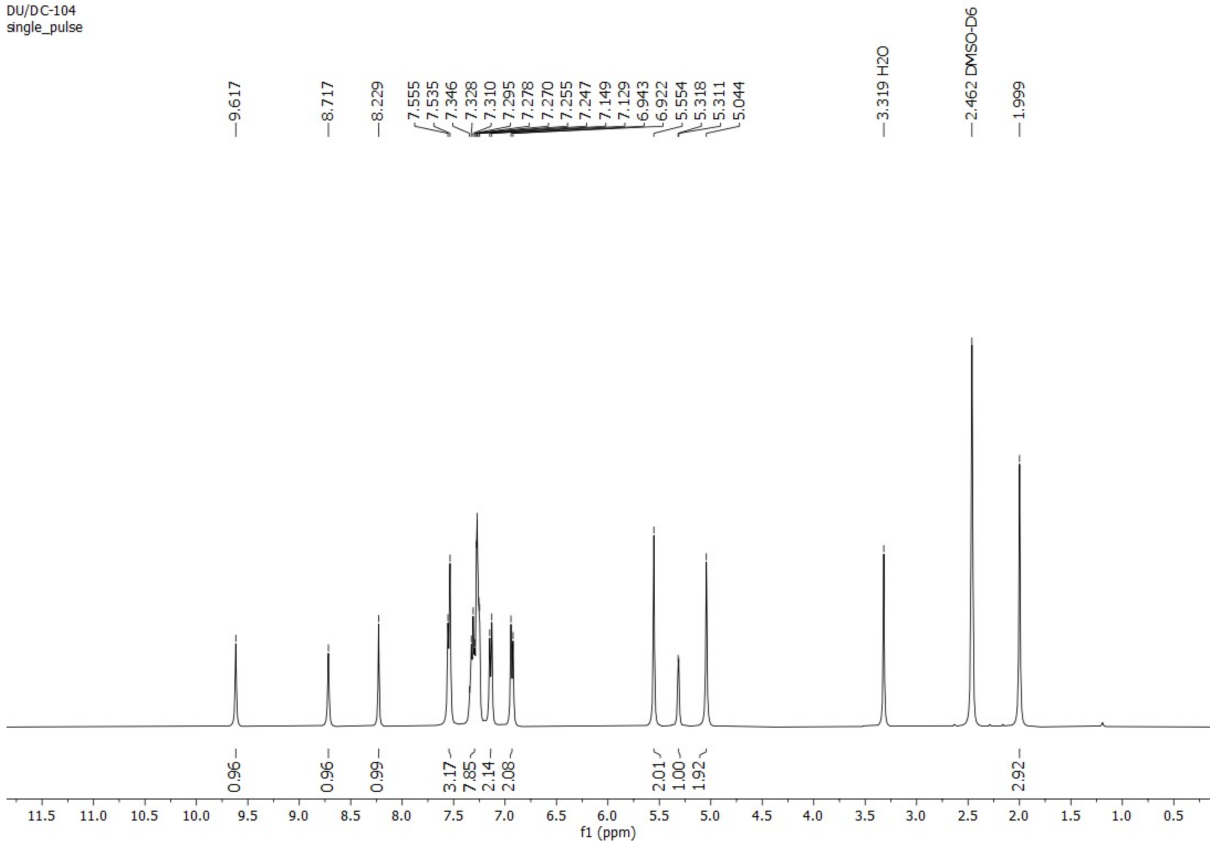

Figure S9. ^{1}H NMR spectrum of compound **4d** at 400 MHz in DMSO-d6

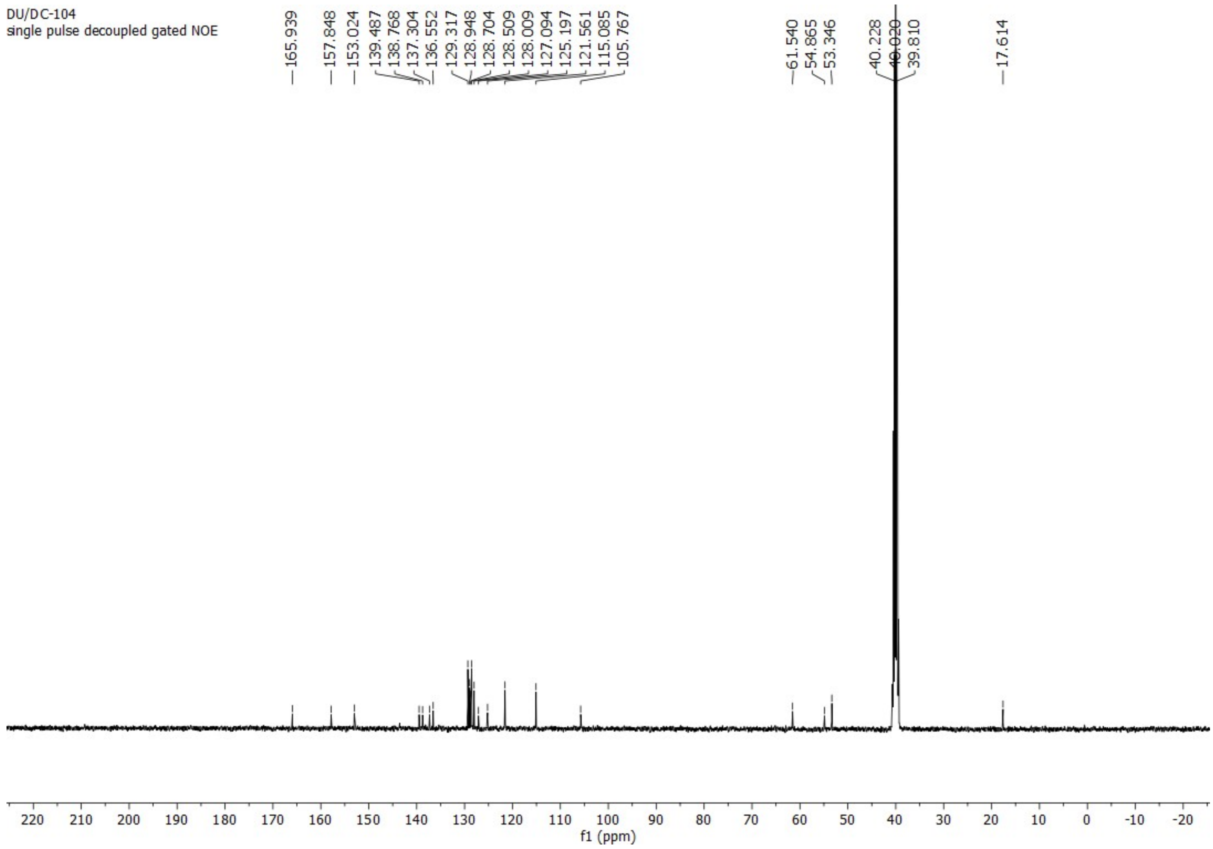

Figure S10. ^{13}C NMR spectrum of compound **4d** at 150 MHz in DMSO-d6

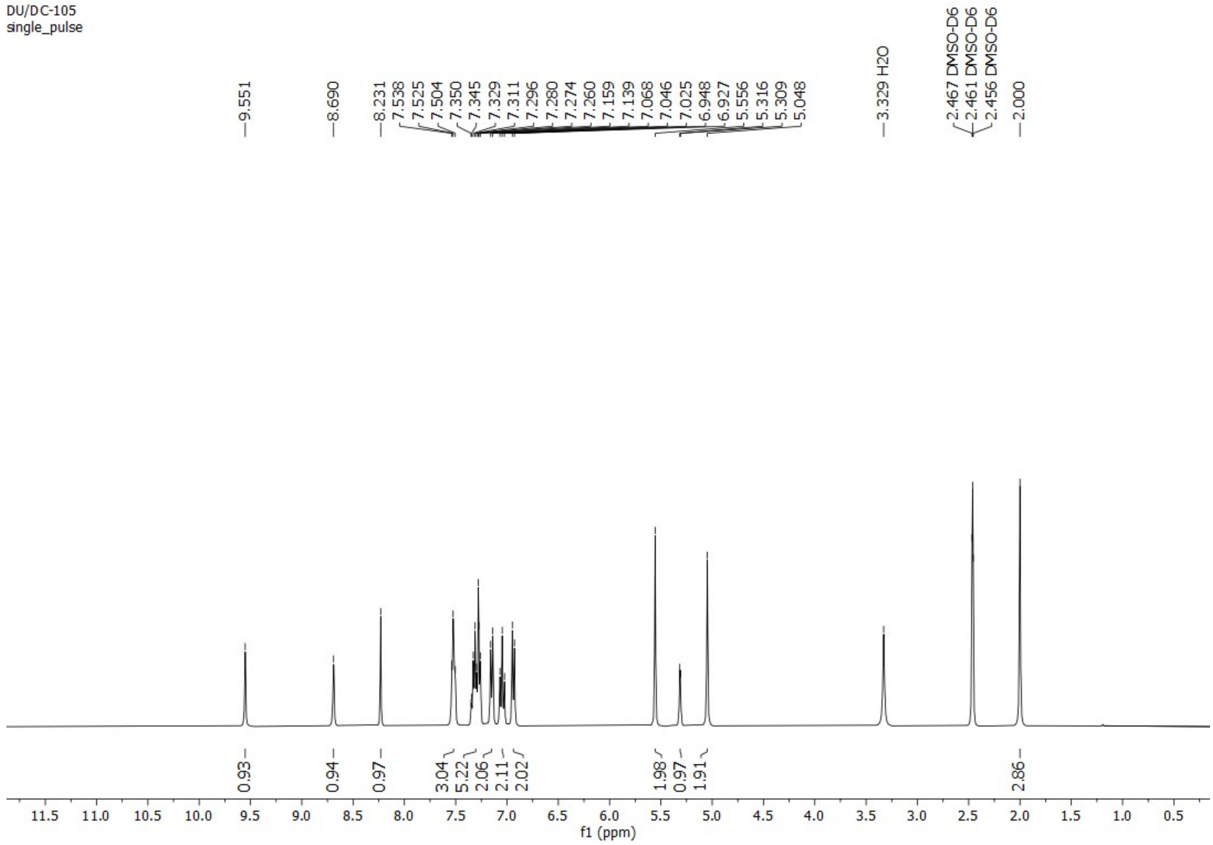

Figure S11. ^{1}H NMR spectrum of compound **4e** at 400 MHz in DMSO-d6

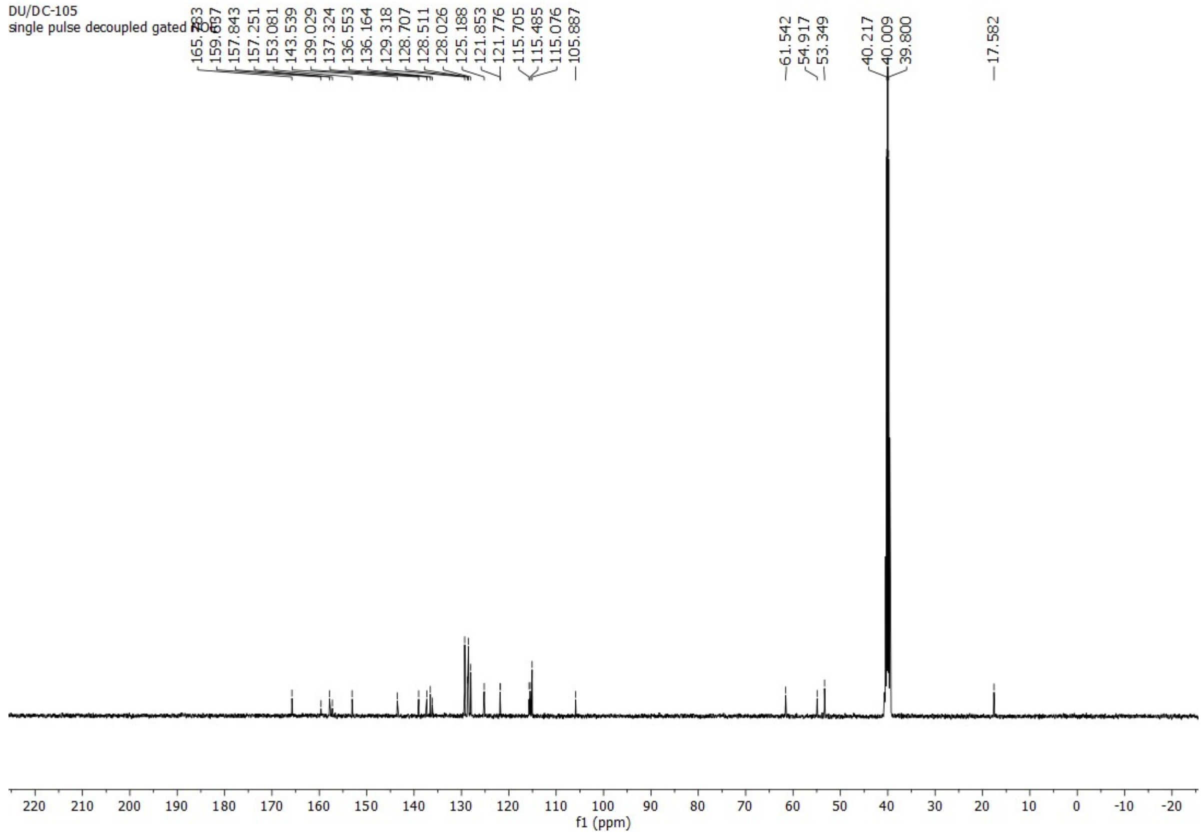

Figure S12. ^{13}C NMR spectrum of compound **4e** at 150 MHz in DMSO-d6

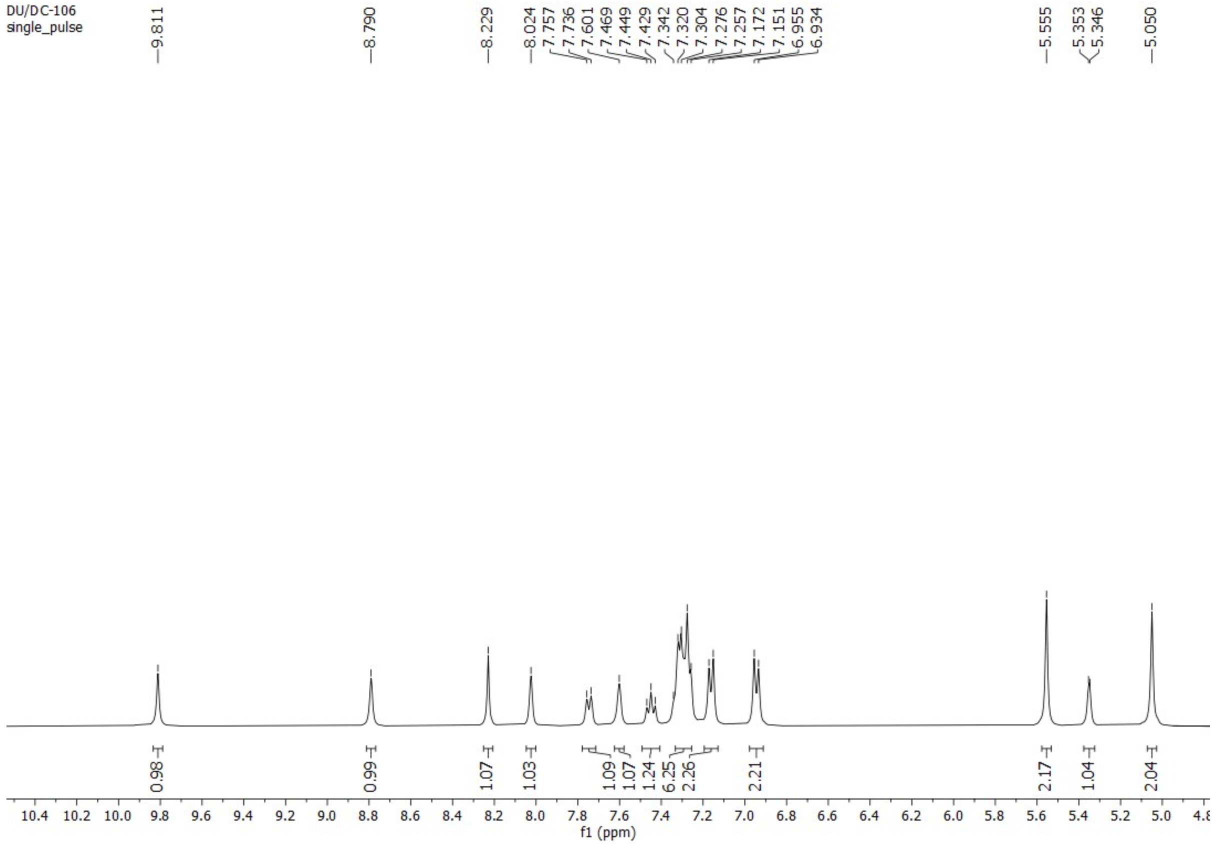

Figure S13. ^{1}H NMR spectrum of compound **4f** at 400 MHz in DMSO-d6

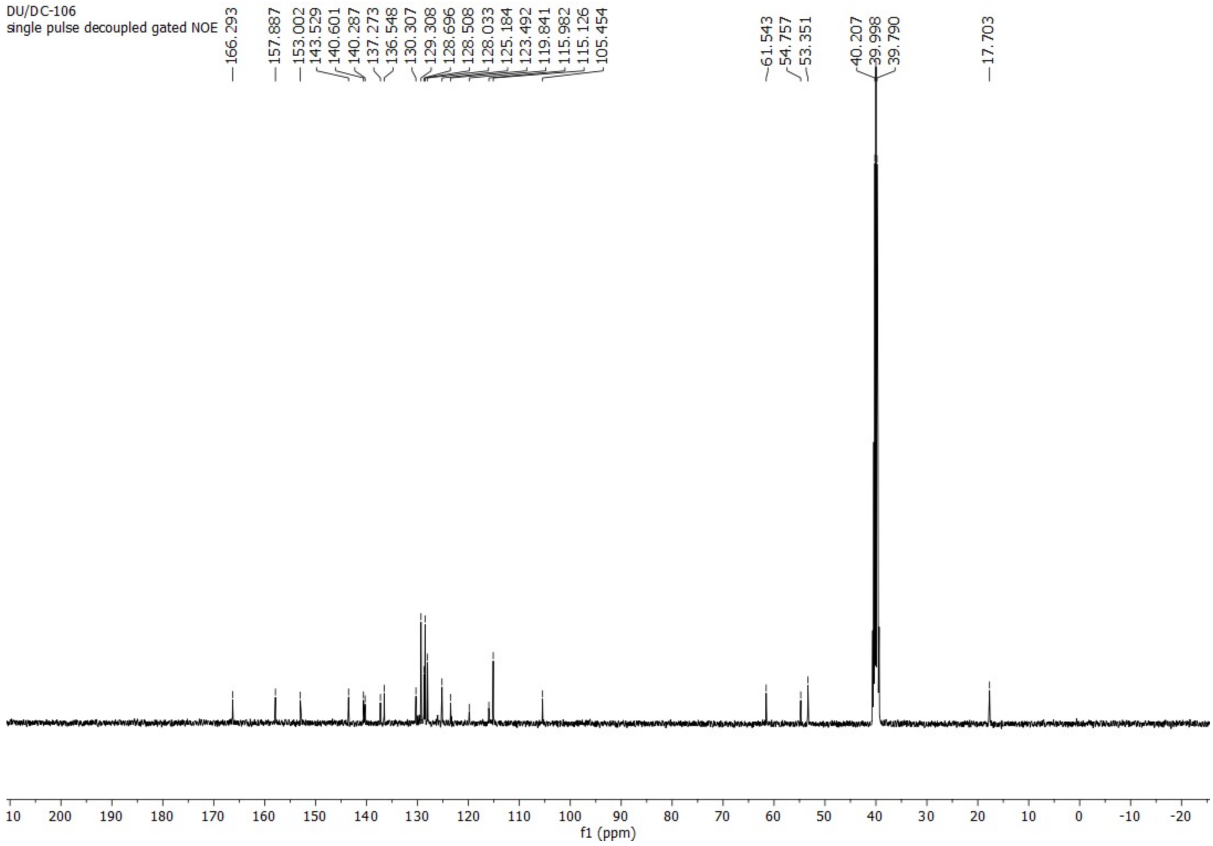

Figure S14. ^{13}C NMR spectrum of compound **4f** at 150 MHz in DMSO-d_6

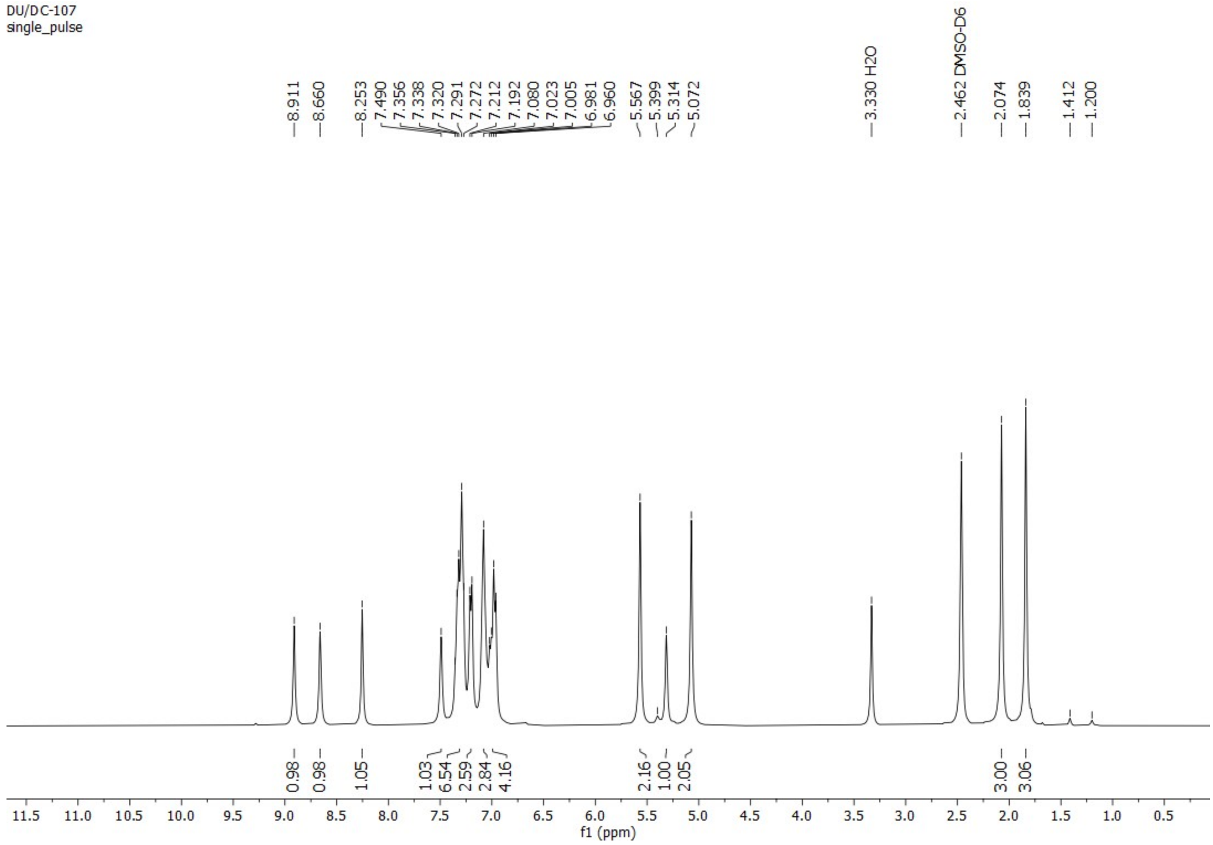

Figure S15. ^{1}H NMR spectrum of compound **4g** at 400 MHz in DMSO-d_6

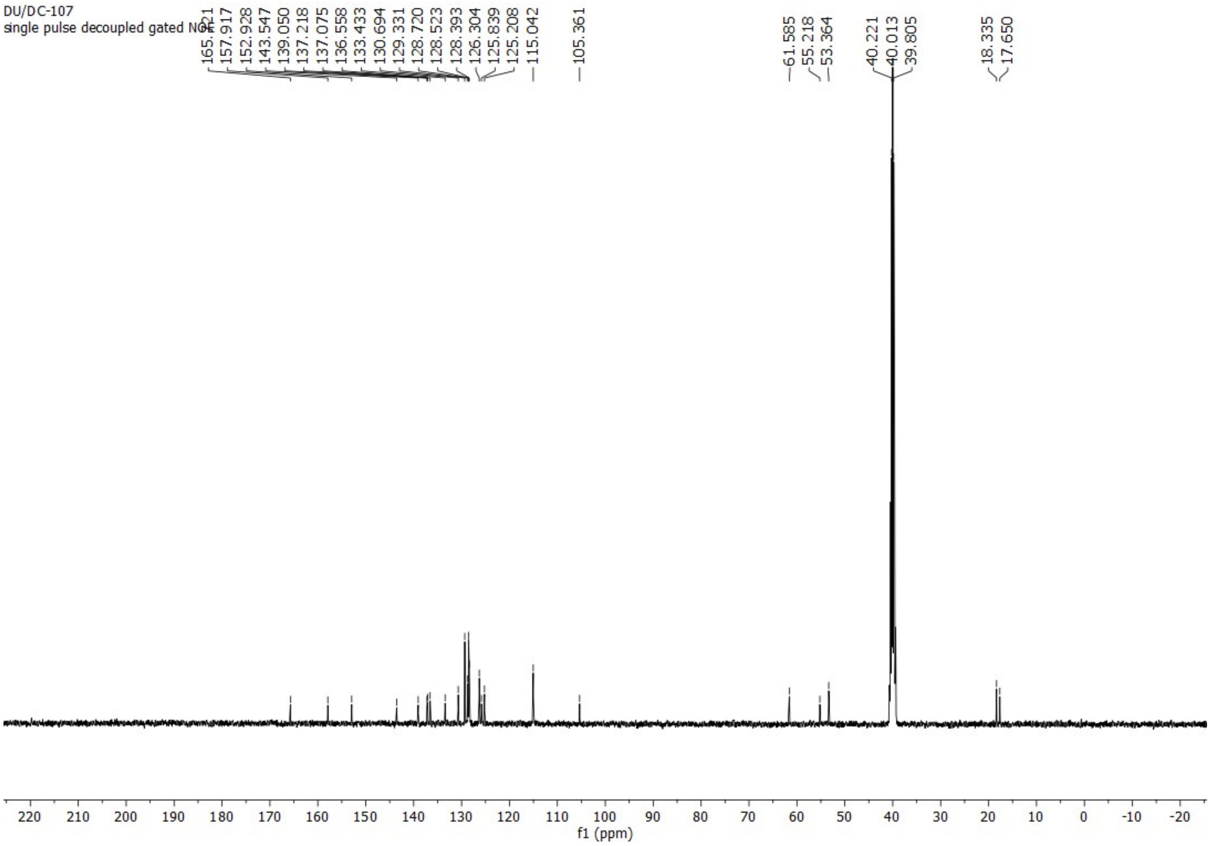

Figure S16. ^{13}C NMR spectrum of compound **4g** at 150 MHz in DMSO-d6

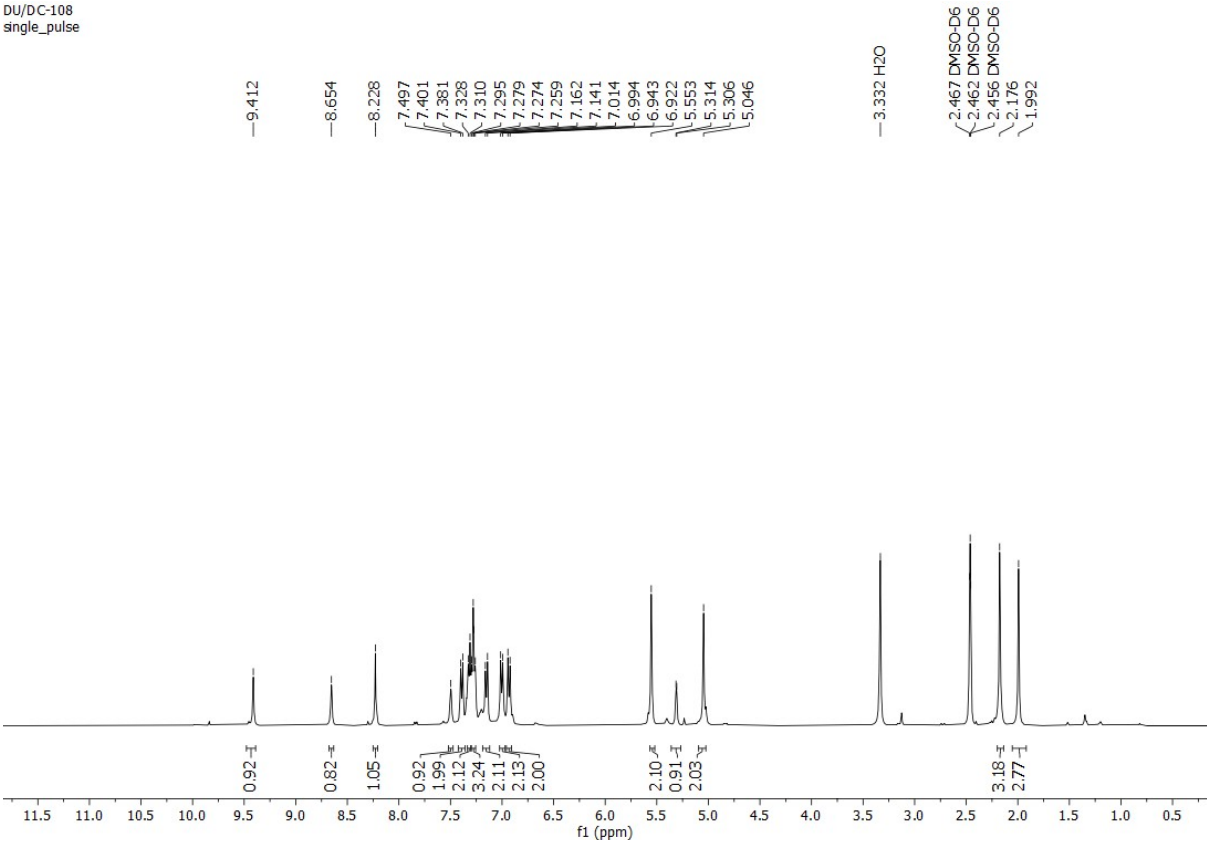

Figure S17. ^{1}H NMR spectrum of compound **4h** at 400 MHz in DMSO-d6

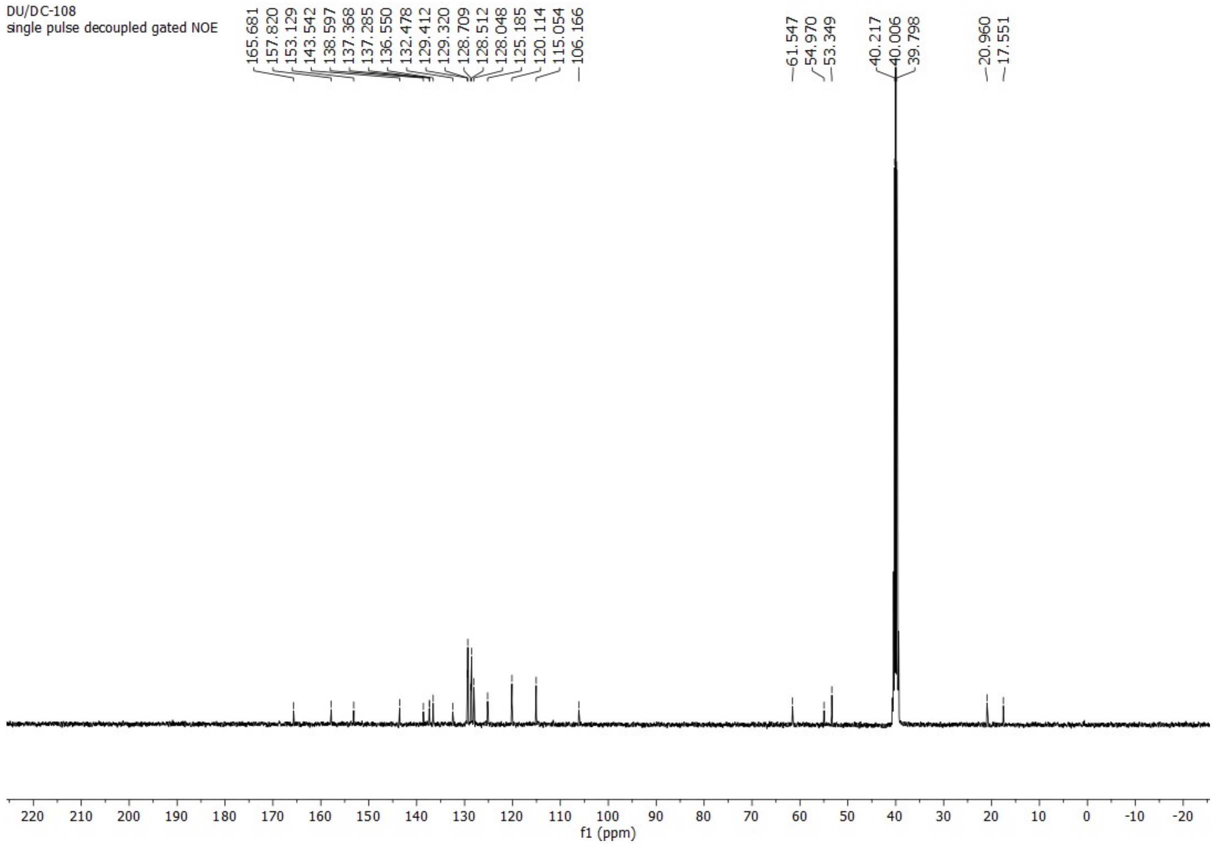

Figure S18. ^{13}C NMR spectrum of compound **4h** at 150 MHz in DMSO-d6

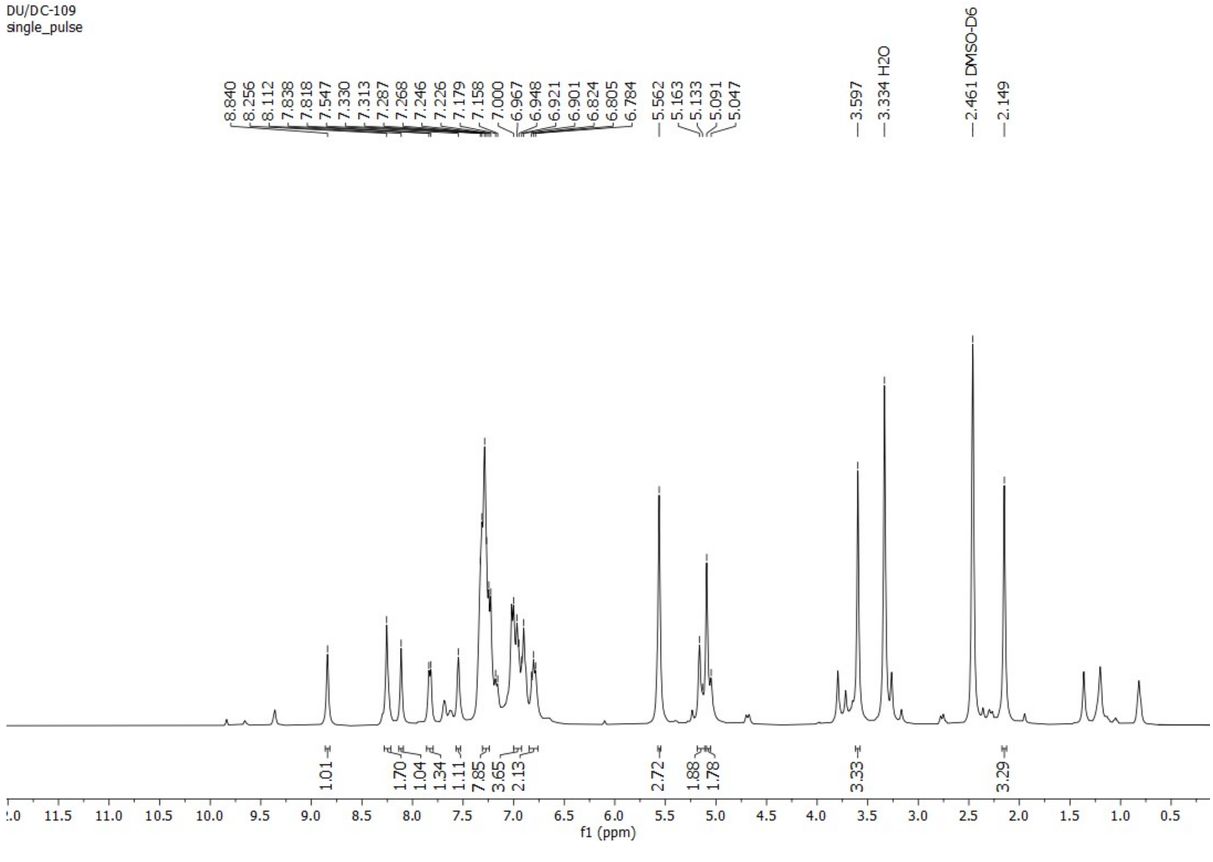

Figure S19. ^{1}H NMR spectrum of compound **4i** at 400 MHz in DMSO-d6

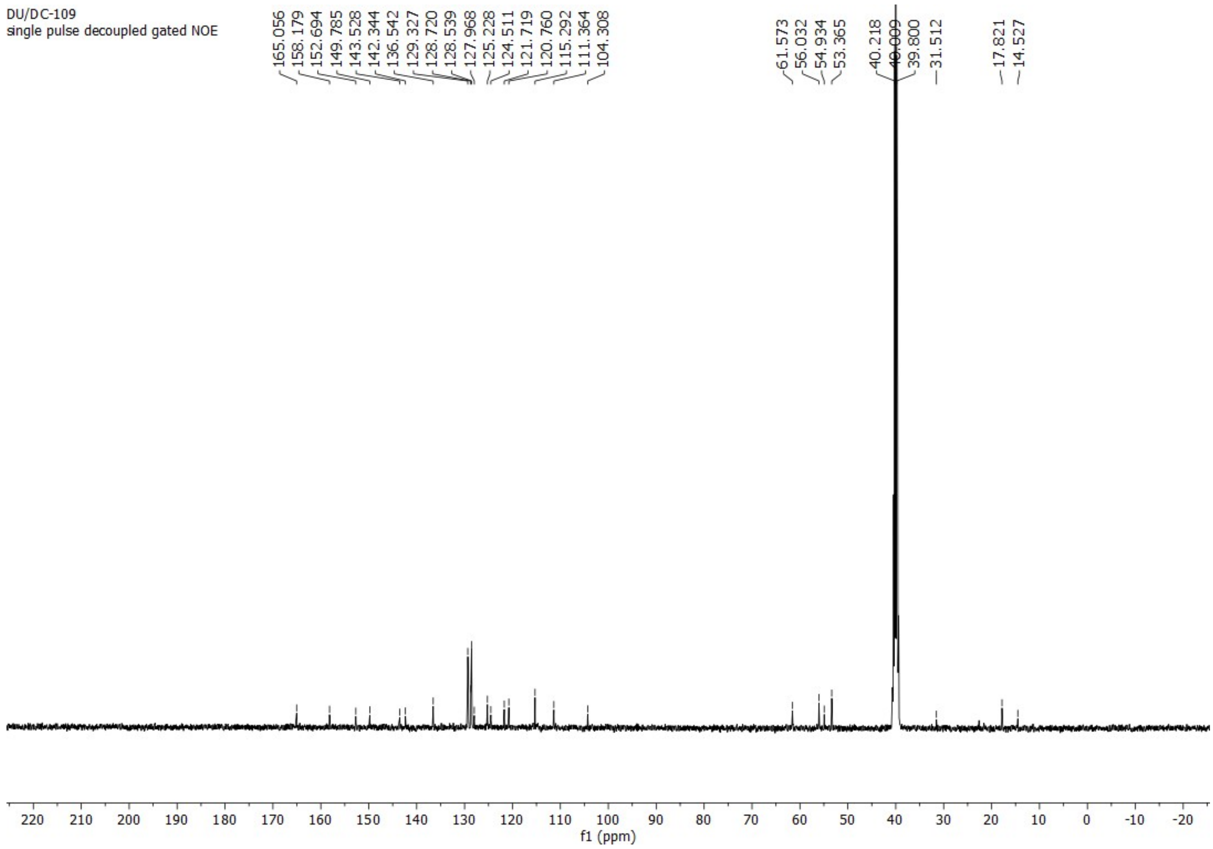

Figure S20. ^{13}C NMR spectrum of compound **4i** at 150 MHz in DMSO-d6

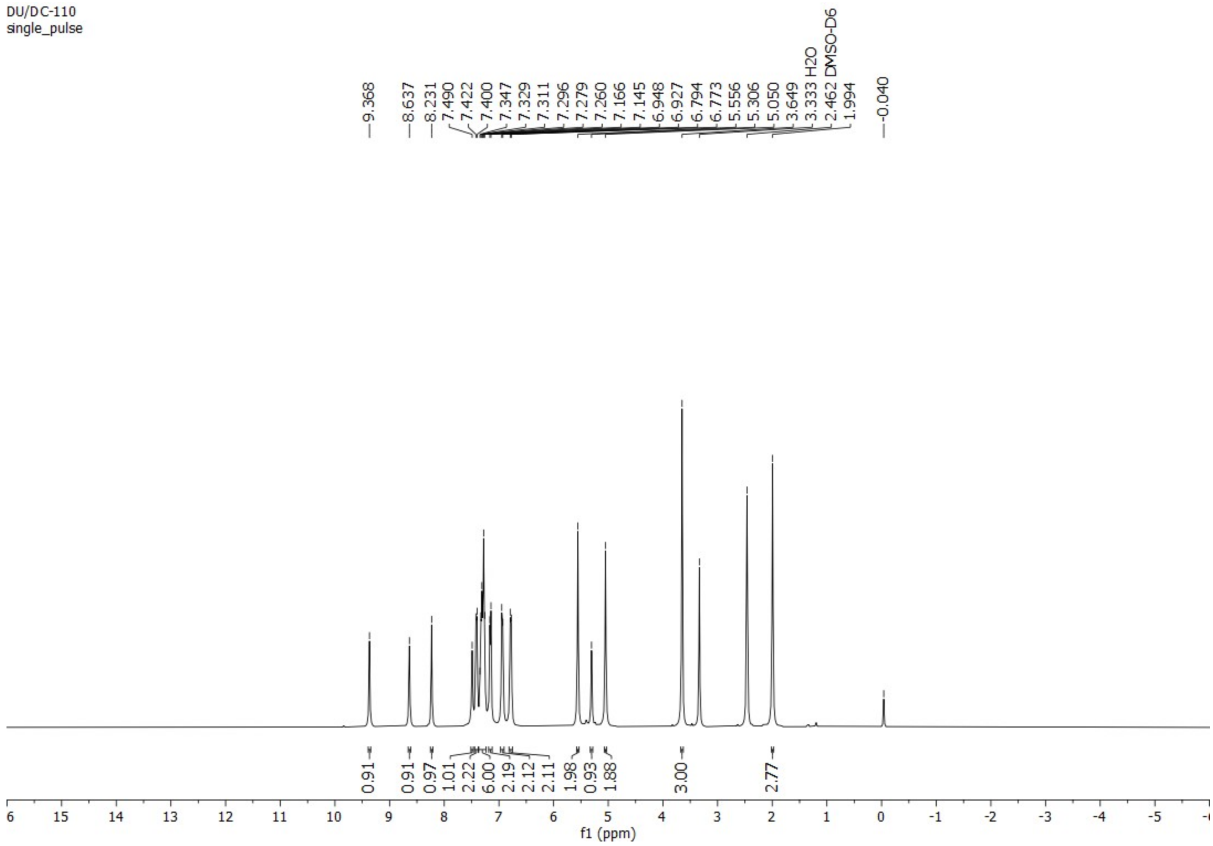

Figure S21. ^{1}H NMR spectrum of compound **4j** at 400 MHz in DMSO-d6

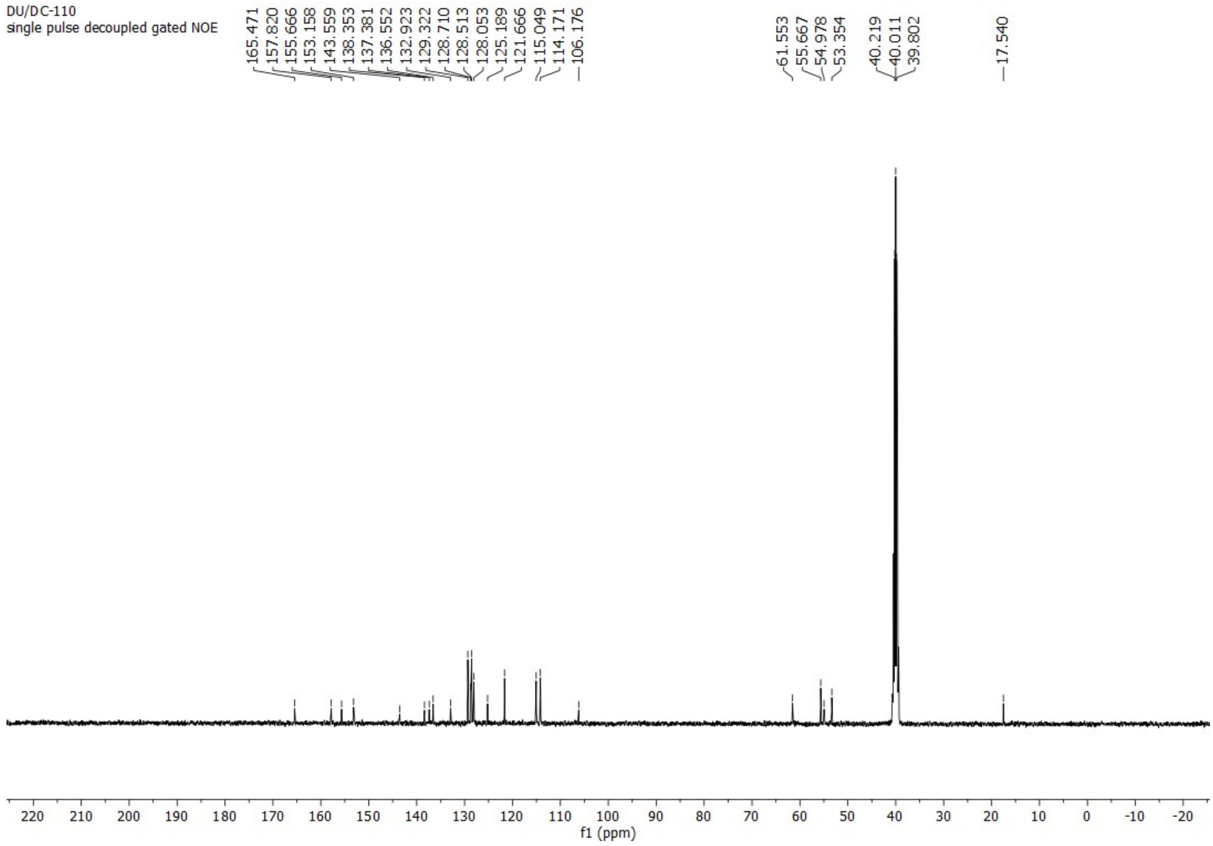

Figure S22. ^{13}C NMR spectrum of compound **4j** at 150 MHz in DMSO-d6

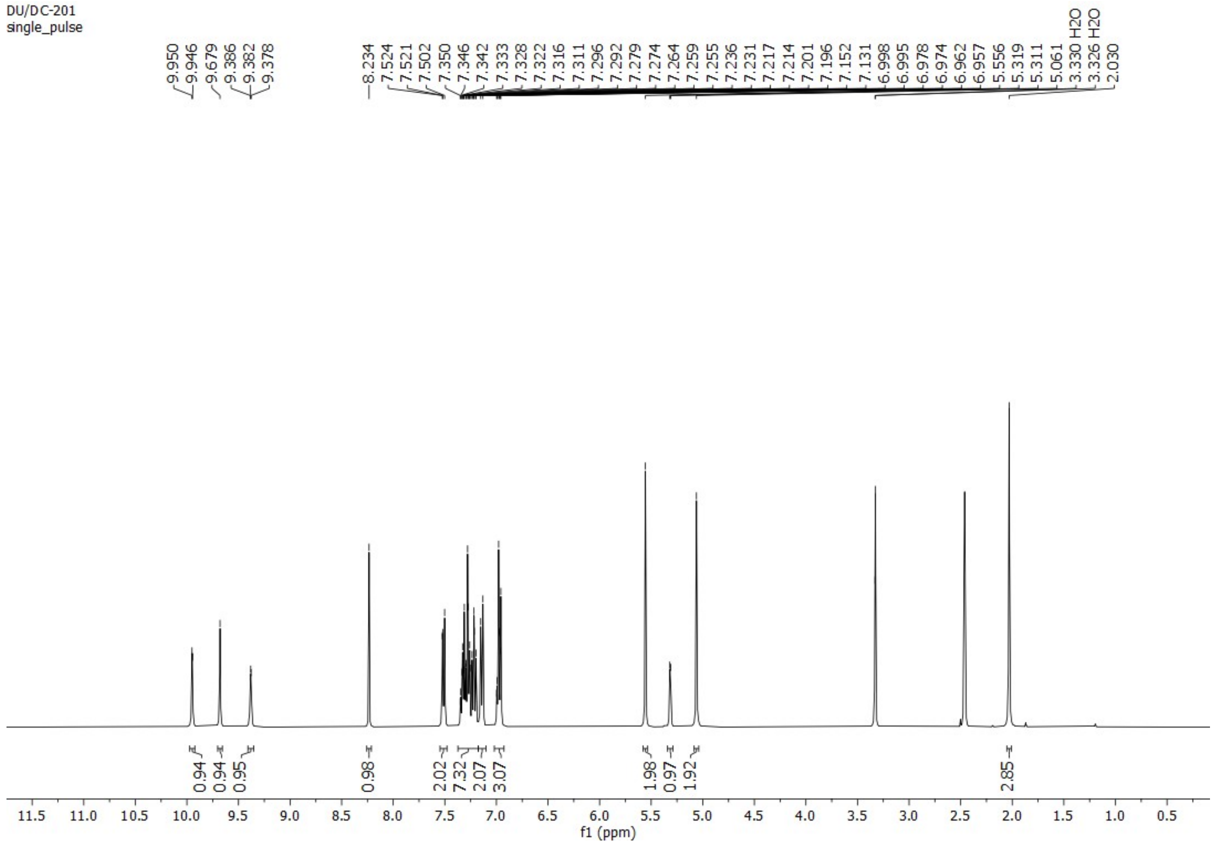

Figure S23. ^{1}H NMR spectrum of compound **4k** at 400 MHz in DMSO-d6

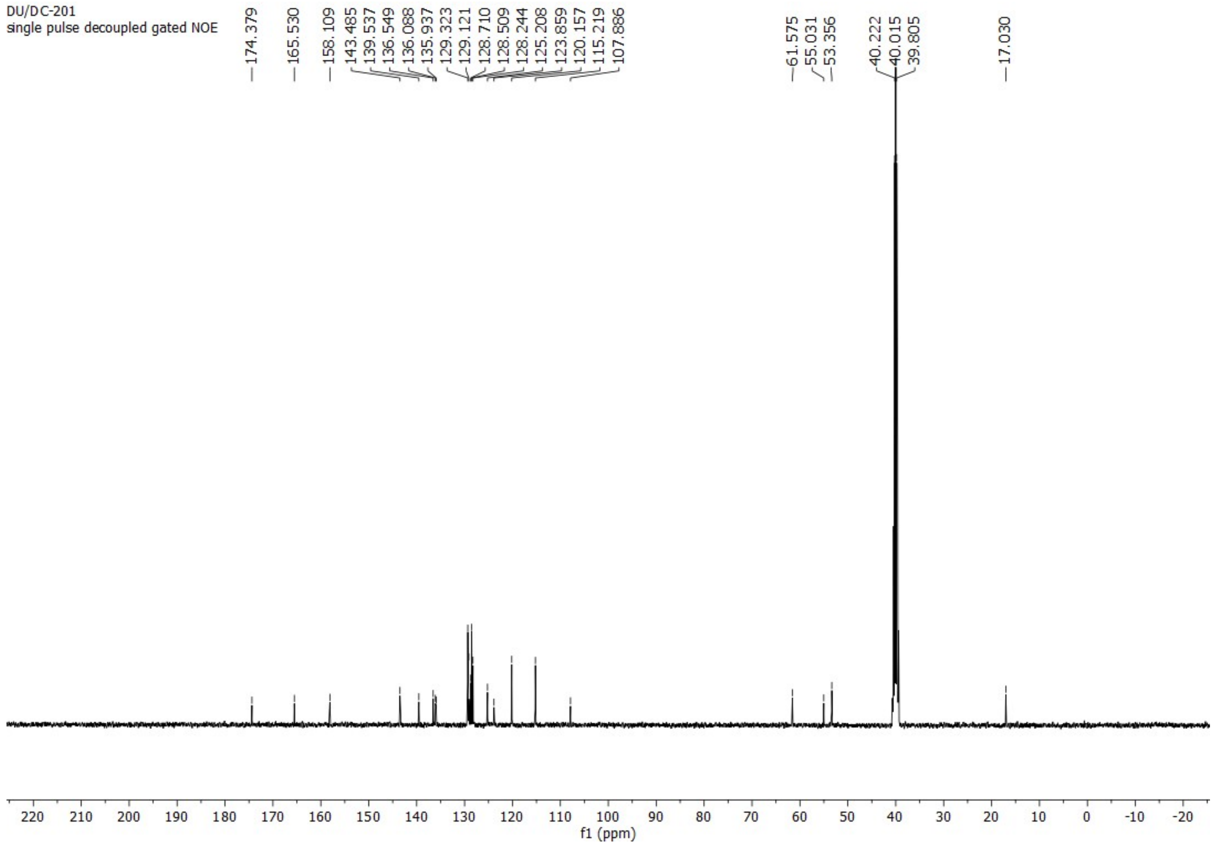

Figure S24. ^{13}C NMR spectrum of compound **4k** at 150 MHz in DMSO-d6

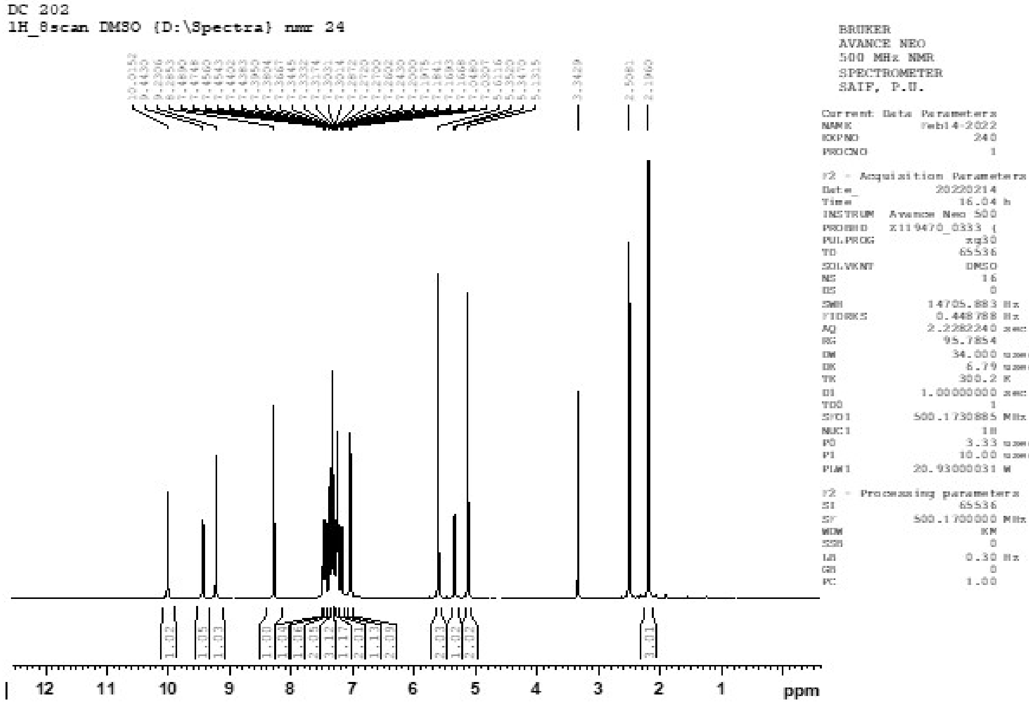

Figure S25. ^{1}H NMR spectrum of compound **4l** at 400 MHz in DMSO-d6

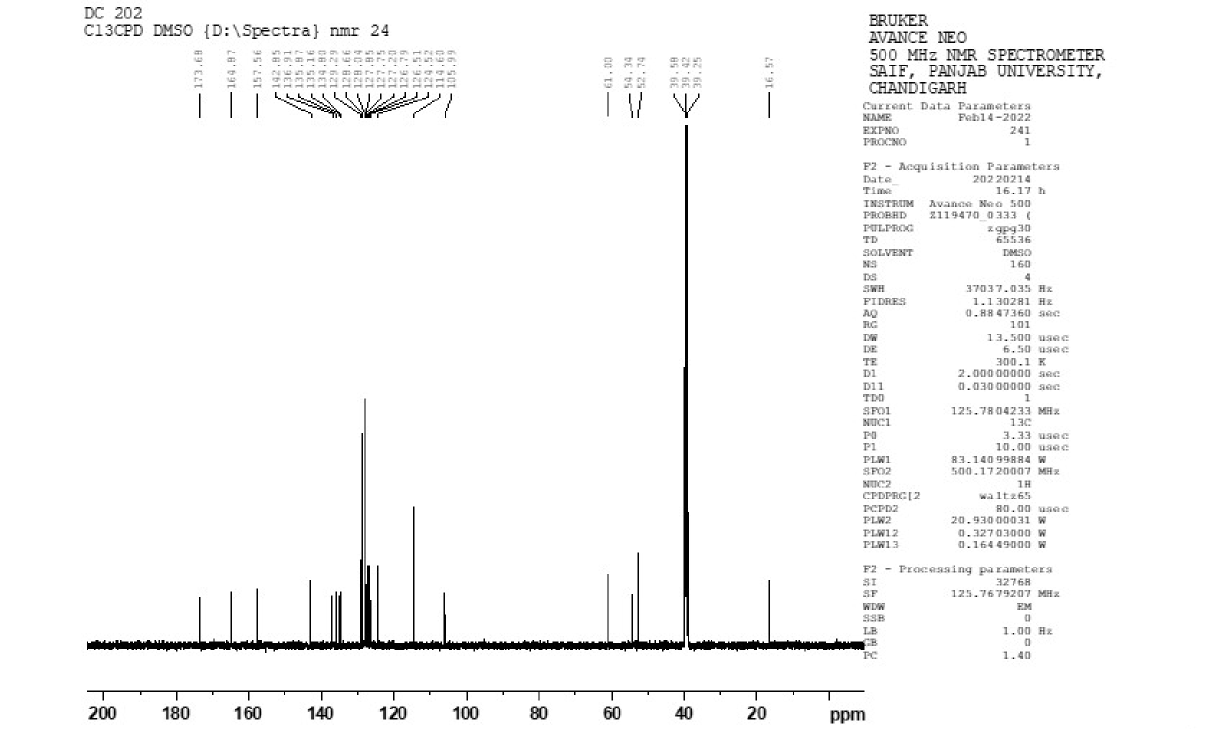

Figure S26. ^{13}C NMR spectrum of compound **4l** at 150 MHz in DMSO-d6

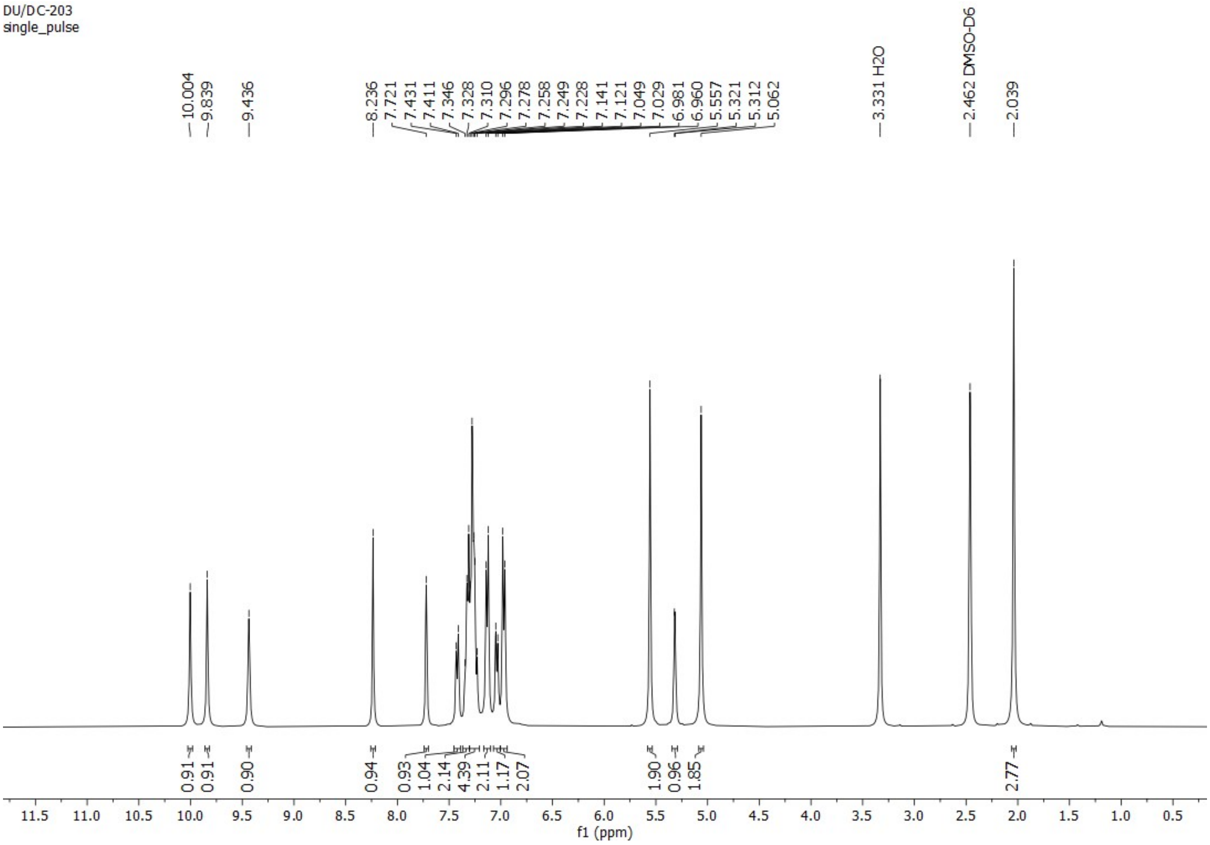

Figure S27. ^{1}H NMR spectrum of compound **4m** at 400 MHz in DMSO-d6

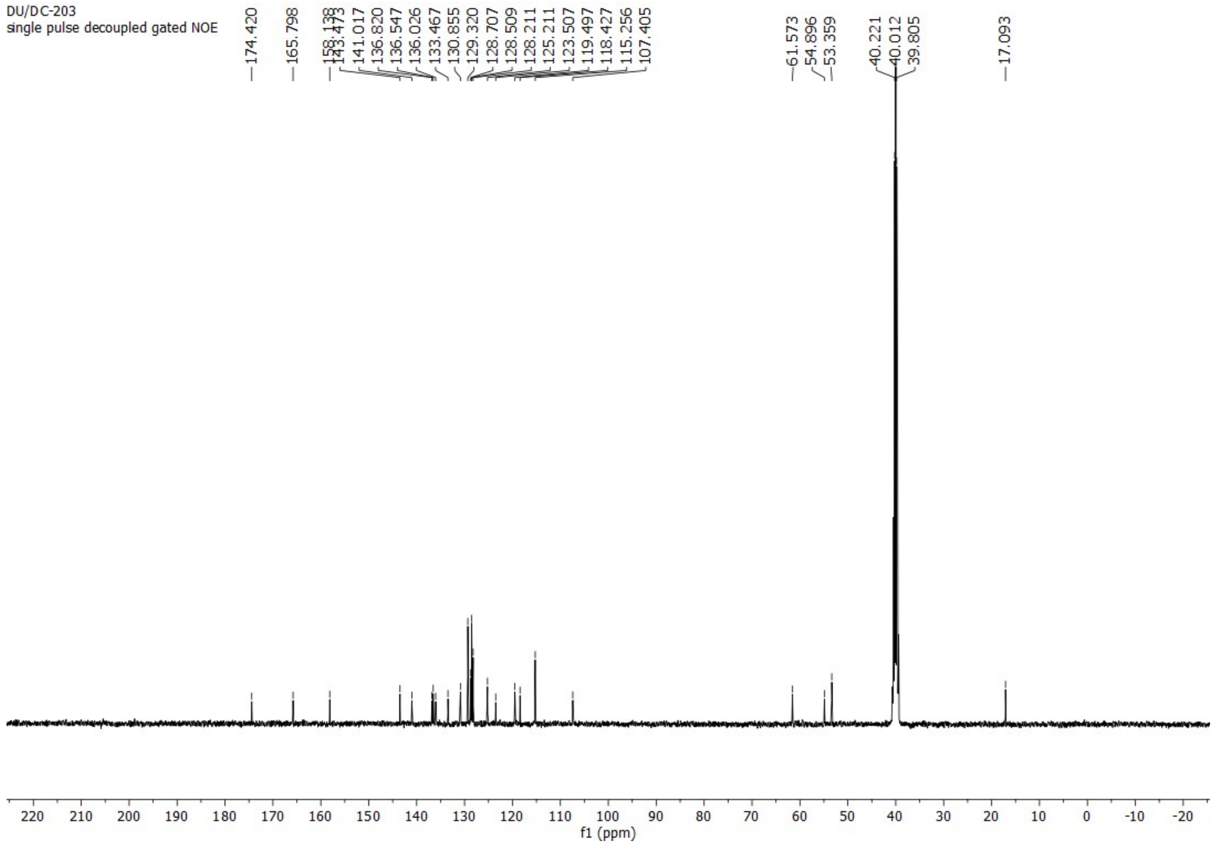

Figure S28. ^{13}C NMR spectrum of compound **4m** at 150 MHz in DMSO-d6

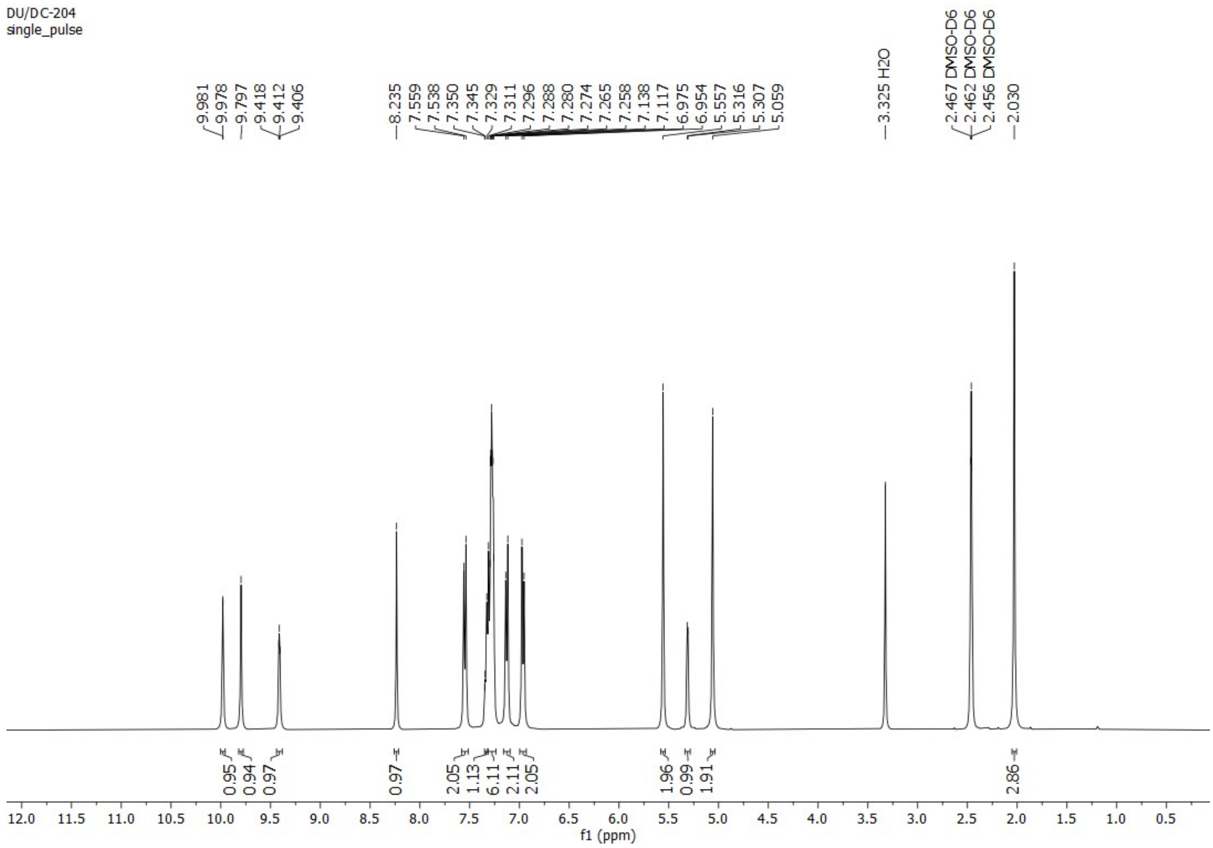

Figure S29. ^{1}H NMR spectrum of compound **4n** at 400 MHz in DMSO-d6

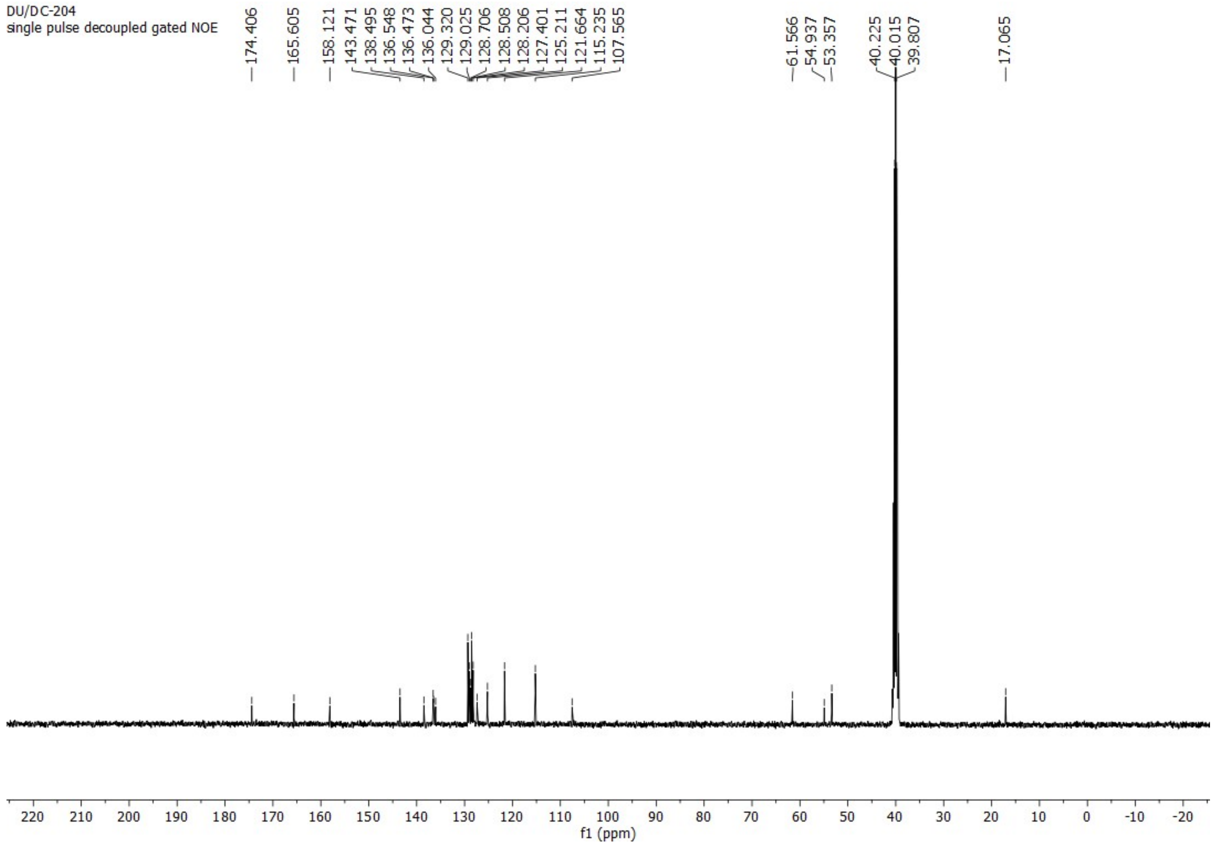

Figure S30. ^{13}C NMR spectrum of compound **4n** at 150 MHz in DMSO-d6

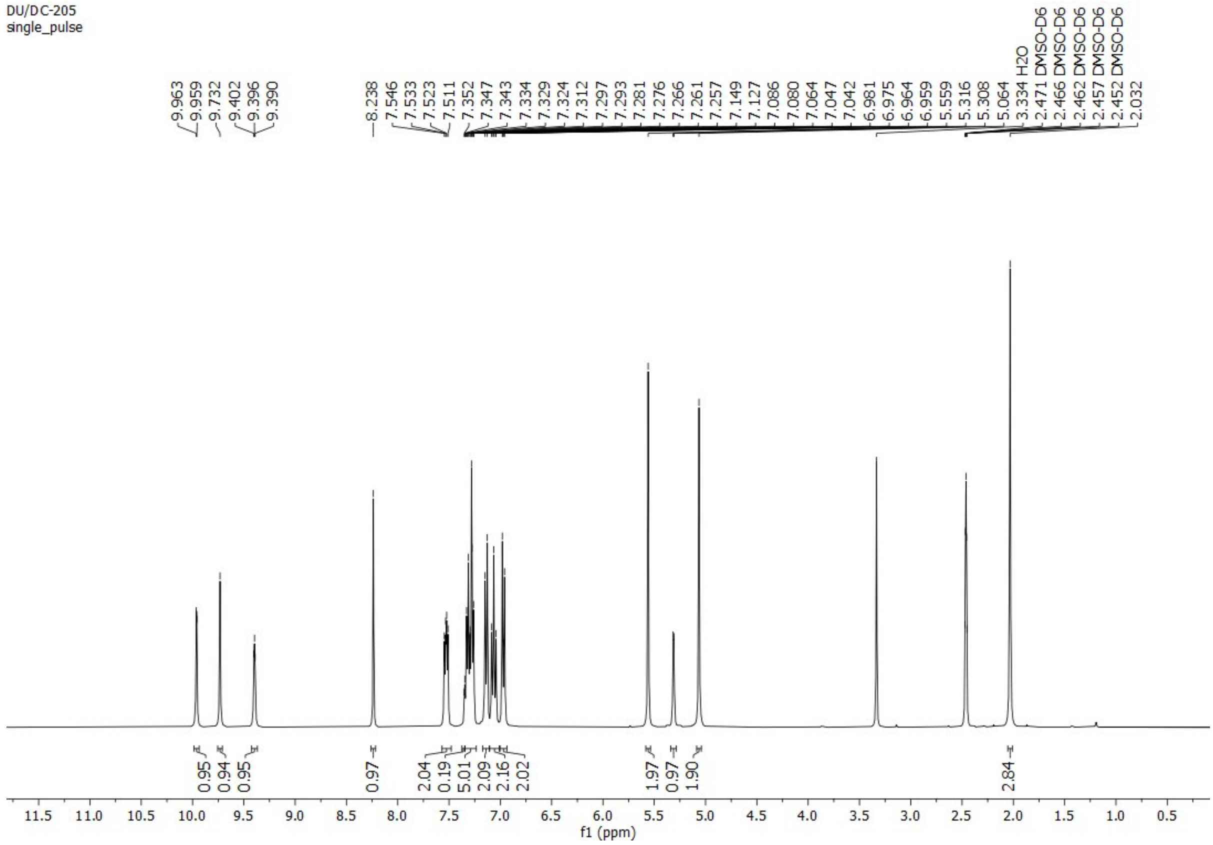

Figure S31. ^{1}H NMR spectrum of compound **4o** at 400 MHz in DMSO-d6

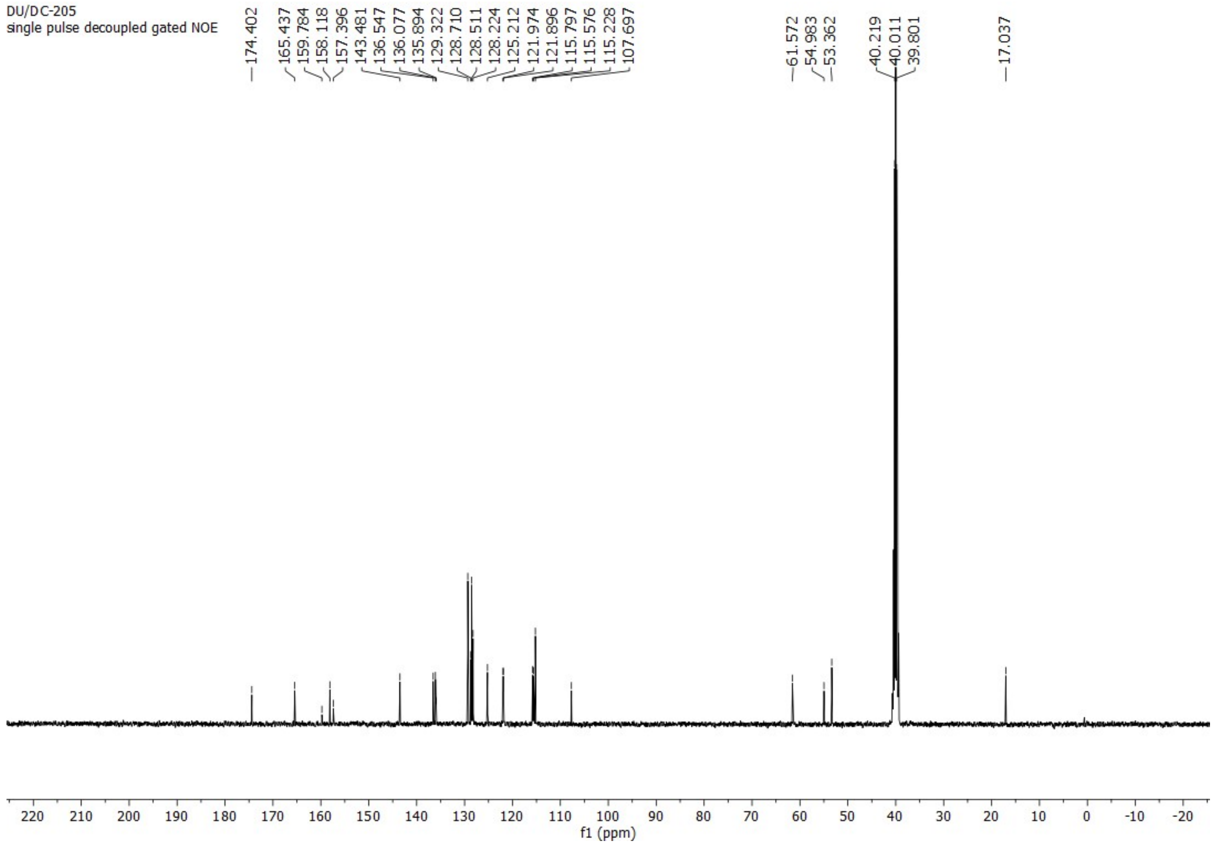

Figure S32. ^{13}C NMR spectrum of compound **4o** at 150 MHz in DMSO-d$_6$

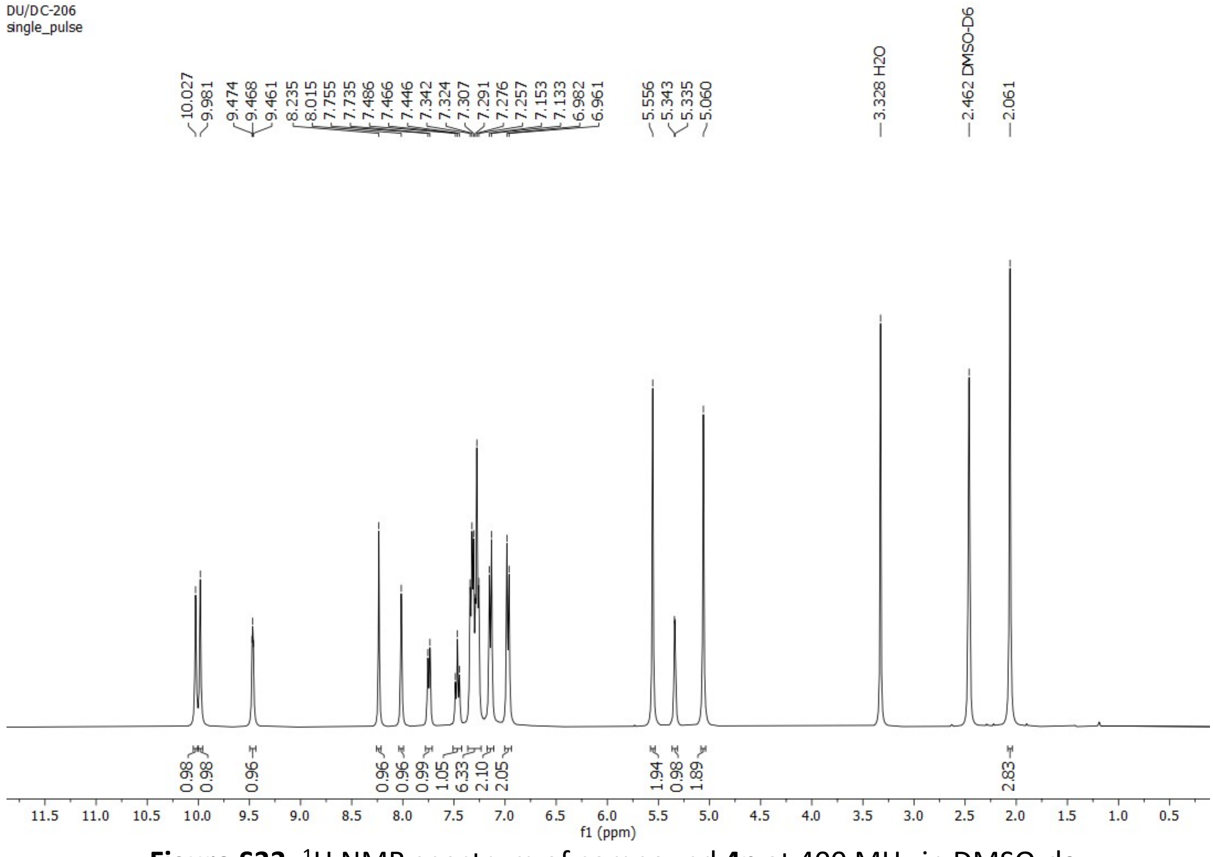

Figure S33. ^{1}H NMR spectrum of compound **4p** at 400 MHz in DMSO-d$_6$

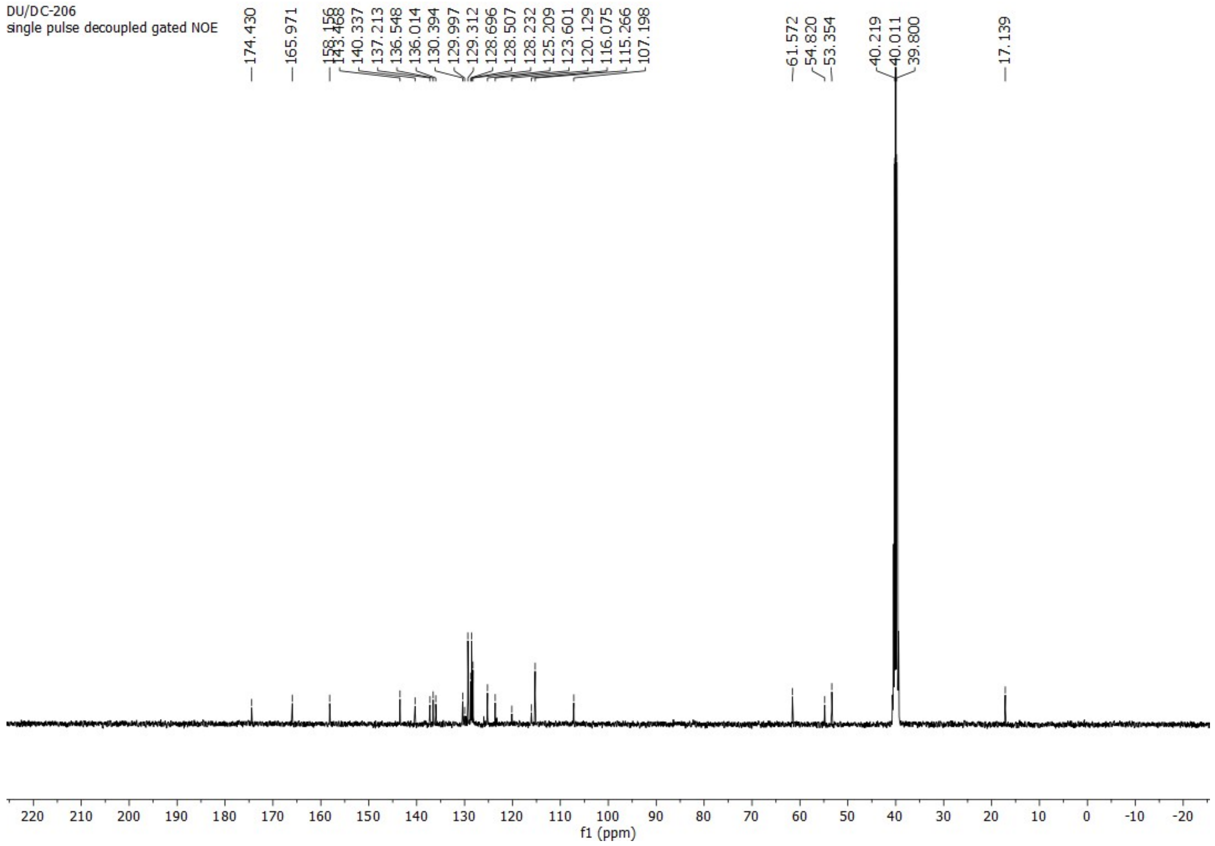

Figure S34. ^{13}C NMR spectrum of compound **4p** at 150 MHz in DMSO-d6

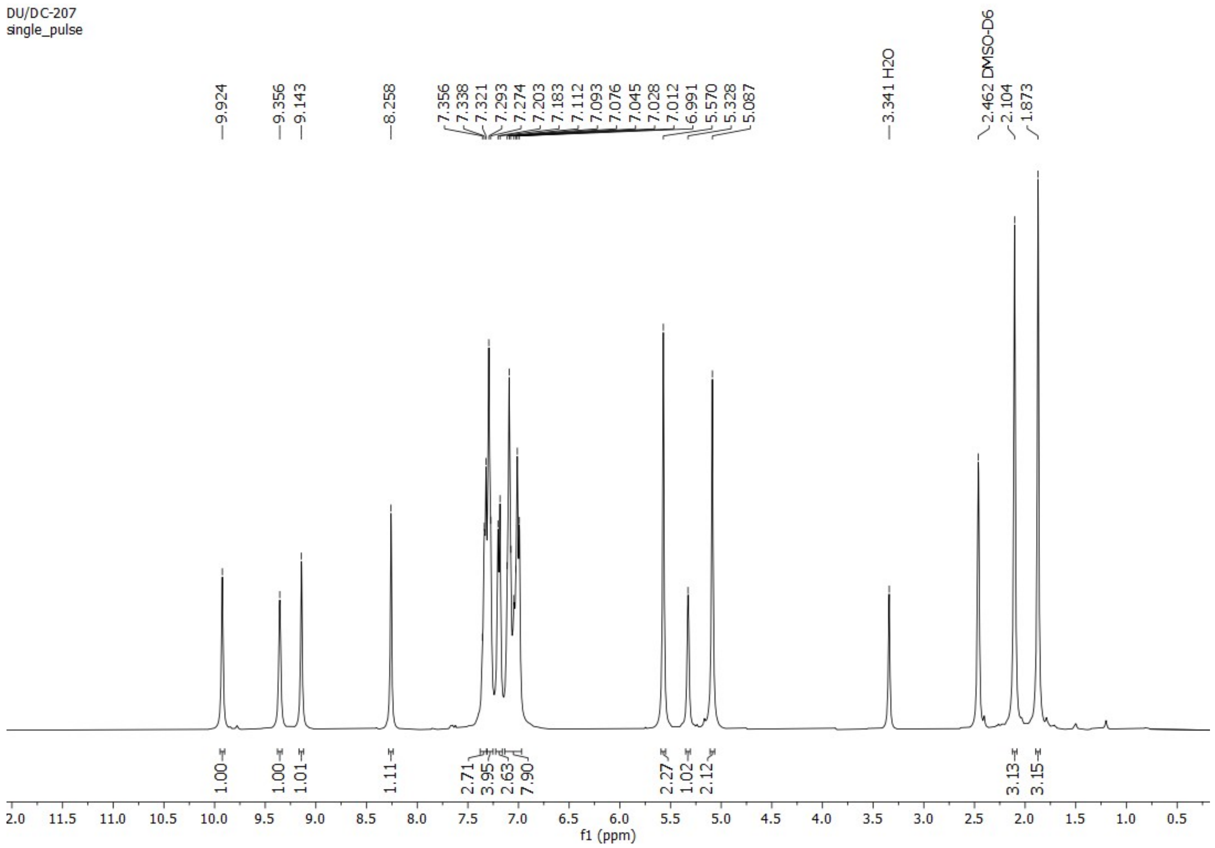

Figure S35. ^{1}H NMR spectrum of compound **4q** at 400 MHz in DMSO-d6

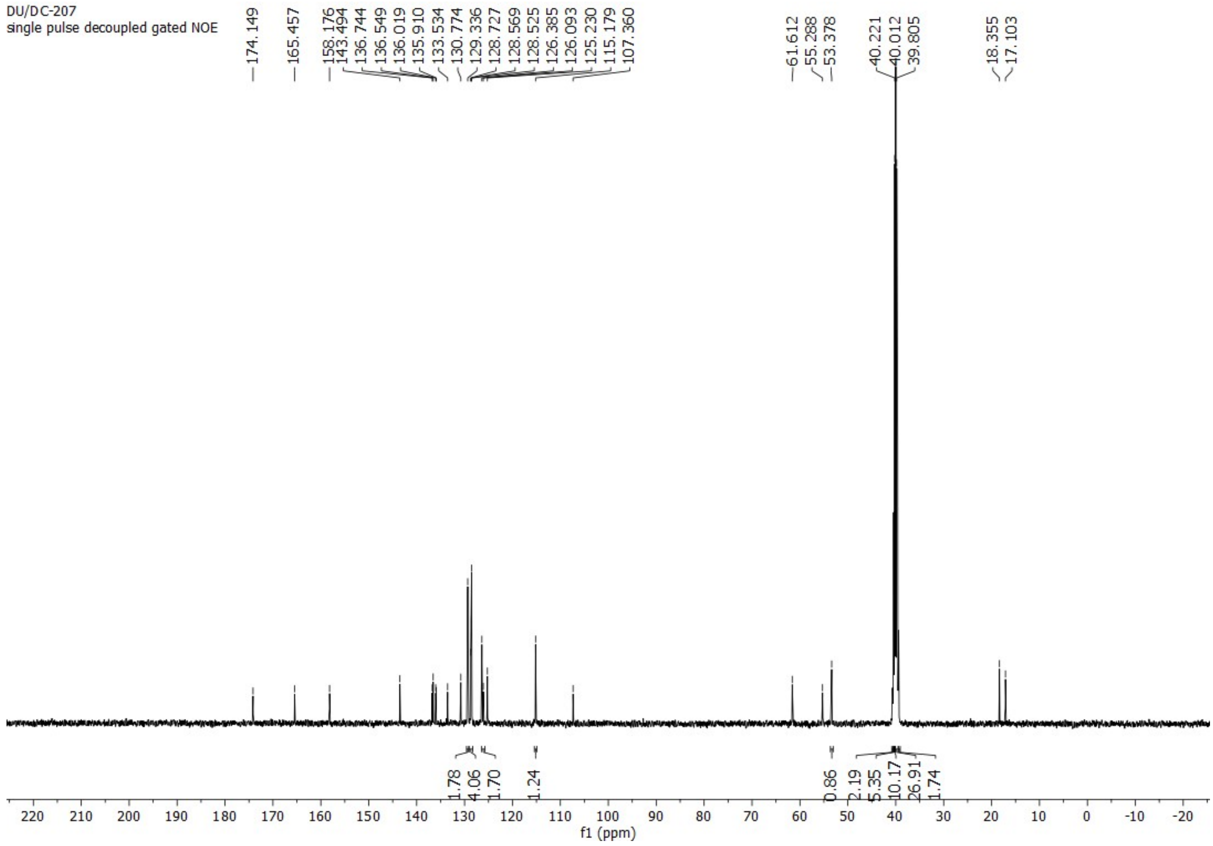

Figure S36. ^{13}C NMR spectrum of compound **4q** at 150 MHz in DMSO-d6

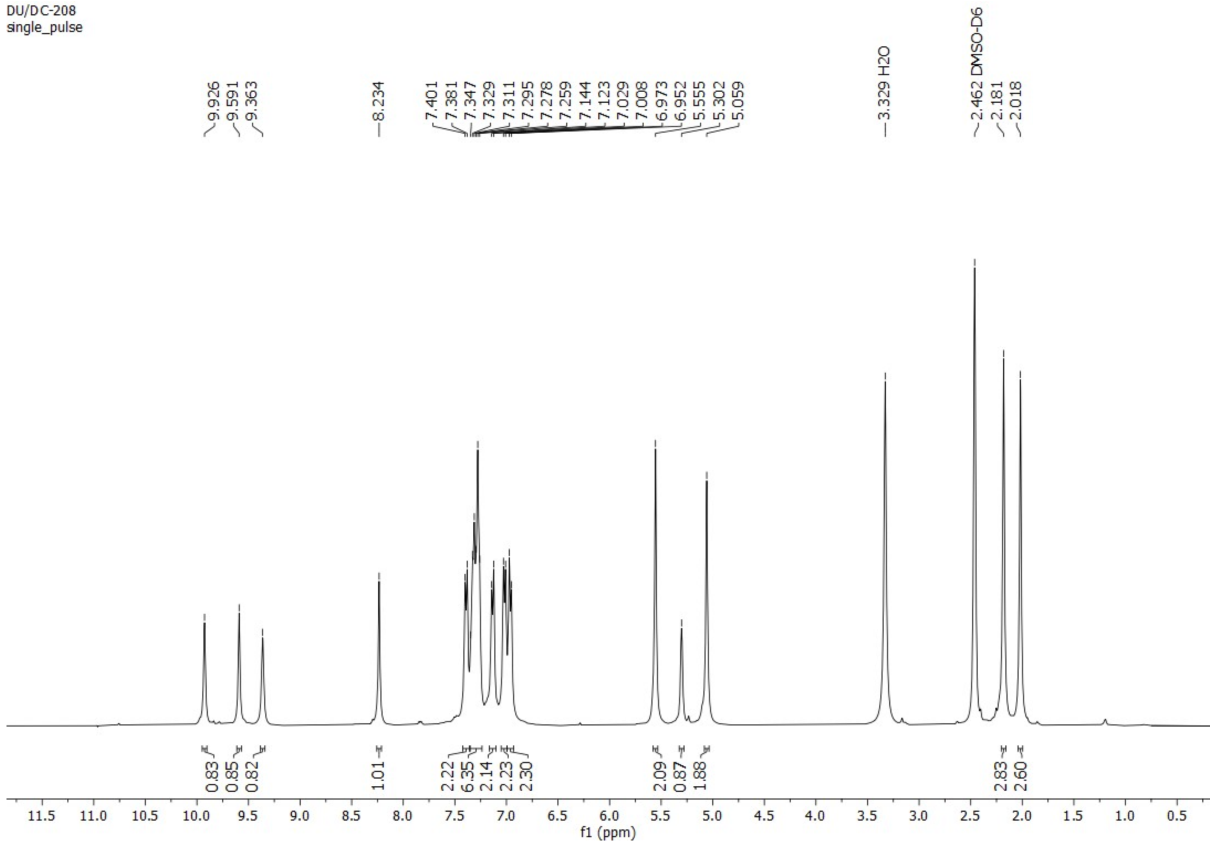

Figure S37. ^{1}H NMR spectrum of compound **4r** at 400 MHz in DMSO-d6

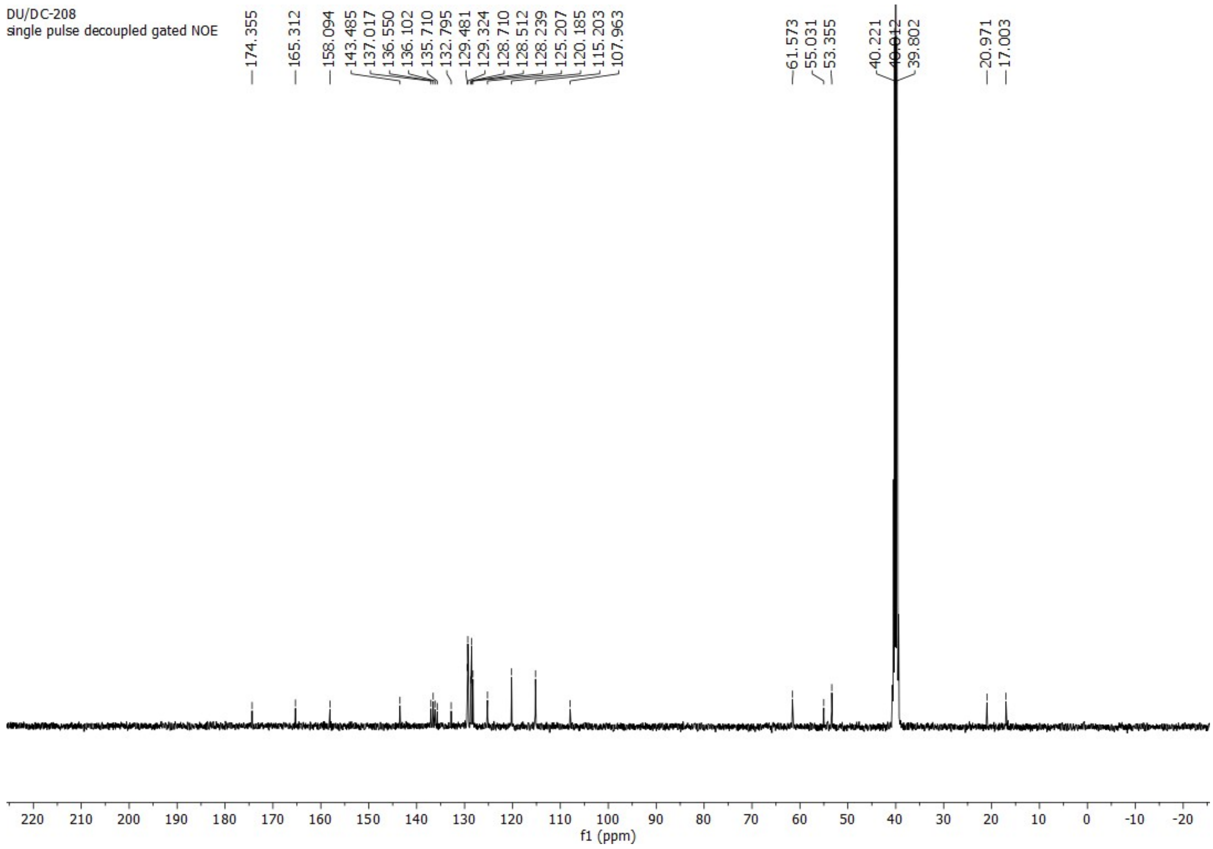

Figure S38. ^{13}C NMR spectrum of compound **4r** at 150 MHz in DMSO-d6

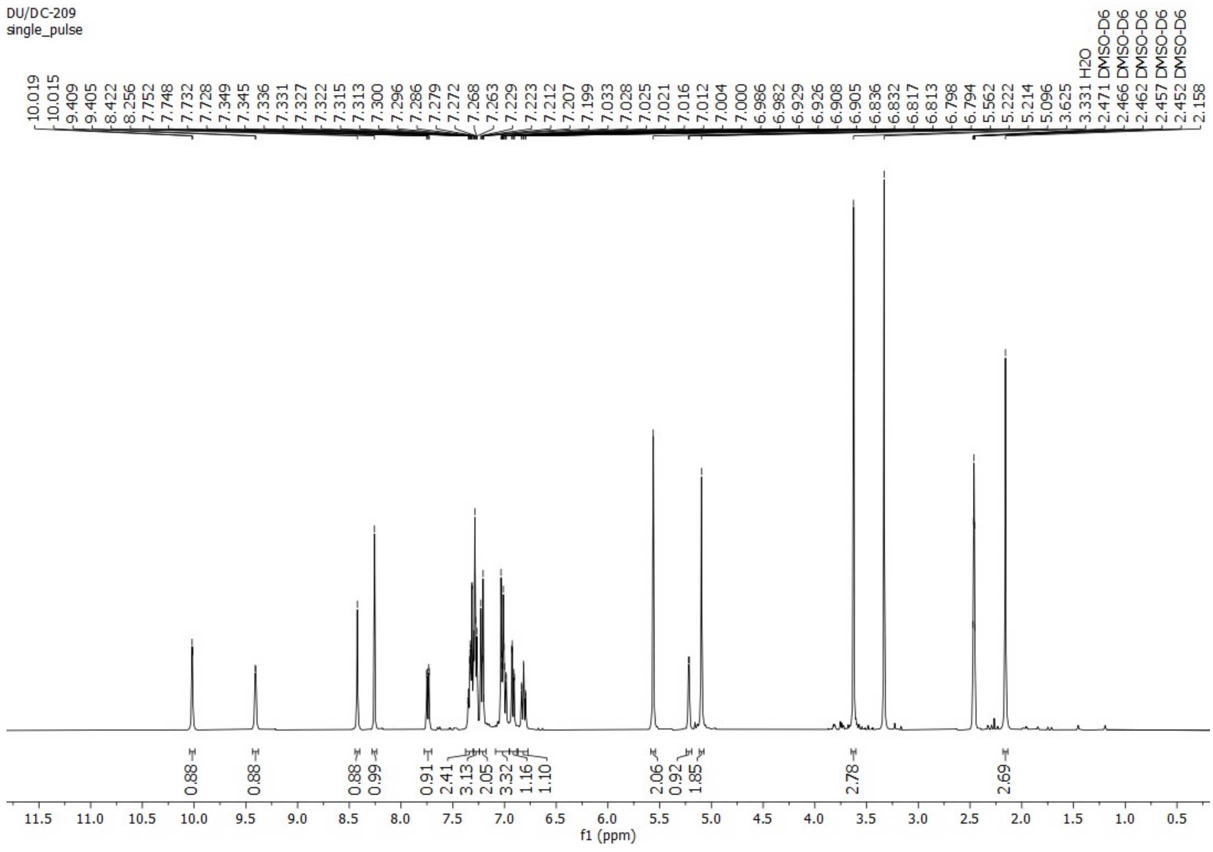

Figure S39. ^{1}H NMR spectrum of compound **4s** at 400 MHz in DMSO-d6

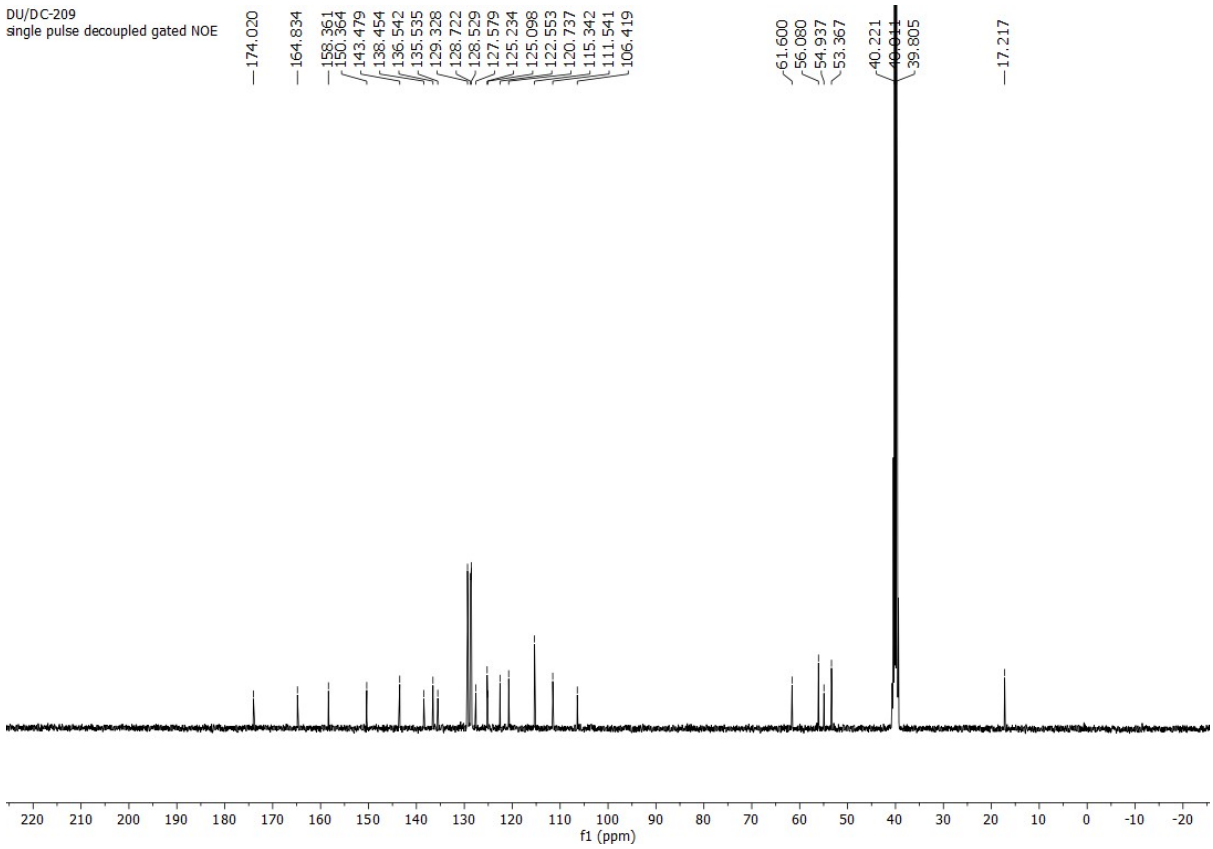

Figure S40. ^{13}C NMR spectrum of compound **4s** at 150 MHz in DMSO-d6

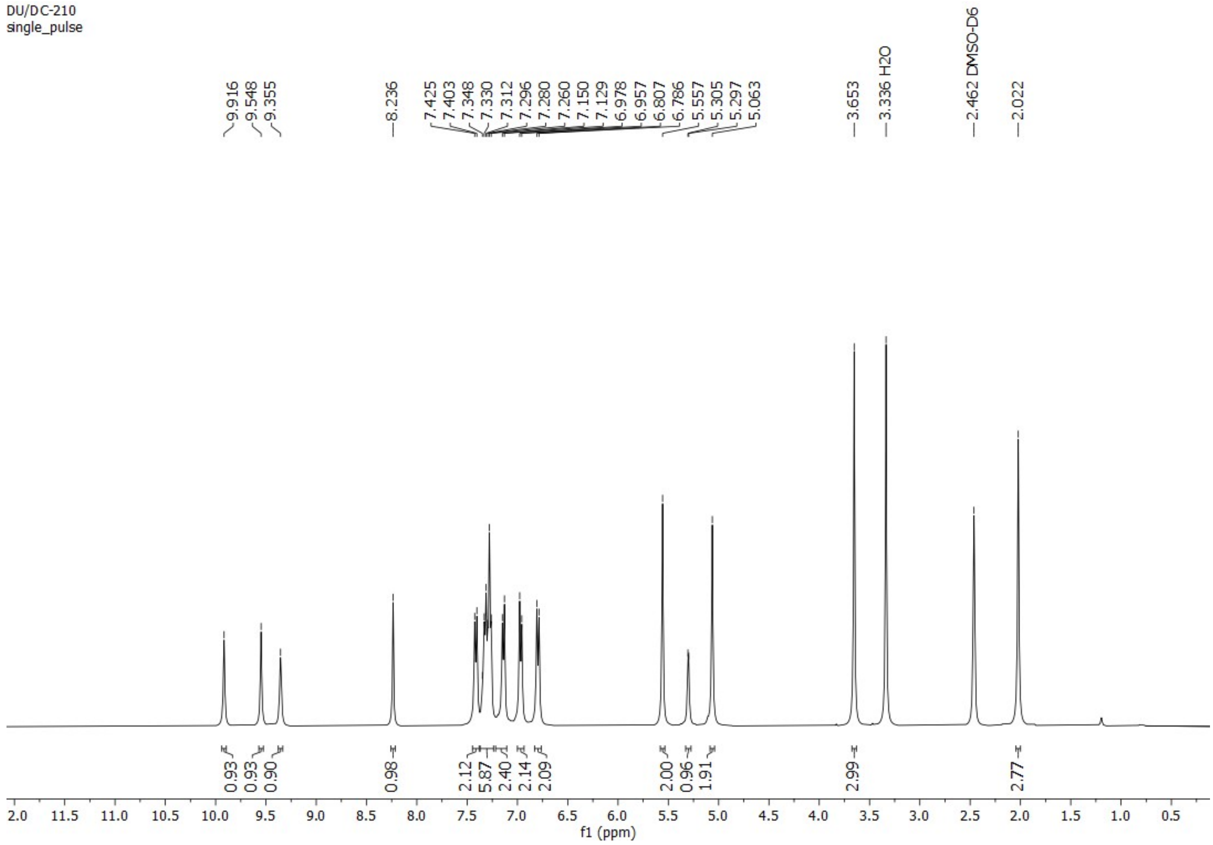

Figure S41. ^{1}H NMR spectrum of compound **4t** at 400 MHz in DMSO-d6

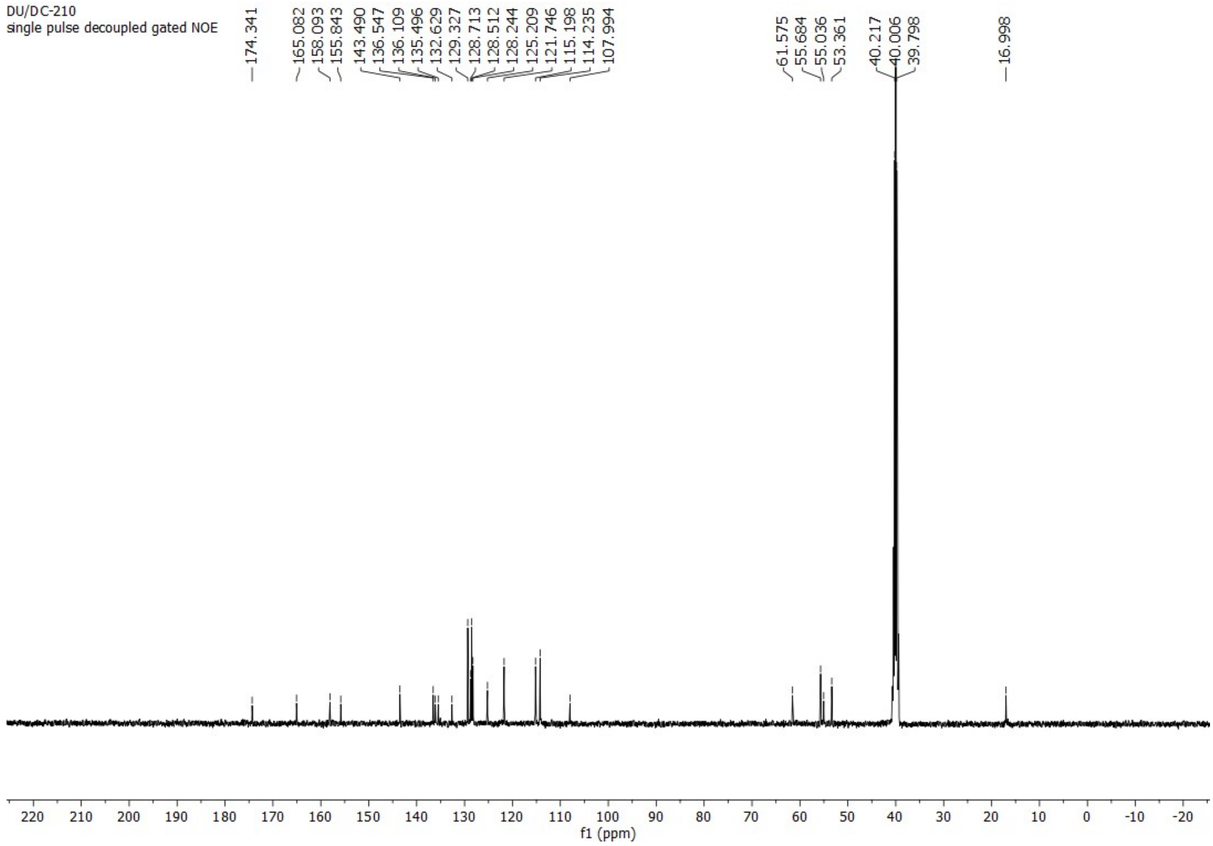

Figure S42. ^{13}C NMR spectrum of compound **4b** at 150 MHz in DMSO-d6

Mass spectra of **4a**-**4t**

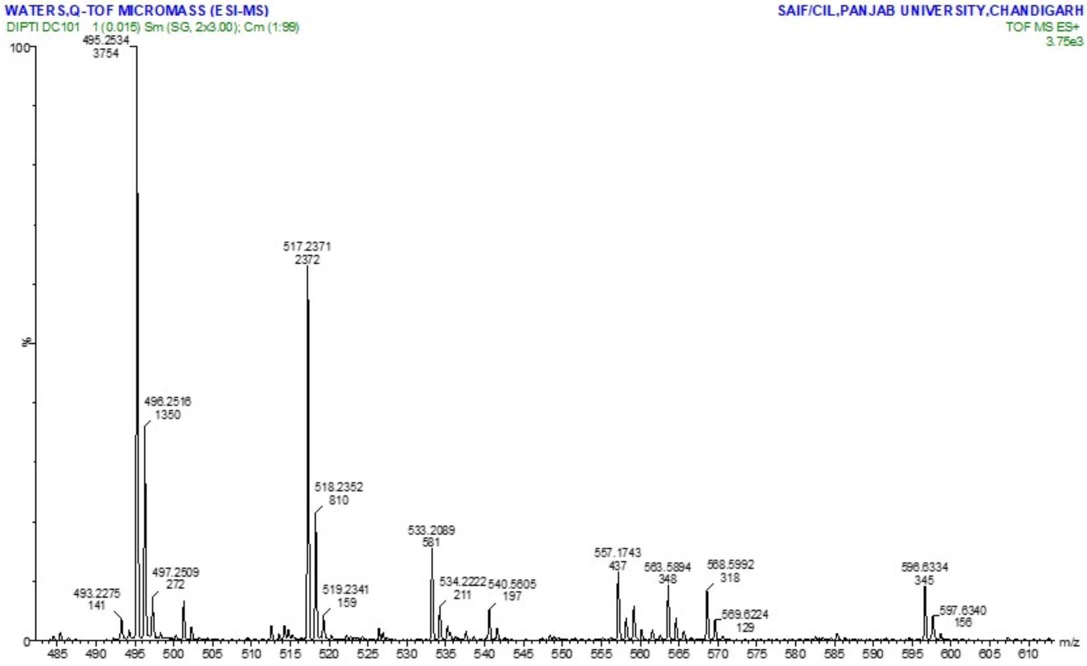

Figure S43. HRMS of compound **4a**

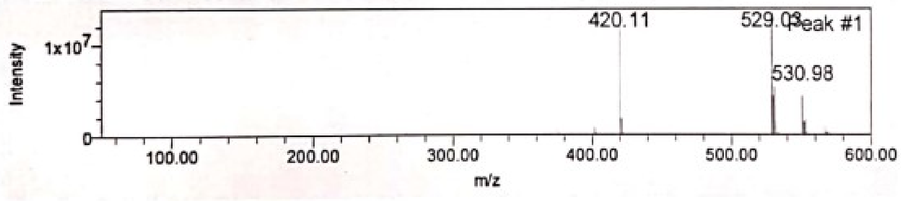

Peak Results

	RT	Area	% Area	Name	Base Peak (m/z)
1	20.009	337927508	100.00		529.03

Figure S44. LCMS of compound **4b**

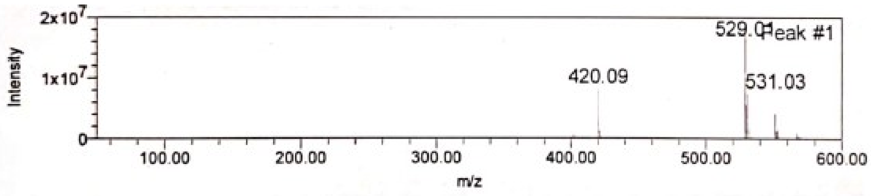

Peak Results

	RT	Area	% Area	Name	Base Peak (m/z)
1	21.057	391689307	100.00		529.01

Figure S45. LCMS of compound **4c**

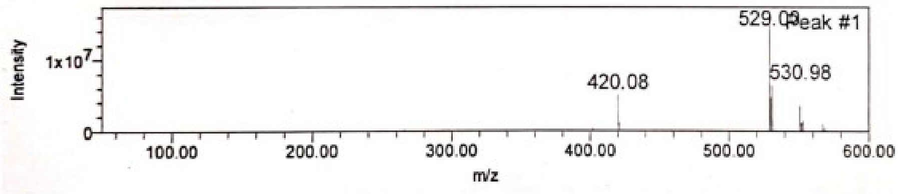

Peak Results

	RT	Area	% Area	Name	Base Peak (m/z)
1	21.080	281838188	100.00		529.00

Figure S46. LCMS of compound **4d**

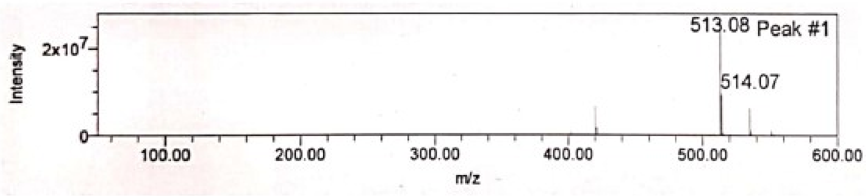

Peak Results

	RT	Area	% Area	Name	Base Peak (m/z)
1	19.502	378301089	100.00		513.08

Figure S47. LCMS of compound **4e**

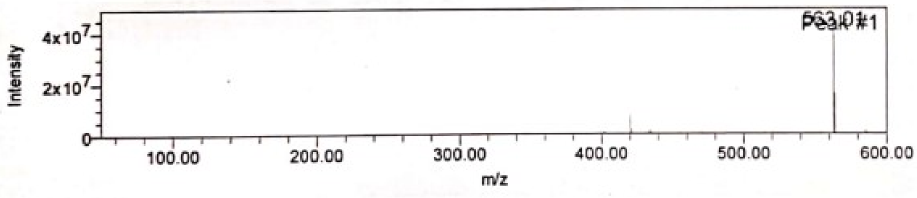

Peak Results

	RT	Area	% Area	Name	Base Peak (m/z)
1	21.897	541048205	100.00		563.01

Figure S48. LCMS of compound **4f**

Lamda:210 nm Analysed By:

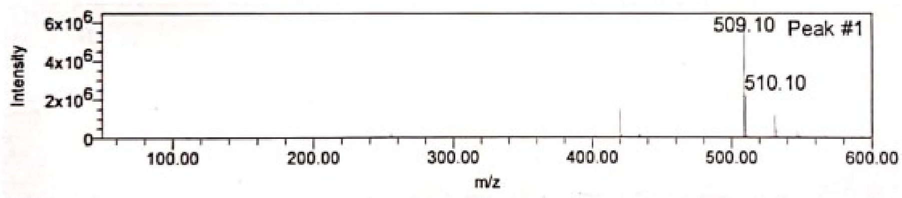

Peak Results

	RT	Area	% Area	Name	Base Peak (m/z)
1	18.903	69328238	100.00		509.10

Figure S49. LCMS of compound **4g**

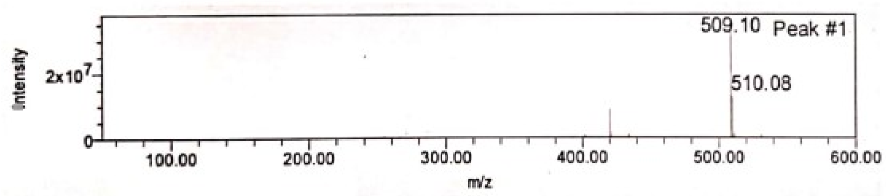

Peak Results

	RT	Area	% Area	Name	Base Peak (m/z)
1	20.018	771578598	100.00		509.10

Figure S50. LCMS of compound **4h**

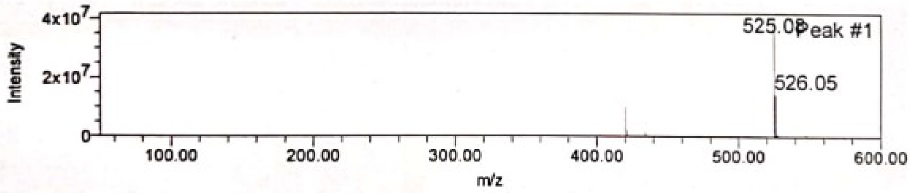

Peak Results

	RT	Area	% Area	Name	Base Peak (m/z)
1	19.640	575852929	100.00		525.08

Figure S51. LCMS of compound **4i**

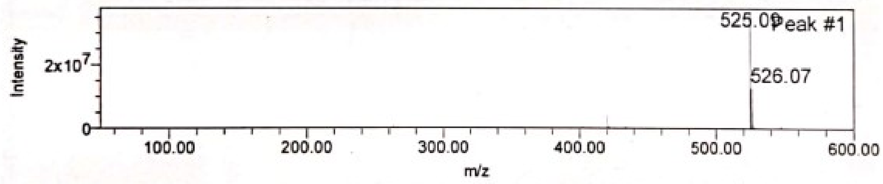

Peak Results

	RT	Area	% Area	Name	Base Peak (m/z)
1	18.485	373959080	100.00		525.09

Figure S52. LCMS of compound **4j**

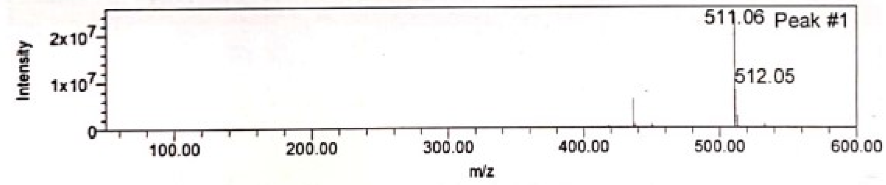

Peak Results

	RT	Area	% Area	Name	Base Peak (m/z)
1	20.433	466055335	100.00		511.06

Figure S53. LCMS of compound **4k**

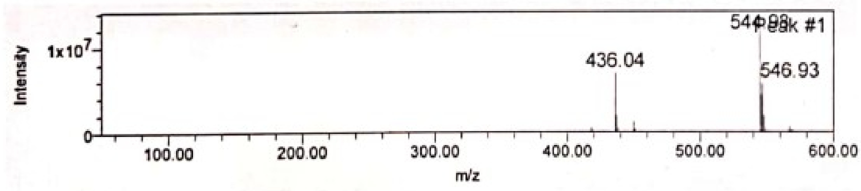

Peak Results

	RT	Area	% Area	Name	Base Peak (m/z)
1	21.348	373579212	100.00		544.98

Figure S54. LCMS of compound **4l**

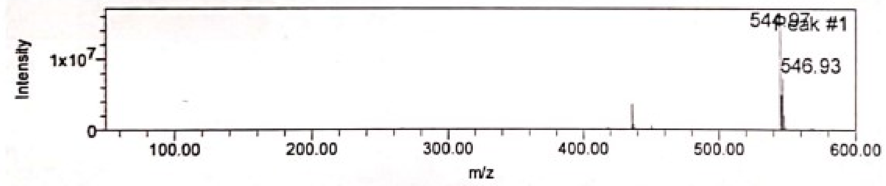

Peak Results

	RT	Area	% Area	Name	Base Peak (m/z)
1	22.557	537694879	100.00		544.97

Figure S55. LCMS of compound **4m**

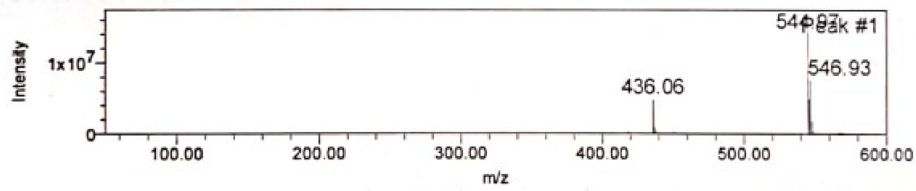

Peak Results

	RT	Area	% Area	Name	Base Peak (m/z)
1	22.376	366963147	100.00		544.97

Figure S56. LCMS of compound **4n**

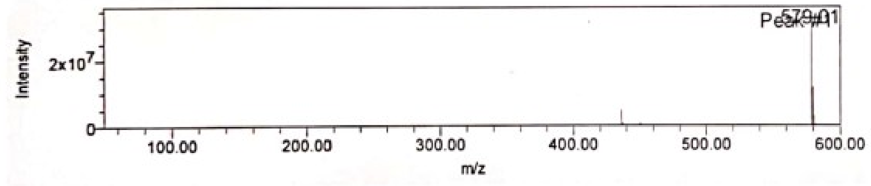

Peak Results

	RT	Area	% Area	Name	Base Peak (m/z)
1	23.250	463429659	100.00		579.01

Figure S57. LCMS of compound **4p**

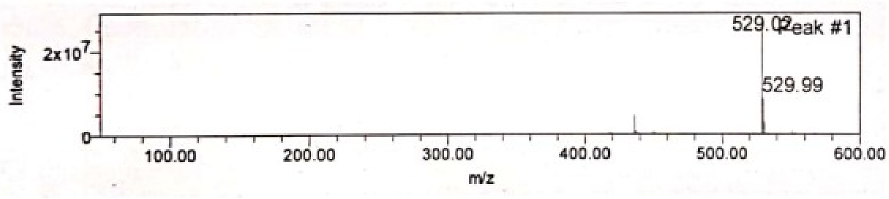

Peak Results

	RT	Area	% Area	Name	Base Peak (m/z)
1	20.750	444407778	100.00		529.02

Figure S58. LCMS of compound **4p**

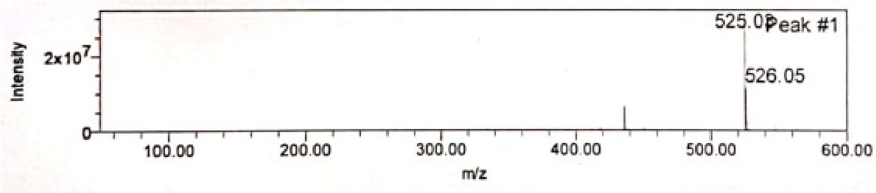

Peak Results

	RT	Area	% Area	Name	Base Peak (m/z)
1	20.321	534054667	100.00		525.03

Figure S59. LCMS of compound **4q**

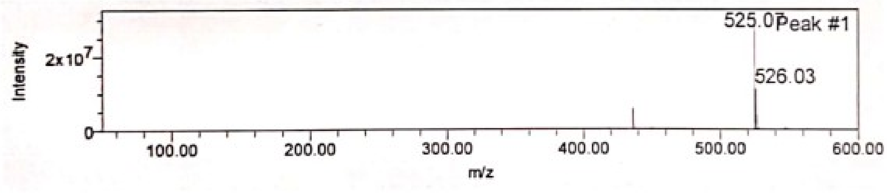

Peak Results

	RT	Area	% Area	Name	Base Peak (m/z)
1	21.454	557440886	100.00		525.07

Figure S60. LCMS of compound **4r**

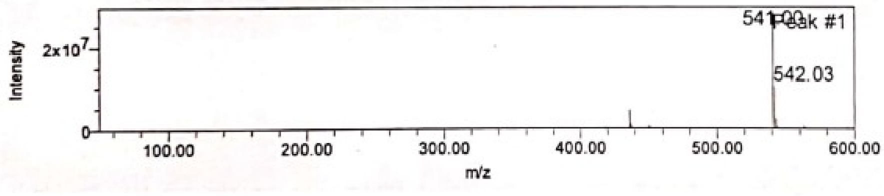

Peak Results

	RT	Area	% Area	Name	Base Peak (m/z)
1	21.074	758060040	100.00		541.00

Figure S61. LCMS of compound **4s**

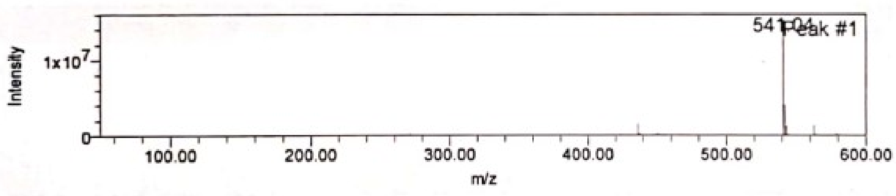

Peak Results

	RT	Area	% Area	Name	Base Peak (m/z)
1	19.844	139043859	100.00		541.04

Figure S62. LCMS of compound **4t**
